# Finding Primary Care—Repurposing Physician Registration Data to Generate a Regionally Accurate List of Primary Care Clinics: Development and Validation of an Open-Source Algorithm

**DOI:** 10.2196/34141

**Published:** 2022-06-22

**Authors:** Ian R Cooper, Cameron Lindsay, Keaton Fraser, Tiffany T Hill, Andrew Siu, Sarah Fletcher, Jan Klimas, Michee-Ana Hamilton, Amanda D Frazer, Elka Humphrys, Kira Koepke, Lindsay Hedden, Morgan Price, Rita K McCracken

**Affiliations:** 1 Innovation Support Unit Department of Family Practice University of British Columbia Vancouver, BC Canada; 2 British Columbia Centre on Substance Use Vancouver, BC Canada; 3 Faculty of Health Sciences Simon Fraser University Burnaby, BC Canada; 4 British Columbia Academic Health Sciences Network Vancouver, BC Canada

**Keywords:** physicians, primary care, primary health care, health services accessibility, practice patterns, physicians, physicians’ offices, computing methodologies, algorithms

## Abstract

**Background:**

Some Canadians have limited access to longitudinal primary care, despite its known advantages for population health. Current initiatives to transform primary care aim to increase access to team-based primary care clinics. However, many regions lack a reliable method to enumerate clinics, limiting estimates of clinical capacity and ongoing access gaps. A region-based complete clinic list is needed to effectively describe clinic characteristics and to compare primary care outcomes at the clinic level.

**Objective:**

The objective of this study is to show how publicly available data sources, including the provincial physician license registry, can be used to generate a verifiable, region-wide list of primary care clinics in British Columbia, Canada, using a process named the Clinic List Algorithm (CLA).

**Methods:**

The CLA has 10 steps: (1) collect data sets, (2) develop clinic inclusion and exclusion criteria, (3) process data sets, (4) consolidate data sets, (5) transform from list of physicians to initial list of clinics, (6) add additional metadata, (7) create working lists, (8) verify working lists, (9) consolidate working lists, and (10) adjust processing steps based on learnings.

**Results:**

The College of Physicians and Surgeons of British Columbia Registry contained 13,726 physicians, at 2915 unique addresses, 6942 (50.58%) of whom were family physicians (FPs) licensed to practice in British Columbia. The CLA identified 1239 addresses where primary care was delivered by 4262 (61.39%) FPs. Of the included addresses, 84.50% (n=1047) were in urban locations, and there was a median of 2 (IQR 2-4, range 1-23) FPs at each unique address.

**Conclusions:**

The CLA provides a region-wide description of primary care clinics that improves on simple counts of primary care providers or self-report lists. It identifies the number and location of primary care clinics and excludes primary care providers who are likely not providing community-based primary care. Such information may be useful for estimates of capacity of primary care, as well as for policy planning and research in regions engaged in primary care evaluation or transformation.

## Introduction

Improving access to primary care is on health agendas around the world [1]. This is likely linked to the finding that increasing supply of primary care physicians is associated with decreased mortality rates [2]. In Canada, primary care accessibility is a persistent challenge. The media regularly note a supposed family physician (FP) shortage [3] despite most provinces having the highest ever number of FPs per capita [4].

Primary care is the first point of access to the health care system and provides longitudinal, person-focused care for most care needs across the lifespan [5,6]. The majority of primary care in Canada is still delivered by FPs at community-based outpatient clinics [7]. Currently, systematically identifying clinics versus individual physicians is difficult. This may be due to the ongoing reliance on funding of the majority of primary care services via individual physician remuneration [7-9] versus using a centralized system of service delivery, as is more commonly seen with other social services, such as public schools.

Across North America, initiatives to transform primary care have addressed access to care by establishing primary care teams [7-11]. Coordinated, team-based care in a primary care clinic is recognized as a critical part of modernized care [12-15] and has begun to be seen in Canada. However, analyses of these transformations often still rely on using the individual physician as a unit of service delivery [16,17] despite indicators that FPs may work in multiple locations, in a combination of roles [18-20], and that clinic culture and organization may contribute to physicians’ behaviors that influence quality of care [21,22]. The primary care clinic, rather than the individual physician, is evolving as the main access point for many patients. Exclusive reliance on physician-centric metrics potentially fails to include the contribution of nonphysician team members to the accessibility and quality of patient care. Other regions have begun to frame descriptions of primary care features and outcomes using the clinic as the unit of analysis [8,23,24]. A comprehensive list of primary care clinic locations is necessary for the effective assessment of initiatives that aim to improve access and would provide a baseline from which to measure change. There is no complete list available in British Columbia; an environmental scan has provided a few partial lists of specific types of care provision (eg, locations funded by health authorities) and some local data sources relying on physician self-report and voluntary listing of clinics. Other health care regions face similar data challenges as well as a need to improve access to primary care [25-27].

The objective of this study is to develop and verify an algorithm that uses a continually updated, public listing of individual physician license registration addresses to create an accurate region-wide listing of clinic locations where primary care is delivered.

## Methods

### Overview

This study describes the development, application, and verification of an algorithm applied to a physician license registration list in order to reliably generate an accurate list of primary care clinics. The Clinic List Algorithm (CLA) was developed by the primary care Innovation Support Unit at the University of British Columbia as part of a larger project examining primary care capacity and access measurement.

### Study Setting

British Columbia, Canada, has a single-payer health system [7], and primary care is provided almost exclusively by FPs. In many regions, physicians must register with a licensing body (eg, provincial or territorial college of physicians and surgeons), declare their specialization qualification (eg, family medicine), and give an address at which they provide services [28]. These registrations may also include other useful information, such as date of graduation, medical school location, or additional demographic data. In Canada, registration lists are publicly available.

### Data Sources

We used the following publicly available data sources to create the CLA.

#### The Registry

The College of Physicians and Surgeons of British Columbia (CPSBC) physician registry is the base to which we will apply additional data sets, processing actions, and verifying actions, and it is referred to in this paper as the Registry. It is updated continuously, is publicly accessible online, and can be requested from the CPSBC in a more accessible format [29]. The version accessed for this study is from September 2020.

#### Additional Address Data

The BC Ministry of Health publishes a comprehensive list of regional health authorities, broken down into Community Health Service Areas (CHSAs) [30]. We incorporated CHSAs because related health profiles exist detailing a community’s demographic, socioeconomic, and health and disease status. DataBC geolocation services information was used to add longitude and latitude coordinates to addresses, in accordance with their terms of service in British Columbia [31].

#### Partial Lists of Specific Family Physician Workplaces

##### Walk-in Clinic, Urgent and Primary Care Centre, and Hospital Lists

These lists were accessed between September 2020 and March 2021. The BC Ministry of Health publishes a list of locations in British Columbia that “provide walk-in treatment services for people who have minor illnesses or injuries or injuries that do not require a visit to a hospital emergency department or an urgent care facility” [32]. Walk-in treatment services are a form of community-based primary care [33]. This walk-in clinic list is updated twice yearly, in December and June. The Ministry of Health also manages two other publicly available lists that identify all hospitals [34] and Urgent and Primary Care Centres (UPCCs) [35] in British Columbia. These are updated similarly to the walk-in clinic list. UPCCs have been available in British Columbia since 2019 and “provide a flexible resource to meet the urgent and primary health care need” in British Columbia [35].

##### Corrections Facilities List

We generated a list of corrections facilities in British Columbia from multiple sources of publicly available information. These facilities included federal and provincial institutions, as well as immigration and remand institutions. At these addresses, primary care services are delivered to people living in corrections facilities. This care is not accessible to members of the community.

##### Long-term Care Facilities List

We obtained a file of all registered long-term care facilities from the BC Office of the Seniors Advocate [36]. This registry is regularly updated and contains details for all BC long-term care homes in which there are publicly funded beds. Medical services provided in these locations require a patient to meet strict admission criteria and are not available to other community members.

### Variables

The outcome of interest was an accurate list of primary care clinics. A clinic was defined as a location where primary care services are delivered by at least one FP registered to practice in British Columbia. Clinic locations were derived from the addresses provided by physicians who registered for a license to practice. The CLA was used to translate the physician registration addresses to primary care clinic locations (see Results section).

### Key Informants

Members of the research team with intimate knowledge of the regional primary care system (RKM and IRC) were needed in helping to develop inclusion and exclusion criteria.

### Analytic Methods

**Figure 1:**
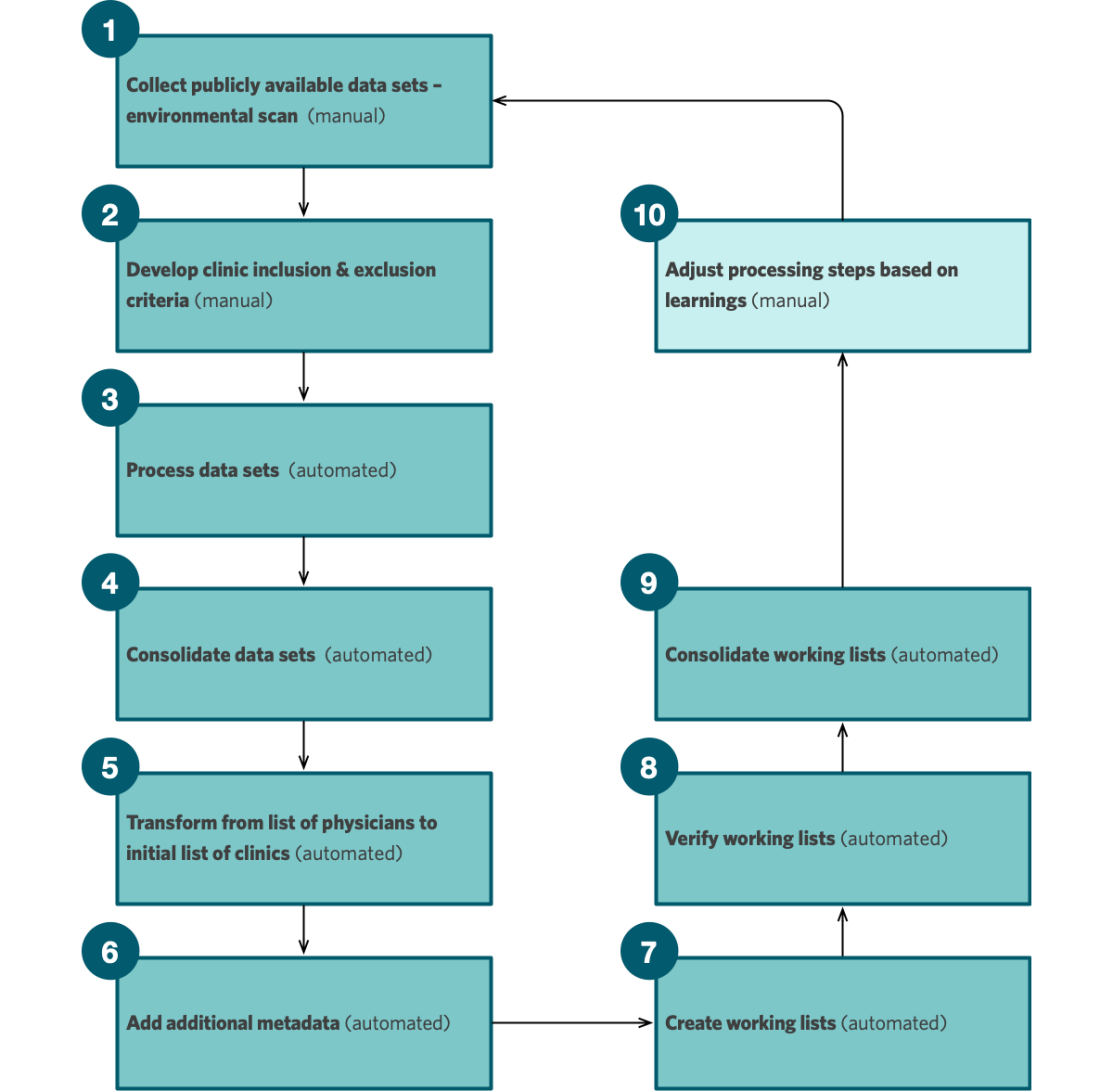
Clinic List Algorithm steps to generate the primary care location list using publicly available data sources.

The complete software code used for this algorithm is published online on GitHub [37]. The software was developed using Python (version 3.7.7; Python Software Foundation) in conjunction with the following open-source libraries: NumPy, pandas, OpenPyXL, GeoPy, and RegEx. Figure 1 summarizes the steps and flow developed for the algorithm application process, and Multimedia Appendix 1 includes additional details about how each step was completed. The Results section describes the application of the CLA. Descriptive statistics were calculated using Microsoft Excel for Mac (version 16.61).

### Ethics Approval

This study is exempt from Research Ethics Board review, as the data used for this research falls under the criteria outlined in Article 2.2 of the Tri-Council Policy Statement.

## Results

### Step 1: Collect Publicly Available Data Sets—Environmental Scan (Manual)

The data sources were collected and collated, including the Registry and additional address data. We plan to create regional service maps, and this level of location detail will facilitate that future work. Some addresses, almost exclusively post-office boxes, had to be reviewed individually and altered manually to allow them to be processed through the BC Address Geocoder. We then collected the following partial lists as sources for inclusion or exclusion: the walk-in clinic, UPCC, and hospital lists, which were accessed between September 2020 and March 2021, were generated; the corrections facilities list was generated; and the long-term care facilities list was requested.

### Step 2: Develop Clinic Inclusion and Exclusion Criteria (Manual)

We established inclusion and exclusion criteria for the type 2 secondary data sources. Walk-in clinics and UPCCs were included. Hospitals, corrections facilities, and long-term care facilities were excluded. Key search terms were developed from author knowledge (RKM and IRC) and grey and published literature searches. Search terms are shown in Table 1.

**Table 1:** Search terms used in filtering and sorting addresses from the Registry list of the College of Physicians and Surgeons of British Columbia to each of our working lists.

Criteria and working lists^a^		Search terms^b^
**Inclusion criteria**		
	Walk-in clinic	No search terms: list developed from walk-in clinic list
	Urgent and primary care	No search terms: list developed from Urgent and Primary Care Centre list
	Family	Family and (Med* or Clinic or Centre or Center or Associate* or Care or Practice)
	First Nations	First Nation* or First People* or Native or Aboriginal or Indigenous or {clinic-specific name}
	Clinic or center	Clinic* or Associate* or Center or Centre of Practice or Doctor*
**Exclusion criteria**		
	Hospital	Hosp*: list developed from hospital list
	Long-term care	Lodging or Lodge* or Manor or Senior or ALC^c^: list developed from long-term care list
	Corrections	Correction* or Immigra* or Pretrial or Custody or Institution or Detention or Holding or Healing Village: list developed from corrections list
	Sexual health	Sexual or STI^d^ or STD^e^ or {clinic specific name}
	Women’s health	Wom[a,e]n* or Menopause or Matern* or Birth* or Obstetric* or Gyne*
	Virtual	{organization-specific name} or Virtual or E[-]Health or Tele* or I[-]Health
	Administrative	Airport or Consulting or Admin* or Fraser Health Authority or First Nation* Health Authority or Coroner or CPSBC^f^ or College of Physician* and Surgeon* or Health Canada or VCH^g^ or Worksafe or Worksafebc or Worker* Comp* or BCAA^h^ or Veteran* Affair or RCMP^i^ or Air Canada or Quality

^a^Working lists created in step 7 of the Clinic List Algorithm.

^b^For search terms, the asterisk (*) denotes that any character following the search term was allowed, round brackets signify the inclusion of any number of terms inside those brackets, square brackets indicate that any of the letters inside the brackets were allowable in the word, and curly brackets identify a search term specific to a clinic that is not a generic term and is not included in this list. Regular expression statements used in the algorithm code are found in Multimedia Appendix 2.

^c^ALC: alternate level of care.

^d^STI: sexually transmitted infection.

^e^STD: sexually transmitted disease.

^f^CPSBC: College of Physicians and Surgeons of British Columbia.

^g^VCH: Vancouver Coastal Health.

^h^BCAA: British Columbia Automobile Association.

^i^RCMP: Royal Canadian Mounted Police.

### Step 3: Process Data Sets (Automated)

**Figure 2:**
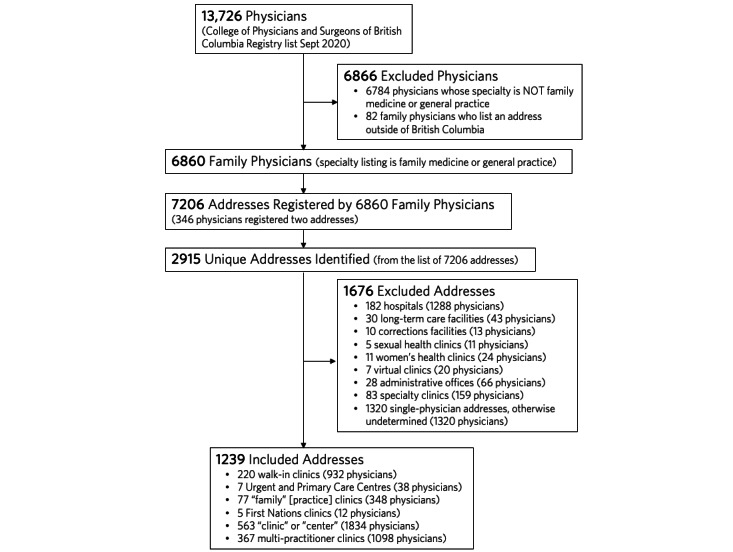
Case study results identifying included, excluded, and undetermined addresses of primary care locations in British Columbia.

The software filtered the CPSBC Registry list using the Specialties and Certificates column to include only FPs. The CPSBC Registry list contained 13,726 physicians, of which 6942 were identified as FPs. We removed addresses from outside British Columbia (n=82), leaving 6860 FPs (Figure 2). These 6860 physicians were found to have 7206 registered addresses. All remaining address fields in the Registry list were then standardized.

### Step 4: Consolidate Data Sets (Automated)

Additional address data were merged with the Registry list, appending geolocation coordinates and relevant CHSA data fields to each entry using consistent data fields between data sources.

### Step 5: Transform From List of Physicians to Initial List of Clinics (Automated)

The software identified 2915 unique addresses from the Registry list. All 6860 FPs were then assigned to the unique addresses, creating the initial clinic-centric list.

### Step 6: Add Additional Metadata (Automated)

Metadata were added as new variables by the software to include the number of FPs identified at each clinic, the rurality of clinic locations, key term identification, and overall summaries of each process undertaken by the software.

### Step 7: Create Working Lists (Automated)

The inclusion and exclusion criteria developed in step 2 were applied to create the 15 working lists. The results of this step are depicted in Figure 2. The partial lists’ data sources were applied to identify addresses of walk-in clinics, UPCCs, hospitals, corrections facilities, and long-term care facilities and then sorted to their respective working lists. Key search terms in the address fields of each remaining listing were sorted to respective working lists (Table 1). Finally, unique addresses with more than one associated FP and that were not sorted by any previous step were assigned to the multi-practitioner working list. Single addresses listed by only one FP were assigned to the single-practitioner working list.

### Step 8: Verify Working Lists (Manual)

The verification process was completed manually, using the working lists produced in step 7. The goal of verification was to determine if an address assigned to a particular working list had been correctly included or excluded as a community-based location that provided any primary care.

The sample size for verification was determined for each separate working list. The proportion that was verified differed between working lists. Considerations in verification were (1) baseline confidence that the sorting method would accurately include and exclude addresses and (2) time and resources available for the verification process. Multimedia Appendix 3 includes the proportion of addresses verified for each group.

The verification process was applied to each working list and consisted of at least one of four reviews. In the first review, lists prepared by government organizations were assumed to be accurate, and no additional verification was done. The second review was manual and was performed by an expert informant (RKM) who personally knew providers or clinics that were delivering primary care. The third and fourth reviews were done by KF and IRC and began with an internet search for clinic name, address, and FP names. If the internet search was insufficient to ascertain that primary care was being delivered at that location, a final, most resource-intensive level of review was done by phoning the clinic.

The internet search process and script used for phone call verification is included in Multimedia Appendix 4. A team member (IRC) collated responses from all four types of reviews and compared a subset of completed entries to ensure consistency between the methods of verification.

The denominator for verification accuracy was the number of addresses in the step multiplied by the proportion to be verified. If a verified address was included—or excluded, depending on the specific working list—as a primary care location, it contributed to the numerator. This gave a relative accuracy for the groups used in the algorithm to identify inclusion or exclusion of potential primary care clinics.

Confirmation of primary care service provision was not possible for the virtual working list, as the six physical addresses correctly identified with this filter were for administrative offices only. For the remainder of the working lists, verification was completed for almost all potential clinics found, with two notable exceptions. The family working list verification was completed on only 27% (21/77) of the clinics identified because this filter was established late in our development process as a very high-fidelity filter. The single-practitioner working list verification was completed on only 15.98% (211/1320) of the addresses identified; of these 211 addresses, only 118 (55.9%) were currently providing primary care. Initial work to develop patterns to identify clinics from the single-practitioner working list indicated that this working list was nonspecific to primary care clinics (eg, contained many residential addresses).

### Step 9: Consolidate Working Lists (Automated)

The software merged the working lists into one final clinic list that is presented in Multimedia Appendix 5. From the original 2915 unique addresses identified, the CLA excluded 356 (12.21%) addresses, representing 1624 FPs. The CLA also defined 1320 addresses as single-physician addresses that were not identified through any other filter. The CLA identified 1239 potential primary care clinics representing 4262 FPs (Figure 2). Table 2 shows the geographic and descriptive data about included primary care clinics.

**Table 2:** Geographic distribution and descriptive statistics of included primary care clinic addresses from the case study in British Columbia.

CHSA^a^ urban or rural classification^b^	Unique addresses^c^ (n=1239), n (%)	FPs^d,e^ with addresses in this region (n=4262), n (%)	FPs per unique address	
			Median (IQR)	Range
Metropolitan	566 (45.68)	1897 (44.51)	2 (2-4)	1-20
Large urban	189 (15.25)	647 (15.18)	3 (2-4)	1-14
Medium urban	195 (15.74)	717 (16.82)	3 (2-5)	1-23
Small urban	99 (7.99)	410 (9.62)	3 (2-5)	1-21
Rural hub	75 (6.05)	269 (6.31)	3 (2-5)	1-15
Rural	102 (8.23)	299 (7.02)	2 (2-3)	1-15
Remote	12 (0.97)	23 (0.54)	2 (1-2)	1-5
Total	1239 (100)	4262 (100)	2 (2-4)	1-23

^a^CHSA: Community Health Service Area.

^b^Urban and rural classifications are based on the classification of the CHSA by the BC Ministry of Health [30].

^c^The software from the Clinic List Algorithm (CLA) was used to identify the unique addresses that were included, with FPs from the College of Physicians and Surgeons of British Columbia list.

^d^FP: family physician.

^e^These are FPs who are registered with the CPSBC and have listed an address identified by the CLA as a primary care location.

### Step 10: Adjust Processing Steps Based on Learnings (Manual)

Each iteration of the working lists was reviewed by the study authors (IRC, CL, KF, TTH, SF, JK, MAH, and RKM). Three revisions of the software were made to address important learnings (eg, adding the term “family” as an inclusion search term and obtaining the complete list of long-term care addresses). The new rules and processes created as a result of discoveries in the verification process were incorporated into the final code, which is found on GitHub [37].

## Discussion

### Principal Findings

The CLA identified 1239 addresses where primary care was delivered by 4262 FPs from a list of the 6942 FPs licensed to practice in British Columbia. The algorithm used publicly available data sources and could be repeated as the data sources are updated. The CLA was developed to facilitate the study of regional effects of policies to transform primary care, using the clinic as the unit of analysis. This may be of particular value in regions where such lists do not exist or are not publicly available.

Previous work exploring geographic distribution of primary care services also used physician registration addresses to find locations of primary care service delivery [25,26]. However, that work did not include a way to remove the physicians who are unlikely to be providing community-based services. Modern FPs have dynamic and varied practice patterns that rarely are in a single location and providing only community-based primary care [20,38]. The CLA allows for observations about how FPs are organizing their work. We identified 1624 FPs who have an address at which primary care services are not available. While these physicians are likely engaged in important health services work that uses their valuable time and skills, they should not be assumed to be a potential source for primary care [18,38,39].

In our final clinic list, 94.25% (4017/4262) of included FPs shared a registered address with at least one other person. While this may not reflect the complete practice context (eg, whether the practice is organized as a team) and does not include the undetermined single-practitioner working list (n=1320 FPs), it does confirm that most FPs are not working in solo practice settings. The CLA allows for the creation of a unique physician identifier that can be associated with an address. Appropriately constructed research and quality initiatives could use such a variable to study physician practice patterns, identified in administrative data, as mapped to physical locations [8,14,17,40]. In many Canadian regions, this linkage has not previously been possible [5,19,41,42].

The majority of primary care evaluation and research on access and quality of care has needed to rely on the individual FP as the unit of analysis [19,41,43-45] (eg, patient attachment, third next available appointment, and rates of opioid prescribing by a single FP). This does not appear to represent how the majority of FPs are actually practicing. Measurements that focus on the clinic as the unit of analysis for primary care provision would likely better reflect the changing practice context in primary care and allow for accurate health resource planning [8,14,40,46].

### Limitations and Future Work

We cannot assume that each practitioner works at each location full time, providing only primary care services, because FPs often work in multiple locations [38]. Further work is required to understand patient access and attachment capacity at each clinic location [47,48]. We assumed that a physician registers all addresses where they provide any care at the time of their license registration. It is possible that this is not the case and that a physician registers an address for convenience or a personal reason. This could result in undercounting of physicians working at a location or failure to identify a primary care location at all.

We neither called nor visited every address to verify their services, due to limited time and resources. We verified only 15.98% (n=211) of the addresses on the single-practitioner working list of 1320 unique addresses held by one FP. Future projects could include a more inclusive verification process for the single-practitioner working list. However, this work would need to be completed manually, given the lack of patterns we were able to discover in software development with the CLA. Future versions of the CLA should incorporate verification from previous versions, thus limiting the manual verification required for updating the final clinic list.

Two new primary care clinics in British Columbia are led by nurse practitioners (NPs) without FPs [49] and are, therefore, missing from the present clinic list. Additionally, rural and remote communities have clinics that may be serviced periodically by FPs, NPs, or registered nurses. It is unlikely that these locations were captured by the CPSBC Registry list. Should these types of clinics become more prevalent, the potential remedy for future versions of the final clinic list would be to apply a similar algorithm to registration lists of NPs and primary care registered nurses. Listing a hospital address was an exclusion criterion for the CLA. It is possible that in some settings, particularly rural areas, primary care is being provided at a hospital address. Future projects should verify if primary care is provided at the hospital address or if an FP registered with the CPSBC uses only a hospital address.

The utility of any registry of addresses relies on the currency and accuracy of the listing [48]. The CLA will require updating; fortunately, the physician registration list is updated continually by the provincial licensing body, and with appropriate resources, the algorithm can be repeated to update the accurate list of clinics.

Finally, the rapid growth of primary care services offered through virtual platforms introduces an important new element to this work [42,50]. FPs can now provide care to patients in distant locations. In British Columbia, virtual care via the single-payer system requires a physician to be registered with the CPSBC [51]. At this time, it is unclear how virtual services might impact the CLA.

### Conclusions

The CLA can reliably create a list of primary care clinics based on publicly available information. The algorithm can be applied in other regions that need comprehensive lists of primary care clinics. The CLA offers researchers, decision-makers, and other organizations interested in health services a reliable way to estimate the regional distribution of primary care clinics. Future research could include the application of the CLA to the evaluation of initiatives for primary care transformation.

## Appendix

Multimedia Appendix 1 Detailed description of the application of the Clinic List Algorithm (CLA) method.

Multimedia Appendix 2 Regular expression statements used in the identification and sorting of addresses from the College of Physicians and Surgeons of British Columbia list to each of the groups.

Multimedia Appendix 3 Accuracy of algorithm sorting of individual physician addresses from licence registration to identify physical locations of primary care clinics.

Multimedia Appendix 4 Internet validation process and phone call script.

Multimedia Appendix 5 Clinic list.

## Abbreviations

CHSA: Community Health Service Area
CLA: Clinic List Algorithm
CPSBC: College of Physicians and Surgeons of British Columbia
FP: family physician
NP: nurse practitioner
UPCC: Urgent and Primary Care Centre
