# Supplementary material for: Finding Primary Care—Repurposing Physician Registration Data to Generate a Regionally Accurate List of Primary Care Clinics: Development and Validation of an Open-Source Algorithm
Source: JMIR Form Res. 2022 Jun 22;6(6):e34141. doi: 10.2196/34141 (PMC9496812; doi:10.2196/34141)
Supplement: Multimedia Appendix 1 [file formative_v6i6e34141_app1.pdf]

Multimedia Appendix 1: Detailed Description of Application of the CLA Method (Development and Testing of the CLA Algorithm outlined in fig 1)

Step 1: Collect Publicly Available Data Sets – Environmental Scan (Manual)

We identified all additional potential sources of addresses for individual primary care providers, locations where primary care may be delivered and/or locations where primary care providers (eg, FPs) work but do not provide community-based outpatient primary care. This was accomplished through consultations with key informants, a grey literature search and targeted reviews of government, Regulatory College, physician association and/or health insurance sources. Potential data sources were assessed for completeness, accessibility, currency and frequency of update and whether they contain data elements of interest.

This Environmental Scan identified two kinds of secondary data sources:

a. Additional address data (Type 1) – Data sources that provide additional geographical descriptions for physical addresses. A useful list included a mechanism to match with the Registry (eg the postal code of the provider address can be matched to a postal code that is within a specific health authority region).

b. Inclusion or exclusion (Type 2) – Data sources that could inform inclusion or exclusion of specific addresses in the other data sets. For example, a list of walk-in clinics can be used to include matching addresses, as a source of primary care. Similarly, a list of hospitals can be used to exclude addresses for FPs who use the hospital address (assumes they work in hospital only).

Step 2: Develop Clinic Inclusion & Exclusion Criteria (Manual)

We established the precise definition for the type of clinic that is being searched for. Because our outcome is a list of primary care clinics, we began with defining a primary care clinic as a location where patients can be seen for general health complaints, without a referral, and have the visit covered by the single-payer provincial medical services plan. This includes clinics that may have some restrictions (eg, require a private pay membership, or patients having a specific diagnosis such as HIV). This is consistent with the Canadian literature [52].

We developed a list of regionally specific key terms that could potentially be used to identify clinic type. Key search terms (eg, "family practice" – inclusion or "esthetics" – exclusion) are identified using key informant knowledge of the primary care environment, existing literature and any other sources (eg, previous local quality improvement initiatives).

Step 3: Process Data Sets (Automated)

Address fields were standardized and cleaned in each data set. (Capitalization of all letters, transformation of address data fields and postal codes to a standardized format). CLA steps 4–7 will work only with standardization. This step also includes initial removal of any unwanted listings, such as physicians with other licensed specialties such as "general surgery" or "obstetrics and gynecology."

Step 4: Consolidate Data Sets (Automated)

The Registry list was merged with the Type 1 secondary data sets, using consistent fields between them (eg, postal codes). In cases where the same fields are not present in both lists, additional data may be required to merge data sets. For example, the postal code data field is present in the Registry, but not in a secondary list. However, in the secondary list other

geographic data fields are present that could be used to identify postal code (eg, street address). This information is typically publicly available or easily accessible on request from the organizations that create the secondary lists.

Step 5: Transform from List of Physicians to Initial List of Clinics (Automated)

The Registry list was transformed from a physician-centric list to an address-centric (ie, clinic-centric) list through a two-step process:

1. Removing duplicate addresses – Using the most precise data fields available that are consistent between entries (eg, geocodes), the first address entry available on the list was kept and all subsequent entries removed. All additional information from these subsequent entries was appended to the first unique entry. It is important to be as specific as possible in this step, as different unit numbers within the same building are often distinct clinics.
2. Map providers to addresses – Using the list of unique addresses created as a dictionary, each physician (based on standardized address) was assigned to the appropriate entry. This generated a final, clinic-centric list of primary addresses from the Registry.

Step 6: Add Additional Metadata (Automated)

Metadata were generated and appended as new variables to each clinic-centric list entry to enhance the data set. These metadata can include counting the number of providers at each address or determining the rurality of the location, for example. (Metadata variables were dependent on the data present in the Registry list and secondary data sources).

Step 7: Create Working Lists (Automated)

Using the inclusion and exclusion criteria developed in Step 2 , the clinic-centric list from step 6 was broken into a collection of working lists. Each separate processing step created a working list to be verified. Each address assigned to a separate working list was assigned a unique clinic identifier that included an identifier for the working list that it belonged to. There were four processes to sort addresses into working lists in this step:

1. List probable primary care clinics – This process includes addresses (eg, walk-in clinics), using Type 2 secondary lists that reliably identify primary care clinics. It then placed them into a working list of probable primary care clinics.
2. List non-primary care clinic locations – This process excludes addresses identified, using Type 2 secondary lists, as likely *not* primary care clinics (eg, long-term care facilities, hospitals). This was a repetition of the preceding step and created working lists of locations that are likely not primary care clinics.
3. Apply search terms – Searched addresses to identify those that contained search terms in address fields. Addresses found to contain a key term were separated into working lists based on categories of these key terms (eg, speciality clinics or administrative offices).
4. Identify number of practitioners – All remaining address entries that had not already been processed to a working list by one of the preceding steps were now divided into one of two lists. Entries where more than one practitioner shares the same address were processed into a Multi Practitioner working list for verification as included potential primary care clinics. All entries where only one

practitioner is present at an address were processed into a Single Practitioner working list.

Step 8: Verify Working Lists (Manual)

Each working list needed review and application of a list-specific verification process. The verification process ensured that addresses presumed to be a clinic providing primary care were correctly identified and addresses that were excluded did not deliver primary care. The verification process included: assumption of accuracy by original list provider (in the case of Type 2 secondary lists), consultation with a key informant who has knowledge of primary care in the region, internet searches for clinic website information and phone calls directly to listed numbers. Addresses verified to be primary care clinics were assigned an inclusion identifier. Those found not to be primary care were assigned an exclusion identifier. A CLA accuracy rate was calculated for each working list, identifying steps/processes that are most and least accurate.

Step 9: Consolidate Working Lists (Automated)

Once verification was complete, the working lists were consolidated into included, excluded and undetermined (Single Practitioner) lists. The included list contained address information for all identified and probable primary care clinics, as well as all additional address data (Type 1).

Step 10: Adjust Processing Steps Based on Learnings (Manual)

From the verification process, inaccurate inclusions and exclusions were noted and assessed to determine possible changes to the processing step (eg, addition of a key term) for automatic inclusion or exclusion in the future, thereby increasing accuracy of the algorithm.

This is an Appendix to a full manuscript published in the J Med Internet Res. For full copyright and citation information see http://dx.doi.org/10.2196/34141
