# Supplementary material for: Finding Primary Care—Repurposing Physician Registration Data to Generate a Regionally Accurate List of Primary Care Clinics: Development and Validation of an Open-Source Algorithm
Source: JMIR Form Res. 2022 Jun 22;6(6):e34141. doi: 10.2196/34141 (PMC9496812; doi:10.2196/34141)
Supplement: Multimedia Appendix 2 [file formative_v6i6e34141_app2.pdf]

<table>
<tr><th></th><th>Inclusion/Exclusion Criteria</th><th>RegEx Statement[1]</th></tr>
<tr><td>Hospital</td><td>Exclusion</td><td>((\b|^)(HOSP.*?)(\b|$))</td></tr>
<tr><td>Walk-in Clinic</td><td>Inclusion</td><td></td></tr>
<tr><td>Urgent and Primary Care</td><td>Inclusion</td><td></td></tr>
<tr><td>Long-Term Care</td><td>Exclusion</td><td>((\b|^)(LODGING|LODGE.*?|MANOR|SENIOR.*?|ALC)(\b|$))</td></tr>
<tr><td>Family</td><td>Inclusion</td><td>((\b|^)(FAMILY)\b\s*?(MED.*?|CLINIC|CENTRE|CENTER|ASSOCIATE.*?|CARE|PRACTICE)(\ b|$))</td></tr>
<tr><td>Corrections</td><td>Exclusion</td><td>((\b|^)(CORRECTION.*?|IMMIGRA.*?|PRETRIAL|CUSTODY|INSTITUTION.*?|DETENTION|HOLDING|HEALING\s*?VILLAGE)(\b|$))</td></tr>
<tr><td>First Nations</td><td>Inclusion</td><td>((\b|^)(FIRST\s*?NATION.*?|FIRST\s*?PEOPLE.*?|INDIGENOUS|NATIVE|ABORIGINAL|{Clinic Specific Name})(\b|$))</td></tr>
<tr><td>Sexual Health</td><td>Exclusion</td><td>((\b|^)(SEXUAL|STI|STD|{Clinic Specific Name})(\b|$))</td></tr>
<tr><td>Women’s Health</td><td>Exclusion</td><td>((\b|^)(WOM[AE]N.*?|MENOPAUSE|MATERN.*?|BIRTH.*?|OBSTETRIC.*?|GYNE.*?)(\b|$))</td></tr>
<tr><td>Virtual</td><td>Exclusion</td><td>((\b|^)({Organization Specific Name}|VIRTUAL|E-*?HEALTH|TELE.*?|I-*?HEALTH.*?)(\b|$))</td></tr>
<tr><td>Administrative</td><td>Exclusion</td><td>((\b|^)(AIRPORT|CONSULTING|ADMIN.*?|FRASER\sHEALTH\sAUTHORITY |FIRST\sNATION.*?\sHEALTH\sAUTHORITY|CORONER|CPSBC|COLLEGE\sOF\sPHYSICIAN.*?\sAND\sSURGEON.*?|HEALTH\s*?CANADA|VCH|WORKSAFE|WORKSAFEBC|WORKER.*?\s*?COMP.*?|BCAA|VETERAN.*?\s*?AFFAIR.*?|RCMP|AIR\s*?CANADA|QUALITY)(\b|$))</td></tr>
<tr><td>Clinic or Centre</td><td>Inclusion</td><td>((\b|^)(CLINIC.*?|ASSOCIATE.*?)(\b|$)) <i>or</i>
((\b|^)(CENTER|CENTRE|PRACTICE|DOCTOR.*?S)(\b|$))</td></tr>
</table>

[1]RegEx statements based on the search terms identified. {} brackets identify a search term specific to a clinic that is not a generic term and is not included in this list.

This is an Appendix to a full manuscript published in the J Med Internet Res. For full copyright and citation information see
http://dx.doi.org/10.2196/34141
