# Supplementary material for: Finding Primary Care—Repurposing Physician Registration Data to Generate a Regionally Accurate List of Primary Care Clinics: Development and Validation of an Open-Source Algorithm
Source: JMIR Form Res. 2022 Jun 22;6(6):e34141. doi: 10.2196/34141 (PMC9496812; doi:10.2196/34141)
Supplement: Multimedia Appendix 3 [file formative_v6i6e34141_app3.pdf]

Working Lists[1]	Included[2]/ Excluded[3]/ Undetermined [4]	Number of unique addresses[5]	Number of family physicians (FPs)[6] associated with addresses	Sample size used for verification[7] n= (% of total)	Number of addresses verified as currently providing primary care	Number of addresses verified as not currently providing primary care	Accuracy of filter to include or exclude, as predicted
Hospital	Excluded	182	1288	n/a[8]	n/a	182[8]	n/a
Walk-in Clinic	Included	220	932	n/a[8]	220[8]	n/a	n/a
Urgent and Primary Care Centre	Included	7	38	n/a[8]	(7)	n/a	n/a
Long-Term Care	Excluded	30	43	n/a[8]	n/a	30[8]	n/a
Family	Included	77	348	21 (27.3%)	20	1	95.2%
Corrections	Excluded	10	13	10 (100%)	0	10	100%
First Nations	Included	5	12	5 (100%)	4	1	80.0%
Sexual Health	Excluded	5	11	4 (100%)	0	4	100%
Women's Health	Excluded	11	24	11 (100%)	0	11	100%
Virtual	Excluded	7	20	7 (100%)	1	6	85.6%
Administrative	Excluded	28	66	23 (100%)	0	28	100%
Specialty	Excluded	83	159	83 (100%)	5	78	94.0%
Clinic or Centre	Included	563	1834	562 (99.8%)	493	69*	87.7%
Multi Practitioner	Included	367	1098	364 (99.2%)	292	54*	80.2%
Single Practitioner	Undetermined	1320	1320	211 (16.0%)	118	93	55.9%[9]

Appendix 3: Accuracy of algorithm sorting of individual physician addresses from licence registration to identify physical locations of primary care clinics

[1] Working lists created in Step 7 of the CLA
[2] Included = included as a probable location of a primary care clinic
[3] Excluded = excluded as a location that provides other non-primary care services
[4] Undetermined = unable to make assumptions based on the present algorithm, requires additional steps (i.e., individual verification) to exclude or include
[5] Unique addresses identified with FPs from the College of Physicians and Surgeons of British Columbia Registry List
[6] Registered as FPs with the College of Physicians and Surgeons of British Columbia
[7] Verification process for the presence of primary care services at an address using a professional informant, as well as internet and phone verification
[8] Addresses on these lists are verified externally and are therefore assumed to be correct
[9] Verification results from this working list were nonspecific, therefore the remaining 1109 single practitioner addresses require individual verification to determine if they are a location of a primary care clinic
*Contained clinics that were permanently closed and that were therefore excluded as not currently performing primary care

This is an Appendix to a full manuscript published in the J Med Internet Res. For full copyright and citation information see http://dx.doi.org/10.2196/34141
