# Supplementary material for: Finding Primary Care—Repurposing Physician Registration Data to Generate a Regionally Accurate List of Primary Care Clinics: Development and Validation of an Open-Source Algorithm
Source: JMIR Form Res. 2022 Jun 22;6(6):e34141. doi: 10.2196/34141 (PMC9496812; doi:10.2196/34141)
Supplement: Multimedia Appendix 4 [file formative_v6i6e34141_app4.pdf]

Internet validation process and phone call script

1) internet search of the clinic name, if available;
2) internet search of the exact address, unit number inclusive;
3) internet search of the street address only, no unit number, to identify residential buildings;
4) internet search of the FPs name, to attempt to identify where they work and if that matches the address on the CPSBC list;
5) phone call to the number provided on the CPSBC List registration (script below).
    a. [Introduction], my name is [insert name]. I work at UBC as a Research Assistant and I'm calling to learn a little bit about your clinic.
    b. Is this a family medicine clinic? [wait for response – if question see c) below] Are the services covered by BC Medical Services Plan? [wait for response] Are there any restrictions to who can be seen? [wait for response]
    c. [Only if questions about "family medicine"] Describe it as patients who see a family doctor for general health issues.
    d. Ask if they have a clinic website.

This is an Appendix to a full manuscript published in the J Med Internet Res. For full copyright and citation information see http://dx.doi.org/10.2196/34141
