# Supplementary material for: Finding Primary Care—Repurposing Physician Registration Data to Generate a Regionally Accurate List of Primary Care Clinics: Development and Validation of an Open-Source Algorithm
Source: JMIR Form Res. 2022 Jun 22;6(6):e34141. doi: 10.2196/34141 (PMC9496812; doi:10.2196/34141)
Supplement: Multimedia Appendix 5 [file formative_v6i6e34141_app5.pdf]

ID	PO_BOX	ADDRESS_FULL	CITY	PROVINCE	POSTAL_CODE	COUNTRY	GEO_LATITUDE	GEO_LONGITUDE	HA_ID	HA_NAME	NUM_PHYSICIANS
FAM_0001		BURNSIDE FAMILY MEDICAL CLINIC, 2, 101 BURNSIDE RD W	VICTORIA	BC	V9A 1B7	CANADA	48.4468486	-123.3794777	4 VIHA	Vancouver Island	4
FAM_0002		101 BURNSIDE RD W, SUITE 4	VICTORIA	BC	V9A 1B7	CANADA	48.4468486	-123.3794777	4 VIHA	Vancouver Island	5
FAM_0003		102, 10183 152A ST	SURREY	BC	V3R 4H6	CANADA	49.1875031	-122.7997353	2 FHA	Fraser	3
FAM_0004		SUITE 302, 1101 DEWDNEY AVE	TRAIL	BC	V1R 4T1	CANADA	49.0975305	-117.7087461	1 IHA	Interior	3
FAM_0005		304, 1101 DEWDNEY AVE	TRAIL	BC	V1R 4T1	CANADA	49.0975305	-117.7087461	1 IHA	Interior	8
FAM_0006	PO BOX 10	LADYSMITH FAMILY PRACTICE CLINIC, 1111 4 AVE	LADYSMITH	BC	V9G 1A1	CANADA	48.9958106	-123.8275049	4 VIHA	Vancouver Island	5
FAM_0007		BURRARD FAMILY MEDICINE, 500, 1144 BURRARD ST	VANCOUVER	BC	V6Z 2A5	CANADA	49.2793415	-123.1281537	3 VCHA	Vancouver Coastal	1
FAM_0008		202, 1193 KINGSWAY	VANCOUVER	BC	V5V 3C9	CANADA	49.2526105	-123.0802672	3 VCHA	Vancouver Coastal	16
FAM_0009		DRTIKHONOVA FAMILY MEDICAL CLINIC, 208, 1200 LYNN VALLEY RD	NORTH VANCOUVER	BC	V7J 2A2	CANADA	49.3360334	-123.0408014	3 VCHA	Vancouver Coastal	1
FAM_0010		JGM FAMILY PRACTICE CLINIC SUITE 1, 1230 ALWARD ST	PRINCE GEORGE	BC	V2M 7B1	CANADA	53.912914	-122.7661766	5 NHA	Northern	2
FAM_0011		1, 1230 ALWARD ST	PRINCE GEORGE	BC	V2M 7B1	CANADA	53.912914	-122.7661766	5 NHA	Northern	3
FAM_0012		FAMILY MEDICINE, CAPILANO MEDICAL CLINIC, 125 2 ST W, UNIT 101	NORTH VANCOUVER	BC	V7M 1C5	CANADA	49.3128439	-123.0789887	3 VCHA	Vancouver Coastal	2
FAM_0013		255, 1311 2 ST N	CRANBROOK	BC	V1C 3A1	CANADA	49.5128884	-115.7637975	1 IHA	Interior	8
FAM_0014		GEORGE ST FAMILY PRACTICE, 1, 1400 GEORGE ST	WHITE ROCK	BC	V4B 4A3	CANADA	49.0278028	-122.7993486	2 FHA	Fraser	7
FAM_0015		CENTRAL OKANAGAN DIVISION OF FAMILY PRACTICE, 201, 1405 ST PAUL ST	KELOWNA	BC	V1Y 2E4	CANADA	49.8886949	-119.4914197	1 IHA	Interior	1
FAM_0016		100, 145 15 ST W	NORTH VANCOUVER	BC	V7M 1R9	CANADA	49.3217918	-123.0744768	3 VCHA	Vancouver Coastal	3
FAM_0017		102, 1450 WADDINGTON RD	NANAIMO	BC	V9S 4V9	CANADA	49.1851172	-123.9624424	4 VIHA	Vancouver Island	5
FAM_0018		HEALTH WISE MEDICAL CLINIC, SUITE 3, 1499 GORDON DR	KELOWNA	BC	V1Y 9R2	CANADA	49.8861966	-119.4769375	1 IHA	Interior	3
FAM_0019		1668 TAMARACK ST	PRINCE GEORGE	BC	V2L 2T3	CANADA	53.9085588	-122.7589858	5 NHA	Northern	5
FAM_0020		MEDI, KEL FAMILY PRACTICE & WALK IN CLINIC, 324, 101, 1865 DILWORTH DR	KELOWNA	BC	V1Y 9T1	CANADA	49.8802564	-119.4365724	1 IHA	Interior	2
FAM_0021		(MILL CREEK FAMILY MEDICINE OFFICE FOR MATERNITY CARE PRACTICE), 1912 ENTERPRISE WAY UNIT 101	KELOWNA	BC	V1Y 9S9	CANADA	49.8846031	-119.453776	1 IHA	Interior	7
FAM_0022		107, C 1966 GUTHRIE RD	COMOX	BC	V9M 0B	CANADA	49.6890343	-124.933328			9
FAM_0023		114D, 20159 88 AVE	LANGLEY	BC	V1M 0A4	CANADA	49.1649752	-122.6646057	2 FHA	Fraser	2
FAM_0024		GOLDEN EARS FAMILY PRACTICE, 140, 20395 LOUGHEED HWY	MAPLE RIDGE	BC	V2X 2P9	CANADA	49.2196856	-122.6557605	2 FHA	Fraser	4
FAM_0025		NEWPORT FAMILY PRACTICE, 310, 205 NEWPORT DR	PORT MOODY	BC	V3H 5C9	CANADA	49.2828136	-122.827467	2 FHA	Fraser	2
FAM_0026		MAGETA MEDICAL CLINIC, 110, 20528 LOUGHEED HWY	MAPLE RIDGE	BC	V2X 2P8	CANADA	49.2181348	-122.652248	2 FHA	Fraser	8
FAM_0027		PORT AUGUSTA FAMILY MEDICINE CLINIC, UNIT 5,&6 COMOX CENTER MALL, 215 PORT AUGUSTA ST	COMOX	BC	V9M 3M9	CANADA	49.6737576	-124.9239317	4 VIHA	Vancouver Island	1
FAM_0028		PORT AUGSTUA FAMILY PRACTICE, UNITS 5&6, 215 PORT AUGUSTA ST	COMOX	BC	V9M 3M9	CANADA	49.6737576	-124.9239317	4 VIHA	Vancouver Island	3
FAM_0029		WILSON CENTRE FAMILY PRACTICE, 1, 2185 WILSON AVE	PORT COQUITLAM	BC	V3C 6C1	CANADA	49.2611675	-122.7770801	2 FHA	Fraser	13
FAM_0030		MURRAYVILLE FAMILY PRACTICE GROUP, 204, 22112 52 AVE	LANGLEY	BC	V2Y 2M6	CANADA	49.0963994	-122.6114208	2 FHA	Fraser	5
FAM_0031	PO BOX 2010	223 16 AVE N	CRESTON	BC	V0B 1G0	CANADA	49.0971564	-116.5071149	1 IHA	Interior	8
FAM_0032		UBC FACULTY OF MEDICINE, FAMILY PRACTICE RESIDENCY PROGRAM, CLINICAL ACADEMIC CAMPUS, 1 FLOOR, 2312 PANDOSY	KELOWNA	BC	V1Y 1T3	CANADA	49.8725291	-119.4919932	1 IHA	Interior	1
FAM_0033	PO BOX 99	FORT FAMILY PRACTICE, 23148 96 AVE	LANGLEY	BC	V1M 2R4	CANADA	49.1687943	-122.5818142	2 FHA	Fraser	5
FAM_0034		GUISACHAN MEDICAL CLINIC, 105, 2395 GORDON DR	KELOWNA	BC	V1W 3X7	CANADA	49.8712313	-119.4746348	1 IHA	Interior	11
FAM_0035		BAYSWATER FAMILY PRACTICE, 200, 2475 BAYSWATER ST	VANCOUVER	BC	V6K 4N3	CANADA	49.2644023	-123.1716107	3 VCHA	Vancouver Coastal	7
FAM_0036		2480 HEATHER ST	VANCOUVER	BC	V5Z 3H9	CANADA	49.2635856	-123.1196225	3 VCHA	Vancouver Coastal	12
FAM_0037		258 EAGLE RIDGE DR, EAGLE RIDGE FAMILY PRACTICE	SALT SPRING ISLAND	BC	V8K 2L1	CANADA	48.8644606	-123.4850647	4 VIHA	Vancouver Island	3
FAM_0038	PO BOX 240	SHAWNIGAN VILLAGE FAMILY PRACTICE, 2749 DUNDAS RD	SHAWNIGAN LAKE	BC	V0R 2W0	CANADA	48.6533854	-123.6215257	4 VIHA	Vancouver Island	6
FAM_0039		2778 104 ST	NANAIMO	BC	V9T 2E4	CANADA	49.2069135	-124.013865	4 VIHA	Vancouver Island	7
FAM_0040		3029 CAMBIE ST	VANCOUVER	BC	V5Z 4N2	CANADA	49.2578453	-123.1158169	3 VCHA	Vancouver Coastal	5
FAM_0041		FAMILY PRACTICE, 403B, 3210 25 AVE	VERNON	BC	V1T 1P1	CANADA	50.2603819	-119.2749639	1 IHA	Interior	4
FAM_0042		VERNON FAMILY PRACTICE ASSOCIATES, 200 3310 32 AVE	VERNON	BC	V1T 2M6	CANADA	50.2652047	-119.2764732	1 IHA	Interior	5
FAM_0043		350 FESTUBERT ST	DUNCAN	BC	V9L 3S9	CANADA	48.7779221	-123.7016569	4 VIHA	Vancouver Island	3
FAM_0044	PO BOX 102	3731 1 AVE	SMITHERS	BC	V0J 2N0	CANADA	54.7784887	-127.1709479	5 NHA	Northern	2
FAM_0045		SHAUGHNESSY FAMILY MEDICINE, 3770 OAK ST	VANCOUVER	BC	V6H 2M3	CANADA	49.2519744	-123.1270319	3 VCHA	Vancouver Coastal	4
FAM_0046		UNIVERSITY PLAZA FAMILY PRACTICE, 200, 3994 SHELBOURNE ST	VICTORIA	BC	V8N 3E2	CANADA	48.4719624	-123.3334163	4 VIHA	Vancouver Island	4
FAM_0047		UPSTREAM FAMILY MEDICINE, 108, 4330 HIGHWAY 101	SECHELT	BC	V0N 3A1	CANADA	49.4403593	-123.7090231	3 VCHA	Vancouver Coastal	5
FAM_0048		VIEWMONT FAMILY MEDICINE, 210, 4475 VIEWMONT AVE	VICTORIA	BC	V8Z 6L8	CANADA	48.4931896	-123.3892105	4 VIHA	Vancouver Island	3
FAM_0049		DEPT OF FAMILY PRACTICE, 4490 OAK ST	VANCOUVER	BC	V6H 3N1	CANADA	49.2447107	-123.1275681	3 VCHA	Vancouver Coastal	1
FAM_0050		LADNER FAMILY PRACTICE CLINIC, 204, 4515 HARVEST DR	LADNER	BC	V4K 4L1	CANADA	49.0850087	-123.0647032	2 FHA	Fraser	4
FAM_0051		SARDIS FAMILY PRACTICE, 202, 45425 LUCKAKUCK WAY	CHILLIWACK	BC	V2R 2T7	CANADA	49.1418723	-121.9672203	2 FHA	Fraser	3
FAM_0052		FAMILY CARE CENTRE, 45779 LUCKAKUCK WAY	CHILLIWACK	BC	V2R 4E8	CANADA	49.1425248	-121.9591416	2 FHA	Fraser	2
FAM_0053		CROSSROADS FAMILY PRACTICE, 201, 45800 PROMONTORY RD	CHILLIWACK	BC	V2R 5Z5	CANADA	49.110887	-121.9570107	2 FHA	Fraser	9
FAM_0054		FIFTH ST FAMILY PRACTICE, 519 5 ST	COURTENAY	BC	V9N 1K2	CANADA	49.6898847	-125.0022515	4 VIHA	Vancouver Island	1
FAM_0055		FIFTH ST FAMILY PRACTICE, 519G 5 ST	COURTENAY	BC	V9N 1K2	CANADA	49.6897758	-125.0021568	4 VIHA	Vancouver Island	3
FAM_0056		101, 5625 PROMONTORY RD, ZENITH FAMILY PRACTICE	CHILLIWACK	BC	V2R 4M5	CANADA	49.1044338	-121.9358127	2 FHA	Fraser	1
FAM_0057		SHUSWAP FAMILY PRACTICE, 102, 571 6 ST NE	SALMON ARM	BC	V1E 1R6	CANADA	50.7039752	-119.2792178	1 IHA	Interior	4
FAM_0058		ST LUKE FAMILY PRACTICE, 5761 GLOVER RD	LANGLEY	BC	V3A 8M8	CANADA	49.1087532	-122.6507475	2 FHA	Fraser	1
FAM_0059		ST LUKE FAMILY PRACTICE, 5, 5761 GLOVER RD	LANGLEY	BC	V3A 8M8	CANADA	49.1087532	-122.6507475	2 FHA	Fraser	3
FAM_0060		FAMILY PRACTICE, DAVID STRANGWAY BLDG, UNDERGRADUATE PROGRAMS DIRECTOR, 300, 5950 UNIVERSITY BLVD	VANCOUVER	BC	V6T 1Z3	CANADA	49.2658095	-123.2462171	3 VCHA	Vancouver Coastal	1
FAM_0061		UBC HEALTH CLINIC, 310, 5950 UNIVERSITY BLVD	VANCOUVER	BC	V6T 1Z3	CANADA	49.2658095	-123.2462171	3 VCHA	Vancouver Coastal	6
FAM_0062		U6, 601 W BROADWAY	VANCOUVER	BC	V5Z 4C2	CANADA	49.2632736	-123.1177186	3 VCHA	Vancouver Coastal	5
FAM_0063		242, 610 SIXTH ST	NEW WESTMINSTER	BC	V3L 3C2	CANADA	49.2134839	-122.9217729	2 FHA	Fraser	6
FAM_0064		SUITE 200, 6411 NELSON AVE	BURNABY	BC	V5H 4H3	CANADA	49.2255332	-122.99472	2 FHA	Fraser	4
FAM_0065		6422 BAY ST	WEST VANCOUVER	BC	V7W 2H1	CANADA	49.3745863	-123.2751322	3 VCHA	Vancouver Coastal	7
FAM_0066		WEST COAST FAMILY MEDICAL CLINIC, 1300, 6660 SOOKE RD	SOOKE	BC	V9Z 0A5	CANADA	48.3797908	-123.721312	4 VIHA	Vancouver Island	10
FAM_0067		OAK TREE FAMILY MEDICINE, 6681 VICTORIA DR	VANCOUVER	BC	V5P 3Y2	CANADA	49.2236369	-123.0661803	3 VCHA	Vancouver Coastal	2
FAM_0068		102B, 6844 KING GEORGE BLVD	SURREY	BC	V3W 4Z9	CANADA	49.1270342	-122.8455285	2 FHA	Fraser	2
FAM_0069		SCOTT RD FAMILY CARE MEDICAL/REHAB CLINIC, 103, 7110 120 ST	SURREY	BC	V3W 3M8	CANADA	49.1322689	-122.8900721	2 FHA	Fraser	2
FAM_0070		MISSION MATERNITY CLINIC, DIVISION OF FAMILY PRACTICE, 7298 HURD ST	MISSION	BC	V2V 3H6	CANADA	49.1348623	-122.3319461	2 FHA	Fraser	8
FAM_0071		VANCOUVER FAMILY PRACTICE CENTRE, 402, 750 BROADWAY W	VANCOUVER	BC	V5Z 1H3	CANADA	49.2630118	-123.1210731	3 VCHA	Vancouver Coastal	5
FAM_0072		UBC FAMILY PRACTICE CENTRE, 400, 750 BROADWAY AVE W	VANCOUVER	BC	V5Z 1H3	CANADA	49.2630118	-123.1210731	3 VCHA	Vancouver Coastal	5
FAM_0073		8 AVE FAMILY AND MATERNITY CARE, 888 8 AVE W	VANCOUVER	BC	V5Z 3Y1	CANADA	49.2640136	-123.1235113	3 VCHA	Vancouver Coastal	1
FAM_0074		304, 888 8 AVE W	VANCOUVER	BC	V5Z 3Y1	CANADA	49.2640136	-123.1235113	3 VCHA	Vancouver Coastal	10
FAM_0075		FAMILY CARE MEDICAL CLINIC, 190, 9040 BLUNDELL RD	RICHMOND	BC	V6Y 1K3	CANADA	49.155408	-123.1235036	3 VCHA	Vancouver Coastal	3
FAM_0076		FAMILY PRACTICE/ EMERGENCY, 4, 910 COLUMBIA ST WEST	KAMLOOPS	BC	V2C 1L2	CANADA	50.6683894	-120.3551873	1 IHA	Interior	2
FAM_0077	BOX 868	ASHCROFT FAMILY MEDICAL PRACTICE	ASHCROFT	BC	V0K 1A0	CANADA			1 IHA	Interior	1
FN_0001		FIRST NATIONS HEALTH AUTHORITY, 501, 100 PARK ROYAL S	WEST VANCOUVER	BC	V7T 1A2	CANADA	49.3252402	-123.1347282	3 VCHA	Vancouver Coastal	1
FN_0002		URBAN ABORIGINAL HEALTH CENTRE, 119, 125 PALM ST	KAMLOOPS	BC	V2B 8J7	CANADA	50.6855733	-120.3564179	1 IHA	Interior	2
FN_0003		VANCOUVER NATIVE HEALTH, 449 HASTINGS ST E	VANCOUVER	BC	V6A 1P5	CANADA	49.2815413	-123.0942367	3 VCHA	Vancouver Coastal	4
FN_0004		KILALA LELUM HEALTH CENTRE, 626 POWELL ST	VANCOUVER	BC	V6A 1H4	CANADA	49.2829938	-123.0907991	3 VCHA	Vancouver Coastal	4
FN_0005	PO BOX 765	SEABIRD ISLAND FIRST NATION HEALTH	AGASSIZ	BC	V0M 1A0	CANADA			2 FHA	Fraser	1
PCC_0001		MCLEOD MEDICAL CLINIC, 229, 100 TABOR BLVD	PRINCE GEORGE	BC	V2M 5T4	CANADA	53.9265841	-122.8028377	5 NHA	Northern	1
PCC_0002		100 WALLACE ST SUITE 1	NANAIMO	BC	V9R 5B1	CANADA	49.1683508	-123.9416342	4 VIHA	Vancouver Island	7
PCC_0003		THE WELL MEDICAL CLINIC, E, 1001 AUSTIN AV	COQUITLAM	BC	V3K 3N9	CANADA	49.2493565	-122.8676106	2 FHA	Fraser	2
PCC_0004		140, 1005 COLUMBIA ST	NEW WESTMINSTER	BC	V3M 6H5	CANADA	49.2004903	-122.9160205	2 FHA	Fraser	2
PCC_0005		CASTLEGAR MEDICAL ASSOCIATES, 1008 COLUMBIA AVE	CASTLEGAR	BC	V1N 1H2	CANADA	49.3140601	-117.6636088	1 IHA	Interior	7
PCC_0006		ALLIANCE CLINIC, 10085 WHALLEY BLVD	SURREY	BC	V3T 4G1	CANADA	49.185684	-122.8434265	2 FHA	Fraser	3
PCC_0007		LARGE CLINIC ATTN: DR CRAIG LYNCH, 1, 101 1 AVE E	PRINCE RUPERT	BC	V8J 3X4	CANADA	54.3166864	-130.3231555	5 NHA	Northern	1
PCC_0008		THE LARGE CLINIC, 100, 101 1 AVE E	PRINCE RUPERT	BC	V8J 3X4	CANADA	54.3166864	-130.3231555	5 NHA	Northern	5
PCC_0009	PO BOX 590	SELKIRK MEDICAL CLINIC, 101 1 W ST	REVELSTOKE	BC	V0E 2S0	CANADA	50.9988813	-118.1957433	1 IHA	Interior	5
PCC_0010	PO BOX 3110	3110 CENTRAL CITY, 10153 KING GEORGE HWY	SURREY	BC	V3T 2W1	CANADA	49.1855931	-122.8473969	2 FHA	Fraser	4
PCC_0011		VERALIFE HEALTH CENTRE, 103, 8386120 ST	SURREY	BC	V3W 3N4	CANADA	49.1898197	-122.8117594	2 FHA	Fraser	1
PCC_0012		MIND LONGEVITY CENTER, INC, 125A, 1030 DENMAN ST	VANCOUVER	BC	V6G 2M6	CANADA	49.2889238	-123.1392459	3 VCHA	Vancouver Coastal	1
PCC_0013	PO BOX 599	HUDSON'S HOPE HEALTH CENTRE, 10309 KYLLO ST	HUDSON'S HOPE	BC	V0C 1V0	CANADA	56.0263657	-121.9157147	5 NHA	Northern	1
PCC_0014		LOYAL MEDICAL CLINIC, 112, 1055	VANCOUVER	BC	V6E 3P1	CANADA	49.1813757	-123.9471511	3 VCHA	Vancouver Coastal	1
PCC_0015		1061 HAMILTON ST	VANCOUVER	BC	V6B 5T4	CANADA	49.2763482	-123.1204839	3 VCHA	Vancouver Coastal	3
PCC_0016		MARINE DR MEDICAL CLINIC, 102, 1061 MARINE DR	NORTH VANCOUVER	BC	V7P 1S6	CANADA	49.3235475	-123.1035419	3 VCHA	Vancouver Coastal	1
PCC_0017		102, 10663 KING GEORGE BLVD	SURREY	BC	V3T 2X6	CANADA	49.1965164	-122.8456678	2 FHA	Fraser	4
PCC_0018		KEYS CLINIC, ST HEALTH OUTREACH PROGRAM, 10667 135A ST	SURREY	BC	V3T 4E3	CANADA	49.1963771	-122.847063	2 FHA	Fraser	1
PCC_0019		107 8 AVE, LILLOOET MEDICAL CLINIC	LILLOOET	BC	V0K 1V0	CANADA	50.691343	-121.9385317	1 IHA	Interior	4
PCC_0020		GATEWAY MEDICAL CENTRE, 10751 KING GEORGE BOUL	SURREY	BC	V3T 2X6	CANADA	49.1978627	-122.8457959	2 FHA	Fraser	1
PCC_0021		TSAWWASSEN MEDICAL CLINIC, 1077 56 ST	DELTA	BC	V4L 2A2	CANADA	49.0228099	-123.0686993	2 FHA	Fraser	1

**PCC_0022**		PORT ALICE HEALTH CENTRE, 1090 MARINE DR	PORT ALICE	BC	V0N 2N0	CANADA	50.4256586	-127.4883092	4 VIHA	Vancouver Island	1
**PCC_0023**		DOCTOR, SCOTTSDALE MEDICAL CENTER, 110, 7928, 128ST, YORK BUSINESS CENTER	SURREY	BC	V3W 4E8	CANADA	49.2024433	-122.8221601	2 FHA	Fraser	1
**PCC_0024**		102, 1100 ALASKA AVE	DAWSON CREEK	BC	V1G 4V8	CANADA	55.7604489	-120.2310667	5 NHA	Northern	2
**PCC_0025**		101, 1100 ALASKA AVE	DAWSON CREEK	BC	V1G 4V8	CANADA	55.7604181	-120.2305905	5 NHA	Northern	4
**PCC_0026**		1101 3 ST	CASTLEGAR	BC	V1N 2A1	CANADA	49.3265946	-117.6645688	1 IHA	Interior	6
**PCC_0027**		111, MOUNTAIN VIEW MEDICAL CLINIC, DOGWOOD ST	CAMPBELL RIVER	BC	V9H 6B9	CANADA	50.0095206	-125.2470366			3
**PCC_0028**		BASTION MEDICAL CENTRE	SALMON ARM	BC	V1E 2G1	CANADA	50.707812	-119.2555288	1 IHA	Interior	1
**PCC_0029**		HARBOUR CITY HEALTH CENTRE, 204, 1125 DUFFERIN CRES	NANAIMO	BC	V9S 2B5	CANADA	49.1840471	-123.9672873	4 VIHA	Vancouver Island	1
**PCC_0030**		1128 HORNBY ST, 4 FLOOR	VANCOUVER	BC	V6Z 2L4	CANADA	49.278937	-123.126832	3 VCHA	Vancouver Coastal	2
**PCC_0031**		THREE BRIDGES COMMUNITY HEALTH CENTER, 1128 HORNBY ST	VANCOUVER	BC	V6Z 2L4	CANADA	49.278937	-123.126832	3 VCHA	Vancouver Coastal	6
**PCC_0032**		FW GREEN CLINIC, 262, 1131, 2 ST N	CRANBROOK	BC	V1C 3L1	CANADA	49.508454	-115.7657633	1 IHA	Interior	1
**PCC_0033**		WESTSIDE MEDICAL, 103, 1135 STEVENS RD	WEST KELOWNA	BC	V1Z 2S8	CANADA	49.8660094	-119.5634336	1 IHA	Interior	5
**PCC_0034**		ESQUIMALT MEDICAL CLINIC, 1153 ESQUIMALT RD 3	VICTORIA	BC	V9A 3N7	CANADA	48.4294672	-123.4107153	4 VIHA	Vancouver Island	2
**PCC_0035**		RODRIGUEZ MEDICAL CLINIC, 104, 1168 THE HIGH ST	COQUITLAM	BC	V3B 0C6	CANADA	49.2821917	-122.7969526	2 FHA	Fraser	3
**PCC_0036**		MAPLE TREE MEDICAL CLINIC, 1181 6 AVE NE	SALMON ARM	BC	V1E 2S5	CANADA	50.7044737	-119.2714868	1 IHA	Interior	7
**PCC_0037**		FRASER MEDICAL CLINIC, UNIT 100, 11915 224 ST	MAPLE RIDGE	BC	V2X 6B2	CANADA	49.2189274	-122.6021356	2 FHA	Fraser	1
**PCC_0038**		FRASER MEDICAL CLINIC, 101, 11915 224 ST	MAPLE RIDGE	BC	V2X 6B2	CANADA	49.2189274	-122.6021356	2 FHA	Fraser	1
**PCC_0039**		FRASER MEDICAL CLINIC, 11915 224 ST	MAPLE RIDGE	BC	V2X 6B2	CANADA	49.2189274	-122.6021356	2 FHA	Fraser	2
**PCC_0040**		1199 AUSTIN AVE	COQUITLAM	BC	V3K 3P4	CANADA	49.2492922	-122.8582747	2 FHA	Fraser	5
**PCC_0041**	PO BOX 3760	1209 MAIN ST	SMITHERS	BC	V0J 2N0	CANADA	54.7803513	-127.170402	5 NHA	Northern	4
**PCC_0042**		MILE 0 WALK IN CLINIC, 121, 10200, 8ST	DAWSON CREEK	BC	V1G 3P8	CANADA	55.7401395	-120.2288883	5 NHA	Northern	1
**PCC_0043**		PITT MEADOWS PROFESSIONAL CENTRE, 101, 12195 HARRIS RD	PITT MEADOWS	BC	V3Y 2E9	CANADA	49.2243315	-122.6900941	2 FHA	Fraser	2
**PCC_0044**		123 10 AVE S	CRANBROOK	BC	V1C 2N1	CANADA	49.5092582	-115.767983	1 IHA	Interior	9
**PCC_0045**	BOX 1700	BLUE HERON MEDICAL CLINIC, 203, 1230 CANYON ST	CRESTON	BC	V0B 1G0	CANADA	49.0956112	-116.509764	1 IHA	Interior	3
**PCC_0046**		HEALTHVUE MEDICAL CLINIC, 100, 12420 NO1 RD	RICHMOND	BC	V7E 6N2	CANADA	49.124758	-123.1808242	3 VCHA	Vancouver Coastal	5
**PCC_0047**		105, 1250 DOMINION AVE	PORT COQUITLAM	BC	V3B 8G7	CANADA	49.2580714	-122.7486628	2 FHA	Fraser	2
**PCC_0048**		1256 MARINE DR	NORTH VANCOUVER	BC	V7P 1T2	CANADA	49.3247652	-123.1093259	3 VCHA	Vancouver Coastal	6
**PCC_0049**		GRANVILLE YOUTH HEALTH CLINIC, 1260 GRANVILLE ST	VANCOUVER	BC	V6Z 1M4	CANADA	49.2763342	-123.1268421	3 VCHA	Vancouver Coastal	1
**PCC_0050**		ALL CARE MEDICAL CENTRE, 12827 76 AVE 101	SURREY	BC	V3W 2V3	CANADA	49.141173	-122.8671536	2 FHA	Fraser	1
**PCC_0051**		SITKA HEALTH CENTRE, 1284 FAIRFIELD RD	VICTORIA	BC	V8V 4W3	CANADA	48.4157697	-123.3491836	4 VIHA	Vancouver Island	4
**PCC_0052**		CEDAR HILLS MEDICAL CLINIC, 12852 96 AVE, UNIT 120	SURREY	BC	V3V 6A8	CANADA	49.1769549	-122.8656881	2 FHA	Fraser	2
**PCC_0053**		SUKHMAN MEDICAL CLINIC, 109, 12885 80TH AVE	SURREY	BC	V3W 0E6	CANADA	49.1502749	-122.8659648	2 FHA	Fraser	1
**PCC_0054**		SEYMOUR HEALTH CENTRE, 1290 HORNBY ST FLOOR 2	VANCOUVER	BC	V6Z 1W2	CANADA	49.2772743	-123.1293398	3 VCHA	Vancouver Coastal	1
**PCC_0055**		3 BRIDGES COMMUNITY HEALTH CENTRE, 1292 HORNBY ST	VANCOUVER	BC	V6Z 1W2	CANADA	49.2774005	-123.1295869	3 VCHA	Vancouver Coastal	2
**PCC_0056**	BOX 98	FRASER LAKE MEDICAL CLINIC, 130 CHOWSUNKET ST	FRASER LAKE	BC	V0J 1S0	CANADA	54.0591174	-124.8494433	5 NHA	Northern	6
**PCC_0057**	PO BOX 460	13009 ROSEDALE AVE	SUMMERLAND	BC	V0H 1Z0	CANADA	49.6012398	-119.6773328	1 IHA	Interior	6
**PCC_0058**		1302 7 AVE SUITE 102	PRINCE GEORGE	BC	V2L 3P1	CANADA	53.9144924	-122.7488382	5 NHA	Northern	3
**PCC_0059**		202, 1302 7 AVE	PRINCE GEORGE	BC	V2L 3P1	CANADA	53.9141274	-122.7486071	5 NHA	Northern	5
**PCC_0060**		1306 CENTRAL ST E	PRINCE GEORGE	BC	V2M 3C1	CANADA	53.9122586	-122.7808912	5 NHA	Northern	5
**PCC_0061**		FW GREEN CLINIC, 262, 1311 SECOND ST N	CRANBROOK	BC	V1C 3L1	CANADA	49.5128884	-115.7637975	1 IHA	Interior	10
**PCC_0062**		1315 ST GEORGES AVE	NORTH VANCOUVER	BC	V7L 3J2	CANADA	49.3202668	-123.0697176	3 VCHA	Vancouver Coastal	4
**PCC_0063**	PO BOX 10003	ELAHO MEDICAL CLINIC, 1337 PEMBERTON AVE	SQUAMISH	BC	V8B 0R1	CANADA	49.7016265	-123.1523587	3 VCHA	Vancouver Coastal	5
**PCC_0064**		1300, 13401, 108 AVE	SURREY	BC	V3T 5T3	CANADA	49.1990923	-122.8502541	2 FHA	Fraser	2
**PCC_0065**		13444 108 AVE, LIFE GATEWAY MEDICAL CLINIC	SURREY	BC	V3T 2K1	CANADA	49.1987563	-122.8495496	2 FHA	Fraser	1
**PCC_0066**		LIONS GATE MEDICAL CLINIC, 201, 135 15 ST E	NORTH VANCOUVER	BC	V7L 2P7	CANADA	49.3217471	-123.0708344	3 VCHA	Vancouver Coastal	2
**PCC_0067**		COURTENAY MEDICAL ASSOCIATES, 201, 1350 ENGLAND AVE	COURTENAY	BC	V9N 8X6	CANADA	49.6860441	-124.9944659	4 VIHA	Vancouver Island	6
**PCC_0068**		THE DE PIERI CLINIC, 106, 1353 ELLIS ST	KELOWNA	BC	V1Y 1Z9	CANADA	49.890011	-119.4930484	1 IHA	Interior	2
**PCC_0069**		THE PHOENIX CENTRE, 13686 94A AVE	SURREY	BC	V3V 1N1	CANADA	49.1745351	-122.843306	2 FHA	Fraser	1
**PCC_0070**		GATEWAY MEDICAL CENTER, 13719 72 AVE	SURREY	BC	V3W 2P2	CANADA	49.1337485	-122.8421959	2 FHA	Fraser	7
**PCC_0071**	BOX 781	COLUMBIA COAST CLINIC, 1371 B CEDAR ST, STN A	CAMPBELL RIVER	BC	V9W 6Y4	CANADA	50.0286822	-125.2472915	4 VIHA	Vancouver Island	1
**PCC_0072**		COPEMAN HEALTH CARE CENTRE, 220, 138 EAST 13 ST	NORTH VANCOUVER	BC	V7L 0E5	CANADA	49.3204014	-123.0710295	3 VCHA	Vancouver Coastal	8
**PCC_0073**		LAKESIDE MEDICAL CLINIC, 1219, 138 S SHORE RD	LAKE COWICHAN	BC	V0R 2G0	CANADA	48.823327	-124.0574911	4 VIHA	Vancouver Island	1
**PCC_0074**	PO BOX BOX 1	LAKESIDE MEDICAL CLINIC, 138 SOUTH SHORE RD	LAKE COWICHAN	BC	V0R 2G0	CANADA	48.823327	-124.0574911	4 VIHA	Vancouver Island	1
**PCC_0075**		AMARI MEDICAL CLINIC, 13805 104 AVE UNIT 102	SURREY	BC	V3T 1W7	CANADA	49.1919092	-122.8394092	2 FHA	Fraser	5
**PCC_0076**		VALLEYVIEW MEDICAL CLINIC, 27, 1400 COWICHAN BAY RD	COBBLE HILL	BC	V0R 1L0	CANADA	48.7101595	-123.6082151	4 VIHA	Vancouver Island	1
**PCC_0077**	PO BOX 69	PEMBERTON MEDICAL CENTRE, 1403 PORTAGE RD	PEMBERTON	BC	V0N 2L0	CANADA	50.3209128	-122.8047946	3 VCHA	Vancouver Coastal	3
**PCC_0078**		1405 SPRUCE ST	CAMPBELL RIVER	BC	V9W 7K1	CANADA	50.0292785	-125.27048	4 VIHA	Vancouver Island	2
**PCC_0079**		1424B ISLAND HWY, RIVER CITY MEDICAL CLINIC	CAMPBELL RIVER	BC	V9W 8C9	CANADA	50.0340816	-125.2509981	4 VIHA	Vancouver Island	1
**PCC_0080**		YORSTON MEDICAL CLINIC, 143 FOURTH AVE S	WILLIAMS LAKE	BC	V2G 1J8	CANADA	52.1263498	-122.1412404	1 IHA	Interior	2
**PCC_0081**		101, 143 FOURTH AVE S	WILLIAMS LAKE	BC	V2G 1J8	CANADA	52.1263498	-122.1412404	1 IHA	Interior	7
**PCC_0082**		106, 14340 64 AVE	SURREY	BC	V3W 1Z1	CANADA	49.1189167	-122.8250531	2 FHA	Fraser	3
**PCC_0083**		1443 VIEW CRES	DELTA	BC	V4L 2K2	CANADA	49.0292211	-123.0682007	2 FHA	Fraser	4
**PCC_0084**		1450 WADDINGTON RD	NANAIMO	BC	V9S 4V9	CANADA	49.1851172	-123.9624424	4 VIHA	Vancouver Island	6
**PCC_0085**		HEALTH POINT CARE CENTRE, 1454 HILLSIDE AVE	VICTORIA	BC	V8T 2B7	CANADA	48.4425235	-123.342568	4 VIHA	Vancouver Island	4
**PCC_0086**		210, 1465 SALISBURY AVE	PORT COQUITLAM	BC	V3B 6J3	CANADA	49.2728126	-122.7550096	2 FHA	Fraser	7
**PCC_0087**		HEALTHCONNECTION CLINIC, 148 15 ST E	NORTH VANCOUVER	BC	V7V 2Z3	CANADA	49.3223321	-123.0698208	3 VCHA	Vancouver Coastal	1
**PCC_0088**		FIRST CANADIAN MEDICAL CENTER, 14888 104 AVE UNIT 104	SURREY	BC	V3R 1M4	CANADA	49.1910464	-122.8098353	2 FHA	Fraser	4
**PCC_0089**		GROW CLINIC 205, 1497 ADMIRALS RD	VICTORIA	BC	V9A 7N8	CANADA	48.4475395	-123.4264501	4 VIHA	Vancouver Island	3
**PCC_0090**		CENTURIA MEDICAL, 2, 1499 GORDON DR	KELOWNA	BC	V1Y 9R2	CANADA	49.8861966	-119.4769375	1 IHA	Interior	3
**PCC_0091**	PO BOX 538	HOUSTON HEALTH CENTRE, 14 ST	HOUSTON	BC	V0J 1Z0	CANADA	54.3965027	-126.6464198	5 NHA	Northern	1
**PCC_0092**		100, 15129 100 AVE	SURREY	BC	V3R 9P4	CANADA	49.1844108	-122.8027054	2 FHA	Fraser	4
**PCC_0093**		FOCUSMED CLINIC, 200, 15135 101 AVE	SURREY	BC	V3R 7Z1	CANADA	49.1863027	-122.8026987	2 FHA	Fraser	3
**PCC_0094**		PANORAMA VILLAGE MEDICAL CLINIC, 103, 15157 56 AVE, HIGHWAY 10	SURREY	BC	V3S 9A5	CANADA	49.1053336	-122.8027414	2 FHA	Fraser	7
**PCC_0095**		101, 1516 FAIRVIEW RD	PENTICTON	BC	V2A 6A3	CANADA	49.4819532	-119.5930404	1 IHA	Interior	5
**PCC_0096**		15229 RUSSELL AVE	WHITE ROCK	BC	V4B 5C3	CANADA	49.0295802	-122.8000195	2 FHA	Fraser	3
**PCC_0097**		106, 15252 32 AVE	SURREY	BC	V3S 0R7	CANADA	49.0610132	-122.7984631	2 FHA	Fraser	9
**PCC_0098**		200, 1530 W7 AVE, SEYMOUR HEALTH CENTRE	VANCOUVER	BC	V6J 1S3	CANADA	49.2653242	-123.1391941	3 VCHA	Vancouver Coastal	6
**PCC_0099**		SULLIVAN MEDICAL CLINIC, 15325 56 AVE	SURREY	BC	V3S 0X9	CANADA	49.1052414	-122.7984342	2 FHA	Fraser	1
**PCC_0100**		HILLTOP MEDICAL CLINIC, 200, 15331 16 AVE	SURREY	BC	V4A 0C1	CANADA	49.0313142	-122.798629	2 FHA	Fraser	18
**PCC_0101**		HYGIEA MEDICAL CLINIC, 15336 31 AVE, SUITE 300	SURREY	BC	V3Z 0X2	CANADA	49.0579213	-122.7969245	2 FHA	Fraser	1
**PCC_0102**	PO BOX 203	15355 24 AVE 410	SURREY	BC	V4A 2H9	CANADA	49.0458355	-122.7975087	2 FHA	Fraser	2
**PCC_0103**		HIGHROADS MEDICAL CLINIC, 401, 15355 24 AVE	SURREY	BC	V4A 2H9	CANADA	49.0458355	-122.7975087	2 FHA	Fraser	2
**PCC_0104**		PENINSULA MEDICAL CLINIC, 109, 15388 24 AVE	SURREY	BC	V4A 2J2	CANADA	49.045415	-122.7964025	2 FHA	Fraser	3
**PCC_0105**	PO BOX 250	154 MEMORIAL AVE	PARKSVILLE	BC	V9P 2G4	CANADA	49.3196578	-124.3151253	4 VIHA	Vancouver Island	9
**PCC_0106**		WHITE ROCK MEDICAL ASSOCIATES, 15451 RUSSELL AVE	WHITE ROCK	BC	V4B 2R5	CANADA	49.029551	-122.7945119	2 FHA	Fraser	5
**PCC_0107**		101, 15451 RUSSELL AVE	WHITE ROCK	BC	V4B 2R5	CANADA	49.029551	-122.7945119	2 FHA	Fraser	10
**PCC_0108**		PRIMARY CARE ACCESS CLINIC, 15476 VINE AVE, BERKLEY PAVILLION	WHITE ROCK	BC	V4B 5M2	CANADA	49.0301548	-122.7935154	2 FHA	Fraser	1
**PCC_0109**		LOTUS INTEGRATIVE MEDICAL CENTRE, 15551 MARINE DR	WHITE ROCK	BC	V4B 1C9	CANADA	49.017241	-122.7915308	2 FHA	Fraser	1
**PCC_0110**		140, 1575 MCCALLUM RD	ABBOTSFORD	BC	V2S 0K2	CANADA	49.0307425	-122.2935838	2 FHA	Fraser	3
**PCC_0111**		SEAWALL MEDICAL CENTER, 102, 1590 BELLEVUE AVE	WEST VANCOUVER	BC	V7V 3R5	CANADA	49.3274895	-123.1583908	3 VCHA	Vancouver Coastal	1
**PCC_0112**		ROSS BAY HEALTH CENTRE, 16, 1594 FAIRFIELD RD	VICTORIA	BC	V8S 1G1	CANADA	48.4127737	-123.3383309	4 VIHA	Vancouver Island	4
**PCC_0113**		EASTSHORE HEALTH UNIT, 15985 HIGHWAY 3A	CRAWFORD BAY	BC	V0B 1E0	CANADA	49.6723897	-116.8246896	1 IHA	Interior	2
**PCC_0114**		UNIT 201, 16088 84 AVE	SURREY	BC	V4N 0V9	CANADA	49.1551695	-122.7763394	2 FHA	Fraser	5
**PCC_0115**		GRACEMED CLINIC, 102, 1629 TOWNSITE RD	NANAIMO	BC	V9S 1N3	CANADA	49.1812857	-123.9774969	4 VIHA	Vancouver Island	1
**PCC_0116**		ROYAL OAK CLINIC, 103, 1638 BROADWAY E	VANCOUVER	BC	V5N 1W1	CANADA	49.2621083	-123.0707674	3 VCHA	Vancouver Coastal	5
**PCC_0117**		ATLIN HEALTH CENTRE, 164 THIRD ST	ATLIN	BC	V0W 1A0	CANADA	59.5733591	-133.7014085	5 NHA	Northern	2
**PCC_0118**		164 KINGS LANE	SALT SPRING ISLAND	BC	V8K 2P7	CANADA	48.8638825	-123.5110295	4 VIHA	Vancouver Island	6
**PCC_0119**		OAK BAY MEDICAL CLINIC, 1640 OAK BAY AVE	VICTORIA	BC	V8R 1B2	CANADA	48.4267034	-123.334592	4 VIHA	Vancouver Island	3
**PCC_0120**		130, 1641 HILLSIDE AVE	VICTORIA	BC	V8T 5G1	CANADA	48.4450083	-123.3337894	4 VIHA	Vancouver Island	3
**PCC_0121**	BOX 48	WEST CHILCOTIN HEALTH CENTRE, 16452 HIGHWAY 20	TATLA LAKE	BC	V0L 1V0	CANADA	51.9043339	-124.6057514	1 IHA	Interior	1
**PCC_0122**		TUSCANY MEDICAL CLINIC, 102, 1646 MCKENZIE AVE	VICTORIA	BC	V8N 0A3	CANADA	48.4691264	-123.3303756	4 VIHA	Vancouver Island	4
**PCC_0123**		101, 1660 FELTHAM RD	VICTORIA	BC	V8N 2A1	CANADA	48.4764944	-123.3336197	4 VIHA	Vancouver Island	3
**PCC_0124**	PO BOX 1060	168 WELD ST	PARKSVILLE	BC	V9P 2H1	CANADA	49.3190801	-124.308108	4 VIHA	Vancouver Island	4
**PCC_0125**		COMOX MEDICAL CLINIC, 100, 1695 COMOX AVE	COMOX	BC	V9M 3M1	CANADA	49.6720752	-124.9230016	4 VIHA	Vancouver Island	12

PCC_0126		BRICKYARD MEDICAL CLINIC, 17475 56 AVE	SURREY	BC	V3S 2X6	CANADA	49.104376	-122.7390628	2 FHA	Fraser	2
PCC_0127		201, 1757 BEAUFORT AVE	COMOX	BC	V9M 1R8	CANADA	49.6718576	-124.9255551	4 VIHA	Vancouver Island	6
PCC_0128		PACIFIC HIGHWAY MEDICAL CLINIC, 17660 65A AVE, UNIT 106	SURREY	BC	V3S 5N4	CANADA	49.1207061	-122.733754	2 FHA	Fraser	1
PCC_0129		101B, 1771 COMOX AVE	COMOX	BC	V9M 3L9	CANADA	49.6730416	-124.926123	4 VIHA	Vancouver Island	4
PCC_0130		MOUNTAIN VIEW MEDICAL CLINIC, 201, 1789 KLO RD	KELOWNA	BC	V1W 3P3	CANADA	49.8605705	-119.4576554	1 IHA	Interior	4
PCC_0131		AQUARIUS MEDICAL CLINIC, 179 DAVIE ST	VANCOUVER	BC	V6Z 2Y1	CANADA	49.2739437	-123.1202846	3 VCHA	Vancouver Coastal	1
PCC_0132		AQUAIRUS MEDICAL CLINIC, 201, 179 DAVIE ST	VANCOUVER	BC	V6Z 2Y1	CANADA	49.273787	-123.1198134	3 VCHA	Vancouver Coastal	1
PCC_0133		JUBILEE MEDICAL CLINIC, 1803 FORT ST	VICTORIA	BC	V8R 1J6	CANADA	48.4294267	-123.3299316	4 VIHA	Vancouver Island	7
PCC_0134		230, 181 KEEFER PL	VANCOUVER	BC	V6B 6C1	CANADA	49.2802457	-123.1083769	3 VCHA	Vancouver Coastal	4
PCC_0135		1823 FORT ST	VICTORIA	BC	V8R 1J6	CANADA	48.4294848	-123.3293751	4 VIHA	Vancouver Island	4
PCC_0136		CAPRI MEDICAL CENTRE, 1835 GORDON DR	KELOWNA	BC	V1Y 3H4	CANADA	49.8816284	-119.4757465	1 IHA	Interior	1
PCC_0137		CAPRI MEDICAL CENTRE, 132, 1835 GORDON DR	KELOWNA	BC	V1Y 3H5	CANADA	49.8816284	-119.4757465	1 IHA	Interior	5
PCC_0138		CASTLEGAR MEDICAL CLINIC, 1840 8 AVE	CASTLEGAR	BC	V1N 2Y2	CANADA	49.2989656	-117.6582702	1 IHA	Interior	4
PCC_0139		LEXITOR CLINIC, 102, 1847 DUFFERIN CRES	NANAIMO	BC	V9S 0B1	CANADA	49.1829388	-123.9835389	4 VIHA	Vancouver Island	7
PCC_0140		APPLE PLAZA CLINIC, 1848 MAIN ST	PENTICTON	BC	V2A 5H3	CANADA	49.4780227	-119.5828142	1 IHA	Interior	1
PCC_0141		186 KENNETH ST	DUNCAN	BC	V9L 1N4	CANADA	48.778342	-123.7098728	4 VIHA	Vancouver Island	10
PCC_0142	PO BOX 379	CHRISTINA LAKE MEDICAL CLINIC, 1875 BAKERY FRONTAGE RD	CHRISTINA LAKE	BC	V0H 1E0	CANADA	49.052974	-118.2113701	1 IHA	Interior	1
PCC_0143		1885 SOLLANS RD	HORNBY ISLAND	BC	V0R 1Z0	CANADA	49.53547	-124.6648743	4 VIHA	Vancouver Island	2
PCC_0144	PO BOX 1720	VILLAGE MEDICAL CLINIC, 190 HORSE LAKE RD	100 MILE HOUSE	BC	V0K 2E0	CANADA	51.6383092	-121.2959826	1 IHA	Interior	3
PCC_0145		PITT RIVER MEDICAL CENTER, 105, 19070 LOUGHEED HWY	PITT MEADOWS	BC	V3Y 2M6	CANADA	49.2312423	-122.6927321	2 FHA	Fraser	5
PCC_0146		COLWOOD MEDICAL TREATMENT CENTRE, 1910 SOOKE RD	VICTORIA	BC	V9B 1V7	CANADA	48.441496	-123.4707503	4 VIHA	Vancouver Island	1
PCC_0147		FORD RD MEDICAL CLINIC, 2, 19126 FORD RD	PITT MEADOWS	BC	V3Y 2P1	CANADA	49.2219378	-122.6919534	2 FHA	Fraser	2
PCC_0148		DOGWOOD MEDICAL CLINIC, 191, B DOGWOOD ST	CAMPBELL RIVER	BC	V9W 6B9	CANADA	50.0108711	-125.2473006	4 VIHA	Vancouver Island	3
PCC_0149		SURLANG MEDICINE CENTRE, 19475 FRASER HWY UNIT 106	SURREY	BC	V3S 6K7	CANADA	49.1156929	-122.6827397	2 FHA	Fraser	1
PCC_0150	PO BOX 610	BEAVER VALLEY CLINIC, 1957 COLUMBIA GARDENS RD	FRUITVALE	BC	V0G 1L0	CANADA	49.1150403	-117.542582	1 IHA	Interior	1
PCC_0151		ABERDEEN MEDICAL CLINIC, 1966 HARRISON WAY	KAMLOOPS	BC	V1S 1M9	CANADA	50.6494377	-120.3729734	1 IHA	Interior	12
PCC_0152		OXFORD CROSSING CLINIC, 3190, 1971 LOUGHEED HWY	PORT COQUITLAM	BC	V3B 0K2	CANADA	49.2645928	-122.7687074	2 FHA	Fraser	4
PCC_0153	PO BOX 1059	ASSOCIATE MEDICAL CLINIC, 1973 COLUMBIA AVE	ROSSLAND	BC	V0G 1Y0	CANADA	49.0767259	-117.8007222	1 IHA	Interior	4
PCC_0154		20040 40A AVE	LANGLEY	BC	V3A 2Y5	CANADA	49.0758122	-122.6684036	2 FHA	Fraser	2
PCC_0155		301, 20055 WILLOWBROOK DR	LANGLEY	BC	V2Y 2T5	CANADA	49.1165575	-122.6664047	2 FHA	Fraser	3
PCC_0156		CROSSROADS MEDICAL CLINIC, 103, 20110 LOUGHEED HWY	MAPLE RIDGE	BC	V2X 2P7	CANADA	49.2202645	-122.6636816	2 FHA	Fraser	1
PCC_0157		RICHMOND RD MEDICAL CLINIC, 101, 2020 RICHMOND RD	VICTORIA	BC	V8R 6R5	CANADA	48.4305342	-123.3302199	4 VIHA	Vancouver Island	1
PCC_0158		HEALTHWAY MEDICAL CLINIC, UNIT B 20200 FRASER HWY	LANGLEY	BC	V3A 4E6	CANADA	49.1060814	-122.6625836	2 FHA	Fraser	2
PCC_0159	BOX 998	MERRITT MEDICAL CLINIC, 2035 CHARTERS ST	MERRITT	BC	V1K 1B8	CANADA	50.1100775	-120.7846223	1 IHA	Interior	2
PCC_0160		LANGLEY YOUTH WELLNESS CLINIC, 20389 FRASER HWY	LANGLEY	BC	V3A 7N2	CANADA	49.1052313	-122.6571773	2 FHA	Fraser	1
PCC_0161		WESTGATE MEDICAL CLINIC, 402, 20395 LOUGHEED HWY	MAPLE RIDGE	BC	V2X 2P9	CANADA	49.2196856	-122.6557605	2 FHA	Fraser	5
PCC_0162		100, 205 NEWPORT DR	PORT MOODY	BC	V3H 5C9	CANADA	49.2828136	-122.827467	2 FHA	Fraser	3
PCC_0163		QUESNEL MEDICAL CLINIC, 205 ST LAURENT AVE	QUESNEL	BC	V2J 2C8	CANADA	52.9790416	-122.4968088	5 NHA	Northern	5
PCC_0164		101, 2051 MCCALLUM RD	ABBOTSFORD	BC	V2S 3N5	CANADA	49.0398624	-122.2934393	2 FHA	Fraser	5
PCC_0165		GATEWAY HEALTHCARE, 203, 2051 MCCALLUM RD	ABBOTSFORD	BC	V2S 3N5	CANADA	49.0398624	-122.2934393	2 FHA	Fraser	13
PCC_0166		PURE CARE CLINIC AND TRUE CARE CLINIC LANGLEY, 20644 FRASER HWY APT 100	LANGLEY	BC	V3A 4G5	CANADA	49.1031765	-122.6508063	2 FHA	Fraser	1
PCC_0167	PO BOX 700	MONASHEE MEDICAL CLINIC, 2067 COLUMBIA AVE	ROSSLAND	BC	V0G 1Y0	CANADA	49.07669	-117.798953	1 IHA	Interior	4
PCC_0168	PO BOX 326	20800 LOUGHEED HWY, STN DEL CENTRE	MAPLE RIDGE	BC	V2X 7G2	CANADA	49.2175452	-122.6451656	2 FHA	Fraser	1
PCC_0169		CLINE MEDICAL CENTRE, 2090 SKAHA DR	NANAIMO	BC	V9R 6R8	CANADA	49.1747993	-123.991293	4 VIHA	Vancouver Island	1
PCC_0170		COVID, 19 AND INFLUENZA, LIKE ILLNESS ASSESSMENT CENTRE, 2099 LOUGHEED HWY UNIT A120	PORT COQUITLAM	BC	V3B 1A8	CANADA	49.2667716	-122.7737774	2 FHA	Fraser	1
PCC_0171	PO BOX 250	212 ALPINE WAY	ELKFORD	BC	V0B 1H0	CANADA	50.0210092	-114.9174472	1 IHA	Interior	3
PCC_0172		CANNON CLINIC, 4, 2151 MCCALLUM RD	ABBOTSFORD	BC	V2S 3N9	CANADA	49.0409292	-122.2946068	2 FHA	Fraser	7
PCC_0173		HOY MEDICAL, 402, 2155 10TH AVE	PRINCE GEORGE	BC	V2M 5J6	CANADA	53.9152456	-122.765827	5 NHA	Northern	11
PCC_0174	PO BOX 400	PORT MCNEILL MEDICAL CLINIC, 2161 MCNEILL RD	PORT MCNEILL	BC	V0N 2R0	CANADA	50.5836711	-127.0782247	4 VIHA	Vancouver Island	4
PCC_0175		PROTEA MEDICAL CLINIC, SUITE 1, 219 FERN RD WEST	QUALICUM BEACH	BC	V9K 2M2	CANADA	49.3464455	-124.4480103	4 VIHA	Vancouver Island	1
PCC_0176	PO BOX 189	TONQUIN CLINIC, 220 FIRST	TOFINO	BC	V0R 2Z0	CANADA	49.151284	-125.9072416	4 VIHA	Vancouver Island	3
PCC_0177		TUMBLER RIDGE MEDICAL CLINIC, 220 FRONT ST	TUMBLER RIDGE	BC	V0C 2W0	CANADA	55.1244008	-121.0001792	5 NHA	Northern	1
PCC_0178		BALANCE MEDICAL CENTER, 2201 BROADWAY W	VANCOUVER	BC	V6K 2E4	CANADA	49.264168	-123.1558405	3 VCHA	Vancouver Coastal	2
PCC_0179		2207 GLENMORE RD, THE BRIDGE MEDICAL	CAMPBELL RIVER	BC	V9H 1E1	CANADA	49.8695316	-125.1286099	4 VIHA	Vancouver Island	2
PCC_0180		PRIMACY CLINIC, 100, 2210 MAIN ST	PENTICTON	BC	V2A 5H8	CANADA	49.4738742	-119.5800415	1 IHA	Interior	3
PCC_0181		303, 22112 52 AVE	LANGLEY	BC	V2Y 2M6	CANADA	49.0963994	-122.6114208	2 FHA	Fraser	5
PCC_0182	BOX 948	DR LORA CRUISE MEDICAL CLINIC, 222 FIRST ST E	REVELSTOKE	BC	V0E 2S0	CANADA	50.997573	-118.1930465	1 IHA	Interior	1
PCC_0183	PO BOX 74769	2230 WEST BROADWAY	VANCOUVER	BC	V6K 0E4	CANADA	49.2635846	-123.1566043	3 VCHA	Vancouver Coastal	6
PCC_0184		201, 2245 ABBOTT ST	KELOWNA	BC	V1Y 1E2	CANADA	49.8738804	-119.4969408	1 IHA	Interior	12
PCC_0185		100, 22470 DEWDNEY TRUNK RD	MAPLE RIDGE	BC	V2X 5Z6	CANADA	49.2208024	-122.5947354	2 FHA	Fraser	3
PCC_0186		101, 2255 ELGIN AVE	PORT COQUITLAM	BC	V3C 2B4	CANADA	49.2631761	-122.7785756	2 FHA	Fraser	7
PCC_0187		106, 22722 LOUGHEED HWY	MAPLE RIDGE	BC	V2X 2V6	CANADA	49.2162	-122.5928078	2 FHA	Fraser	4
PCC_0188		203, 2276 ISLAND HWY S, SUITE	CAMPBELL RIVER	BC	V9W 1C3	CANADA	49.9698565	-125.2143336	4 VIHA	Vancouver Island	5
PCC_0189		PRIMARY CARE ACCESS CLINIC, 101, 2296 MCCALLUM RD	ABBOTSFORD	BC	V2S 3P4	CANADA	49.0436744	-122.2929111	2 FHA	Fraser	1
PCC_0190		INTEGRATED HEALTH CLINIC, 23252 MAVIS AVE	LANGLEY	BC	V1M 2R4	CANADA	49.1702166	-122.577856	2 FHA	Fraser	1
PCC_0191		SHORELINE MEDICAL CLINIC, 2379 BEVAN AVE	SIDNEY	BC	V8L 4M9	CANADA	48.6473855	-123.4008276	4 VIHA	Vancouver Island	2
PCC_0192		RAVEN SONG PRIMARY CARE, 2450 ONTARIO ST	VANCOUVER	BC	V5T 4T7	CANADA	49.2635129	-123.1041171	3 VCHA	Vancouver Coastal	13
PCC_0193		18, 2475 DOBBIN RD	WESTBANK	BC	V4T 2E9	CANADA	49.8283801	-119.6290661	1 IHA	Interior	5
PCC_0194		2479 MCCALLUM RD	ABBOTSFORD	BC	V2S 3P8	CANADA	49.047228	-122.2936701	2 FHA	Fraser	2
PCC_0195		MCCALLUM HEALTH CENTRE, 2481 MCCALLUM RD SUITE 1C	ABBOTSFORD	BC	V2S 3P8	CANADA	49.0472544	-122.2939808	2 FHA	Fraser	1
PCC_0196		DIRECTOR, AMBLESIDE MEDICAL CENTRE, 250 16 ST	WEST VANCOUVER	BC	V7V 3R5	CANADA	49.3278445	-123.1581902	3 VCHA	Vancouver Coastal	7
PCC_0197		PONDEROSA PRIMARY CARE CENTRE, 110, 2504 SKAHA LAKE RD	PENTICTON	BC	V2A 6G1	CANADA	49.4687253	-119.5838601	1 IHA	Interior	6
PCC_0198		210, 2525 COMMERCIAL DR	VANCOUVER	BC	V5N 4C1	CANADA	49.2620106	-123.0701554	3 VCHA	Vancouver Coastal	2
PCC_0199		OCEAN PIER MEDICAL CLINIC, 2537 BEACON AVE	SIDNEY	BC	V8L 1Y3	CANADA	48.6484605	-123.3946544	4 VIHA	Vancouver Island	1
PCC_0200		207, 2537 BEACON AVE	SIDNEY	BC	V8L 1Y3	CANADA	48.6484605	-123.3946544	4 VIHA	Vancouver Island	9
PCC_0201		258 PARK DR	CLEARWATER	BC	V0E 1N1	CANADA	51.6500544	-120.037531	1 IHA	Interior	5
PCC_0202		FIRST CARE MEDICAL CENTRE, 2590 COMMERCIAL DR	VANCOUVER	BC	V5N 4C2	CANADA	49.2615027	-123.0697234	3 VCHA	Vancouver Coastal	2
PCC_0203	PO BOX 160	KIMBERLEY MEDICAL CLINIC, 260 FOURTH AVE	KIMBERLEY	BC	V1A 2Y6	CANADA	49.6900306	-115.9863649	1 IHA	Interior	4
PCC_0204		WESTVIEW MEDICAL CENTRE, 400, 2609 WESTVIEW DR	NORTH VANCOUVER	BC	V7N 4M2	CANADA	49.3330735	-123.0896829	3 VCHA	Vancouver Coastal	5
PCC_0205		WELCOME CENTRE MEDICAL CLINIC, 2610 VICTORIA DR	VANCOUVER	BC	V5N 4L2	CANADA	49.2611159	-123.0654038	3 VCHA	Vancouver Coastal	1
PCC_0206		OMA MEDICAL CLINIC, 202, 2620 COMMERCIAL DR	VANCOUVER	BC	V5N 4C4	CANADA	49.2607208	-123.0694263	3 VCHA	Vancouver Coastal	1
PCC_0207		COM EL MEDICAL CENTRE, 2620 COMMERCIAL DR UNIT 301	VANCOUVER	BC	V5N 4C4	CANADA	49.2607208	-123.0694263	3 VCHA	Vancouver Coastal	2
PCC_0208		OLIVETREE MEDICAL CLINIC, 2655 CLEARBROOK RD	ABBOTSFORD	BC	V2T 2Y6	CANADA	49.050643	-122.3383429	2 FHA	Fraser	1
PCC_0209	PO BOX 610	GINGER GOODWIN MEDICAL CLINIC, 2665 BEAUFORT AVE	CUMBERLAND	BC	V0R 1S0	CANADA	49.6198211	-125.0325448	4 VIHA	Vancouver Island	1
PCC_0210	PO BOX 610	SUITE 102, 2665 BEAUFORT AVE	CUMBERLAND	BC	V0R 1S0	CANADA	49.6198211	-125.0325448	4 VIHA	Vancouver Island	2
PCC_0211		LANGDON MEDICAL CENTRE, 108, 2669 LANGDON ST	ABBOTSFORD	BC	V2T 3L3	CANADA	49.050803	-122.3311472	2 FHA	Fraser	3
PCC_0212		120, 27 HELMCKEN RD	VICTORIA	BC	V8Z 5G5	CANADA	48.468176	-123.4281809	4 VIHA	Vancouver Island	7
PCC_0213		271 INGRAM ST, CANADA CARE CLINIC	DUNCAN	BC	V9L 1P3	CANADA	48.7794145	-123.7119816	4 VIHA	Vancouver Island	1
PCC_0214		230, 2720 MILL BAY RD	MILL BAY	BC	V0R 2P1	CANADA	48.6520419	-123.5590063	4 VIHA	Vancouver Island	9
PCC_0215		FORWARD CARE MEDICAL, EAST VAN, 2736 41ST AVE E	VANCOUVER	BC	V5R 2X1	CANADA	49.2327034	-123.0503292	3 VCHA	Vancouver Coastal	8
PCC_0216	PO BOX 1149	275 E STUART DR	FORT ST JAMES	BC	V0J 1P0	CANADA	54.4421505	-124.249522	5 NHA	Northern	3
PCC_0217		2751 CLIFFE AVE, SOUTHWOOD MEDICAL CLINIC	COURTENAY	BC	V9N 2L8	CANADA	49.6733228	-124.9835948	4 VIHA	Vancouver Island	1
PCC_0218		SOUTHWOOD MEDICAL, 1700, 2751 CLIFFE AVE	COURTENAY	BC	V9N 2L8	CANADA	49.6733228	-124.9835948	4 VIHA	Vancouver Island	3
PCC_0219		2755 TUTT ST APT 201	KELOWNA	BC	V1Y 0G1	CANADA	49.86577	-119.4894933	1 IHA	Interior	6
PCC_0220		BEVERLY MEDICAL CLINIC, 2763 BEVERLY ST	DUNCAN	BC	V9L 6X2	CANADA	48.7857453	-123.7080025	4 VIHA	Vancouver Island	1
PCC_0221		DIAMOND HEALTH CARE CENTRE, 2775 LAUREL ST, 5 FLOOR, TRANSPLANT CLINIC	VANCOUVER	BC	V5Z 1M9	CANADA	49.2615125	-123.1259879	3 VCHA	Vancouver Coastal	1
PCC_0222		6 FLOOR, DIAMOND CENTRE, 2775 LAUREL ST	VANCOUVER	BC	V5Z 1M9	CANADA	49.2615125	-123.1259879	3 VCHA	Vancouver Coastal	1
PCC_0223		GORDON AND LESLIE DIAMOND CENTER, 8169, 2775 LAUREL ST	VANCOUVER	BC	V5Z 1M9	CANADA	49.2615125	-123.1259879	3 VCHA	Vancouver Coastal	1
PCC_0224		GORDON AND LESLIE DIAMOND HEALTH CARE CENTRE, MD UNDERGRADUATE PROGRAM, 11265, 2775 LAUREL ST ST	VANCOUVER	BC	V5Z 1M9	CANADA	49.2615125	-123.1259879	3 VCHA	Vancouver Coastal	3
PCC_0225		MACDONALD PROFESSIONAL CENTRE, 104, 2786 16 AVE W	VANCOUVER	BC	V6K 4M1	CANADA	49.2573174	-123.1672901	3 VCHA	Vancouver Coastal	1
PCC_0226		WESTSHORE AVI HEALTH CENTRE, 111, 2787 JACKLIN RD	VICTORIA	BC	V9B 3X7	CANADA	48.4509967	-123.5048126	4 VIHA	Vancouver Island	3
PCC_0227		284 CITY CENTRE	KITIMAT	BC	V8C 1T6	CANADA	54.0543107	-128.6530733	5 NHA	Northern	2
PCC_0228		HORIZON MEDICAL CLINIC, 2845 CRUICKSHANK ST	ABBOTSFORD	BC	V2T 6X1	CANADA	49.0539702	-122.3246446	2 FHA	Fraser	1
PCC_0229		AMBLESIDE DERMEDICS & HEALTH CENTRE, 22, 285 17 ST	WEST VANCOUVER	BC	V7V 1J1	CANADA	49.3283565	-123.1605387	3 VCHA	Vancouver Coastal	1

**PCC_0230**		RUTLAND AURORA HEALTH CENTRE, 102, 285 AURORA CRES	KELOWNA	BC	V1X 7N6	CANADA	49.891331	-119.3975117	1 IHA	Interior	2
**PCC_0231**		RUTLAND MEDICAL ASSOCIATES, 285 AURORA CRES SUITE 200	KELOWNA	BC	V1X 7N6	CANADA	49.89122	-119.3973182	1 IHA	Interior	2
**PCC_0232**		205, 285 AURORA CRES	KELOWNA	BC	V1X 7N6	CANADA	49.891331	-119.3975117	1 IHA	Interior	3
**PCC_0233**		206, 285 AURORA CRES	KELOWNA	BC	V1X 7N6	CANADA	49.891331	-119.3975117	1 IHA	Interior	5
**PCC_0234**		KHATSALANO MEDICAL CLINIC, 2865 WEST BROADWAY	VANCOUVER	BC	V6K 2G2	CANADA	49.2643678	-123.1696071	3 VCHA	Vancouver Coastal	1
**PCC_0235**	BOX 765	SEABIRD ISLAND HEALTH CENTRE, 2895 CHOWAT RD	AGASSIZ	BC	V0M 1A0	CANADA	49.2543676	-121.7296865	2 FHA	Fraser	1
**PCC_0236**	PO BOX 879	EXETER MEDICAL CLINIC, 290 HORSE LAKE RD	100 MILE HOUSE	BC	V0K 2E1	CANADA	51.6381314	-121.2940732	1 IHA	Interior	4
**PCC_0237**		THE LIGHTHOUSE MEDICAL CLINIC, 205, 2903 32 AVE	VERNON	BC	V1T 2L6	CANADA	50.2655568	-119.2699663	1 IHA	Interior	2
**PCC_0238**	PO BOX 820	BRIDGESIDE MEDICAL CLINIC, 291 FAIRVIEW RD	OLIVER	BC	V0H 1T0	CANADA	49.1825047	-119.5486875	1 IHA	Interior	1
**PCC_0239**	PO BOX 820	107, 291C FAIRVIEW RD	OLIVER	BC	V0H 1T0	CANADA	49.1825047	-119.5486875	1 IHA	Interior	3
**PCC_0240**		3010, 2929 BARNET HWY, CAREMART	COQUITLAM	BC	V3B 5R5	CANADA	49.2792693	-122.7980479	2 FHA	Fraser	2
**PCC_0241**		LU'MA MEDICAL CENTRE, 2970 NANAIMO ST	VANCOUVER	BC	V5N 5G3	CANADA	49.2572968	-123.0565372	3 VCHA	Vancouver Coastal	3
**PCC_0242**		TSARTLIP HEALTH CENTRE, 3 BOAT RAMP RD	BRENTWOOD BAY	BC	V8M 1N9	CANADA	48.5817158	-123.4683243	4 VIHA	Vancouver Island	1
**PCC_0243**		KAMLOOPS VALLEY MEDICAL CLINIC, 206, 300 COLUMBIA ST	KAMLOOPS	BC	V2C 6L1	CANADA	50.6716375	-120.3338026	1 IHA	Interior	1
**PCC_0244**		CAREVIEW MEDICAL CENTRE, 214, 3001 GORDON AVE	COQUITLAM	BC	V3C 2K7	CANADA	49.2716022	-122.792874	2 FHA	Fraser	2
**PCC_0245**		103, 3007 GLEN DR	COQUITLAM	BC	V3B 0L8	CANADA	49.2830829	-122.7938943	2 FHA	Fraser	4
**PCC_0246**		CARE4YOU, 303, 301 COLUMBIA ST E	NEW WESTMINSTER	BC	V3L 3W5	CANADA	49.2260098	-122.8933071	2 FHA	Fraser	2
**PCC_0247**		CAMERON MEDICAL CLINIC, 302 CAMERON ST	WILLIAMS LAKE	BC	V2G 1S9	CANADA	52.1337387	-122.1438067	1 IHA	Interior	3
**PCC_0248**		ARBUTUS CLINIC, 1, 306 LOWER GANGES RD	SALT SPRING ISLAND	BC	V8K 2V3	CANADA	48.8589558	-123.5073201	4 VIHA	Vancouver Island	1
**PCC_0249**		DOCTORS MEDICAL CLINIC SUITE 215, 3066 SHELBOURNE ST	VICTORIA	BC	V8R 6T9	CANADA	48.4450083	-123.3337894	4 VIHA	Vancouver Island	1
**PCC_0250**		THE LANSDOWNE PROFESSIONAL CENTRE, 375, 3066 SHELBOURNE ST	VICTORIA	BC	V8R 6T9	CANADA	48.4450083	-123.3337894	4 VIHA	Vancouver Island	1
**PCC_0251**		DOCTORS MEDICAL CLINIC, 215, 3066 SHELBOURNE ST	VICTORIA	BC	V8R 6T9	CANADA	48.4450083	-123.3337894	4 VIHA	Vancouver Island	8
**PCC_0252**		VALLEY CARE MEDICAL CLINIC, 310 8 ST UNIT E	COURTENAY	BC	V9N 1N3	CANADA	49.6888756	-124.9971749	4 VIHA	Vancouver Island	2
**PCC_0253**		WASHINGTON PARK MEDICAL CLINIC, E, 310 8 ST	COURTENAY	BC	V9N 1N3	CANADA	49.6888756	-124.9971749	4 VIHA	Vancouver Island	9
**PCC_0254**		COWICHAN MEDICAL CENTRE, 3106 GIBBINS RD	DUNCAN	BC	V9L 1E8	CANADA	48.7835959	-123.7259008	4 VIHA	Vancouver Island	1
**PCC_0255**		ALDERGROVE MEDICAL CENTRE, 110, 3113 272 ST	ALDERGROVE	BC	V4W 3R9	CANADA	49.0592741	-122.4711786	2 FHA	Fraser	4
**PCC_0256**		2OO, 31205 MACLURE RD, BLUE OAK MEDICAL CLINIC	ABBOTSFORD	BC	V2T 5E5	CANADA	49.054562	-122.3589427	2 FHA	Fraser	1
**PCC_0257**		BLUE OAK MEDICAL CLINIC, 200, 31205 MACLURE RD	ABBOTSFORD	BC	V2T 5E5	CANADA	49.054562	-122.3589427	2 FHA	Fraser	3
**PCC_0258**		350, 3150 54 AVE E	VANCOUVER	BC	V5S 1Z1	CANADA	49.2190887	-123.040184	3 VCHA	Vancouver Coastal	9
**PCC_0259**	PO BOX 969	319 15 AVE N	CRESTON	BC	V0B 1G0	CANADA	49.0984416	-116.5084939	1 IHA	Interior	5
**PCC_0260**		108, 3195 GRANVILLE ST	VANCOUVER	BC	V6H 3K2	CANADA	49.2572074	-123.1392212	3 VCHA	Vancouver Coastal	3
**PCC_0261**		GWAII MEDICAL CLINIC, 31956 LOUGHEED HWY	MISSION	BC	V2V 1A1	CANADA	49.1318085	-122.3402049	2 FHA	Fraser	3
**PCC_0262**		CASEY MEDICAL CLINIC, 320 2 AVE W	PRINCE RUPERT	BC	V8J 1G6	CANADA	54.313895	-130.3254244	5 NHA	Northern	2
**PCC_0263**		WELLINGTON MEDICAL CLINIC, 3200 N ISLAND HIGHWAY	NANAIMO	BC	V9T 1W1	CANADA	49.2051299	-124.0055342	4 VIHA	Vancouver Island	1
**PCC_0264**		WELLINGTON MEDICAL CLINIC, 3200 ISLAND HWY N UNIT 84C	NANAIMO	BC	V9T 1W1	CANADA	49.2051299	-124.0055342	4 VIHA	Vancouver Island	10
**PCC_0265**		ORCHARD'S WALK MEDICAL CLINIC, 101, 3200 VALLEYVIEW DR	KAMLOOPS	BC	V2C 0H1	CANADA	50.6707927	-120.2252897	1 IHA	Interior	1
**PCC_0266**		102, 3200 VALLEYVIEW DR	KAMLOOPS	BC	V2C 4S2	CANADA	50.6706671	-120.2254763	1 IHA	Interior	6
**PCC_0267**		200, 3207 30TH AVE	VERNON	BC	V1T 2C6	CANADA	50.2639381	-119.2745054	1 IHA	Interior	9
**PCC_0268**		THE CENTREVILLE CLINIC, 301, 3210 25 AVE	VERNON	BC	V1T 1P1	CANADA	50.2603819	-119.2749639	1 IHA	Interior	10
**PCC_0269**		MISSION OAKS MEDICAL CENTRE, 32136 LOUGHEED HWY	MISSION	BC	V2V 1A4	CANADA	49.1316562	-122.3333503	2 FHA	Fraser	2
**PCC_0270**		270, 32156 HILLCREST AVE	ABBOTSFORD	BC	V2T 1S4	CANADA	49.0476582	-122.3330365	2 FHA	Fraser	3
**PCC_0271**		103, 32450 SIMON AVE	ABBOTSFORD	BC	V2T 4J2	CANADA	49.0527908	-122.3252165	2 FHA	Fraser	3
**PCC_0272**		MISSION HILLS MEDICAL CLINIC, 220, 32530 LOUGHEED HIGHWAY	MISSION	BC	V2V 1A5	CANADA	49.1322735	-122.3213361	2 FHA	Fraser	1
**PCC_0273**		PRANJNA MEDICAL CLINIC, 106–32615, SOUTH FRASER WAY	ABBOTSFORD	BC	V2T 1X8	CANADA	49.0518521	-122.3204469	2 FHA	Fraser	2
**PCC_0274**		ASSISTANT CLINICAL PROFESSOR, UBC FACULTY OF MEDICINE, HEATH & WELLNESS, UBC OKANAGAN, 3272 UNIVERSITY WAY, UN	KELOWNA	BC	V1V 1V7	CANADA	49.9411847	-119.3962334	1 IHA	Interior	1
**PCC_0275**		2 AVE MEDICAL CLINIC, 330 2 AVE W	PRINCE RUPERT	BC	V8J 1G6	CANADA	54.3137963	-130.3256764	5 NHA	Northern	11
**PCC_0276**		101, 330 FESTUBERT ST, ALDERLEA MEDICAL CLINIC	DUNCAN	BC	V9L 3S9	CANADA	48.7776517	-123.7016565	4 VIHA	Vancouver Island	7
**PCC_0277**		MYMD MEDICAL CLINIC, 3300 BOUNDARY RD	BURNABY	BC	V5M 0A8	CANADA	49.2544608	-123.0230517	2 FHA	Fraser	1
**PCC_0278**		VERNON DOWNTOWN PRIMARY CARE CENTRE, 3306A 32 AVE	VERNON	BC	V1T 2M6	CANADA	50.2652486	-119.2761567	1 IHA	Interior	1
**PCC_0279**		2 FLOOR, 333 SEYMOUR BLVD	NORTH VANCOUVER	BC	V7J 2J4	CANADA	49.3115536	-123.024775	3 VCHA	Vancouver Coastal	3
**PCC_0280**		OMINECA MEDICAL CLINIC, 3348 HOSPITAL RD	VANDERHOOF	BC	V0J 3A2	CANADA	54.0283168	-124.008808	5 NHA	Northern	15
**PCC_0281**		VALLEY MEDICAL CLINIC, 335 JUBILEE ST	DUNCAN	BC	V9L 1W9	CANADA	48.7795684	-123.7107508	4 VIHA	Vancouver Island	7
**PCC_0282**		DENMAN ISLAND MEDICAL CLINIC, 3351 PIERCY RD	DENMAN ISLAND	BC	V0R 1T0	CANADA	49.5372757	-124.8202163	4 VIHA	Vancouver Island	1
**PCC_0283**		BLUE SKY MEDICAL CLINIC, 341 FESTUBERT ST	DUNCAN	BC	V9L 3T1	CANADA	48.7777584	-123.702426	4 VIHA	Vancouver Island	1
**PCC_0284**		CAREXUS CLINIC, 104, 34143 MARSHALL RD	ABBOTSFORD	BC	V2S 1L8	CANADA	49.0389593	-122.278581	2 FHA	Fraser	1
**PCC_0285**		HOLLEY CLINIC, 348 FRONT ST	QUESNEL	BC	V2J 2K3	CANADA	52.9781695	-122.4978461	5 NHA	Northern	8
**PCC_0286**		350 COLUMBIA ST	VANCOUVER	BC	V6A 4J1	CANADA	49.2818492	-123.102197	3 VCHA	Vancouver Coastal	7
**PCC_0287**		HILLSIDE MEDICAL CLINIC, 360 2 AVE	CAMPBELL RIVER	BC	V9W 3T9	CANADA	50.0113995	-125.243085	4 VIHA	Vancouver Island	7
**PCC_0288**		PARKGATE MEDICAL CENTRE, 220, 3650 MT SEYMOUR PKY	NORTH VANCOUVER	BC	V7H 2Y5	CANADA	49.3162533	-122.9702535	3 VCHA	Vancouver Coastal	4
**PCC_0289**		370 TRANQUILLE RD	KAMLOOPS	BC	V2B 3G7	CANADA	50.6891273	-120.3577319	1 IHA	Interior	5
**PCC_0290**		NORTH SHORE PRIMARY CARE CENTRE, 103, 376 TRANQUILLE RD	KAMLOOPS	BC	V2B 3G7	CANADA	50.6893203	-120.357807	1 IHA	Interior	2
**PCC_0291**		CONTINENTAL MEDICAL CLINIC, 1193, 3779 SEXSMITH RD	RICHMOND	BC	V6X 3Z9	CANADA	49.1853908	-123.1295162	3 VCHA	Vancouver Coastal	1
**PCC_0292**		AFFINITY MEDICAL CLINIC, 2186, 3779 SEXSMITH RD	RICHMOND	BC	V6X 3Z9	CANADA	49.1853908	-123.1295162	3 VCHA	Vancouver Coastal	3
**PCC_0293**	PO BOX 389	SQUAMISH MEDICAL CLINIC, 37979 CLEAVELAND AVE	SQUAMISH	BC	V8B 0A3	CANADA	49.6982036	-123.1541809	3 VCHA	Vancouver Coastal	3
**PCC_0294**	PO BOX 9200	DIAMOND HEAD MEDICAL CLINIC, 37989 CLEVELAND AVE	SQUAMISH	BC	V8B 0C1	CANADA	49.6983179	-123.1540768	3 VCHA	Vancouver Coastal	3
**PCC_0295**		THE WILLOW CLINIC, 1, 3801 28A ST	VERNON	BC	V1T 9T6	CANADA	50.2699564	-119.2685361	1 IHA	Interior	3
**PCC_0296**		STEVESTON MEDICAL CENTER, 103, 3811 CHATHAM ST	RICHMOND	BC	V7E 2Z4	CANADA	49.1267089	-123.1830756	3 VCHA	Vancouver Coastal	1
**PCC_0297**		WAVECREST MEDICAL CLINIC, 106, 382 LERWICK RD	COURTENAY	BC	V9N 9E5	CANADA	49.7115827	-124.9667102	4 VIHA	Vancouver Island	4
**PCC_0298**		WHITE CLINIC, 100, 383 ELLIS ST	PENTICTON	BC	V2A 4L9	CANADA	49.4975738	-119.590922	1 IHA	Interior	5
**PCC_0299**	BOX 177	3842 THIRD AVE	SMITHERS	BC	V0J 2N3	CANADA	54.7816599	-127.1700728	5 NHA	Northern	3
**PCC_0300**		NORTH AMERICA MEDICAL CENTRE, 301, 3965 KINGSWAY	BURNABY	BC	V5H 1Y8	CANADA	49.2330893	-123.0167063	2 FHA	Fraser	3
**PCC_0301**		BLUEBIRD MEDICAL CENTER, 302B, 3975 LAKESHORE RD	KELOWNA	BC	V1W 1V3	CANADA	49.8404814	-119.4880734	1 IHA	Interior	2
**PCC_0302**		MEDICAL DIRECTOR, THE MYO CLINIC, L1, 399 TYEE RD	VICTORIA	BC	V9A 0A8	CANADA	48.4317897	-123.3786612	4 VIHA	Vancouver Island	2
**PCC_0303**		200, 4012 HASTINGS ST E	BURNABY	BC	V5C 2H9	CANADA	49.2808069	-123.0158079	2 FHA	Fraser	11
**PCC_0304**		SEA TO SKY CLINIC, 40147 GLENALDER PL	SQUAMISH	BC	V8B 0G2	CANADA	49.7368182	-123.1338681	3 VCHA	Vancouver Coastal	2
**PCC_0305**		LOTUS MEDICAL, 4018 KNIGHT ST	VANCOUVER	BC	V5N 5Y7	CANADA	49.2490433	-123.0749432	3 VCHA	Vancouver Coastal	4
**PCC_0306**	PO BOX 820	402 2 AVE	FERNIE	BC	V0B 1M0	CANADA	49.5022551	-115.0619987	1 IHA	Interior	5
**PCC_0307**		2180, 4151 HAZELBRIDGE WAY	RICHMOND	BC	V6X 4J7	CANADA	49.1838016	-123.1341126	3 VCHA	Vancouver Coastal	9
**PCC_0308**	BOX 2769	CHISEL PEAK MEDICAL CLINIC, 417 10TH AVE	INVERMERE	BC	V0A 1K0	CANADA	50.5095655	-116.0316011	1 IHA	Interior	6
**PCC_0309**		CENTRICARE MEDICAL CLINIC, 4209 FRASER ST	VANCOUVER	BC	V5V 4G1	CANADA	49.2474577	-123.0902598	3 VCHA	Vancouver Coastal	1
**PCC_0310**		BURNABY PRIMARY AND URGENT CARE CENTRE, 37M, 4277 KINGSWAY	BURNABY	BC	V5H 3Z2	CANADA	49.2316429	-123.0088961	2 FHA	Fraser	1
**PCC_0311**		4303 10TH AVE W	VANCOUVER	BC	V6R 2H6	CANADA	49.2640065	-123.2034232	3 VCHA	Vancouver Coastal	7
**PCC_0312**		4336 SUNSHINE COAST HWY, UPSTREAM MEDICAL CLINIC	SECHELT	BC	V0N 3A1	CANADA	49.4402988	-123.7094112	3 VCHA	Vancouver Coastal	1
**PCC_0313**		COAST MEDICAL WHISTLER, 110, 4350 LORIMER RD	WHISTLER	BC	V0N 1B4	CANADA	50.119201	-122.956261	3 VCHA	Vancouver Coastal	2
**PCC_0314**		RIVERVIEW MEDICAL CLINIC, 104, 436 LORNE ST	KAMLOOPS	BC	V2C 1W3	CANADA	50.6783656	-120.331077	1 IHA	Interior	3
**PCC_0315**		CORNERSTONE MEDICAL CLINIC, 4367 HASTINGS ST	BURNABY	BC	V5C 2J7	CANADA	49.2812968	-123.0064992	2 FHA	Fraser	2
**PCC_0316**		207, 437 GLENMORE RD	KELOWNA	BC	V1V 1Y5	CANADA	49.9145524	-119.4460224	1 IHA	Interior	5
**PCC_0317**		MARTIN ST OUTREACH CENTRE, 117, 437 MARTIN ST	PENTICTON	BC	V2A 5L1	CANADA	49.4961108	-119.5929126	1 IHA	Interior	5
**PCC_0318**		1550, 4380 NO 3	RICHMOND	BC	V6X 3V7	CANADA	49.1815473	-123.135047	3 VCHA	Vancouver Coastal	3
**PCC_0319**		COMER MEDICAL CLINIC, 440 COMER ST	WILLIAMS LAKE	BC	V2G 1T7	CANADA	52.1363067	-122.1443111	1 IHA	Interior	3
**PCC_0320**		CROWN ISLE CLINIC, 300, 444 LERWICK RD	COURTENAY	BC	V9N 0A9	CANADA	49.7103498	-124.9656984	4 VIHA	Vancouver Island	5
**PCC_0321**		CONCORD MEDICAL CENTRE, 4484 MAIN ST	VANCOUVER	BC	V6V 3R3	CANADA	49.2446766	-123.1012123			1
**PCC_0322**		36, 450 LANSDOWNE ST, KAMLOOPS DOWNTOWN HEALTH CENTRE	KAMLOOPS	BC	V2C 1Y3	CANADA	50.6770071	-120.3291751	1 IHA	Interior	2
**PCC_0323**	PO BOX 799	BARRIERE HEALTH CENTRE, 4537 BARRIERE TOWN RD	BARRIERE	BC	V0E 1E0	CANADA	51.1802754	-120.1253689	1 IHA	Interior	2
**PCC_0324**		OUTREACH URBAN HEALTH CLINIC, INTERIOR HEALTH, 455 LEON AVE	KELOWNA	BC	V1Y 6J4	CANADA	49.8843076	-119.4941692	1 IHA	Interior	3
**PCC_0325**		FALSE CREEK HEALTH CENTRE, 4555 8 AVE W, FLOOR 555	VANCOUVER	BC	V6R 2A4	CANADA	49.2656316	-123.2107023	3 VCHA	Vancouver Coastal	1
**PCC_0326**		GARRISON MEDICAL CLINIC, UNIT 2, 45555 MARKET WAY	CHILLIWACK	BC	V2R 0M5	CANADA	49.1047018	-121.9637872	2 FHA	Fraser	1
**PCC_0327**		METRO MEDICAL CLINIC, 45610 YALE RD UNIT 202	CHILLIWACK	BC	V2P 2N2	CANADA	49.1534866	-121.9624544	2 FHA	Fraser	2
**PCC_0328**		HIGHROADS MEDICAL CLINICS, 4588 DUNBAR ST	VANCOUVER	BC	V6S 2G6	CANADA	49.2453064	-123.1851967	3 VCHA	Vancouver Coastal	6
**PCC_0329**		1, 45905 YALE RD	CHILLIWACK	BC	V2P 2M6	CANADA	49.1675973	-121.9583882	2 FHA	Fraser	4
**PCC_0330**		HG HEALTH CENTRE, 4610 LAZELLE AVE	TERRACE	BC	V8G 1S5	CANADA	54.5176116	-128.5877701	5 NHA	Northern	6
**PCC_0331**		SPRUCE MEDICAL CENTRE, 102, 4630 LAZELLE AVE	TERRACE	BC	V8G 1S6	CANADA	54.5175215	-128.5891663	5 NHA	Northern	8
**PCC_0332**		4 FLOOR, PARK AVE MEDICAL CLINIC, 400, 4634 PARK AVE	TERRACE	BC	V8G 1V7	CANADA	54.5189669	-128.5910788	5 NHA	Northern	1
**PCC_0333**		PARK AVE MEDICAL CENTRE, 400, 4634 PARK AVE	TERRACE	BC	V8G 1V7	CANADA	54.5189669	-128.5910788	5 NHA	Northern	5

**PCC_0334**		4645 KINGSWAY	BURNABY	BC	V5H 2B3	CANADA	49.2291662	-123.0006352	2 FHA	Fraser	5
**PCC_0335**		IMPERIAL MEDICAL CLINIC, 4648 IMPERIAL ST	BURNABY	BC	V5J 1B8	CANADA	49.2217077	-122.9987149	2 FHA	Fraser	8
**PCC_0336**		INTEGRATED WELLNESS MEDICAL CLINIC, 4655 CENTRAL BLVD	BURNABY	BC	V5H 4H7	CANADA	49.2239592	-122.9986929	2 FHA	Fraser	3
**PCC_0337**		INTEGRATED WELLNESS MEDICAL CENTRE, 4655 CENTRAL BLVD UNIT 2	BURNABY	BC	V5H 4H7	CANADA	49.2239592	-122.9986929	2 FHA	Fraser	4
**PCC_0338**		101, 4695 HASTINGS ST	BURNABY	BC	V5C 2K6	CANADA	49.2812538	-122.9978158	2 FHA	Fraser	8
**PCC_0339**		THE MEDICAL CLINIC ASSOCIATES, 4794 JOYCE AVE	POWELL RIVER	BC	V8A 3B6	CANADA	49.8456464	-124.5200536	3 VCHA	Vancouver Coastal	13
**PCC_0340**		480 10TH AVE	CAMPBELL RIVER	BC	V9W 4E3	CANADA	50.0252504	-125.2464419	4 VIHA	Vancouver Island	4
**PCC_0341**		METROHEALTH CLINIC, 4879 KINGSWAY	BURNABY	BC	V5H 4T6	CANADA	49.2274441	-122.994761	2 FHA	Fraser	3
**PCC_0342**		OCEANSIDE HEALTH CENTRE, 489 ALBERNI HWY	PARKSVILLE	BC	V9P 1J9	CANADA	49.3105943	-124.323014	4 VIHA	Vancouver Island	8
**PCC_0343**		DELTA MEDICAL CLINIC, 4977 TRENANT ST	DELTA	BC	V4K 2K5	CANADA	49.0923722	-123.0856466	2 FHA	Fraser	2
**PCC_0344**		PARK AVE MEDICAL CLINIC, 4 FLOOR, TH	TERRACE	BC	V8G 1V7	CANADA	54.0586216	-128.6877284	5 NHA	Northern	1
**PCC_0345**	PO BOX 1000	LOGAN LAKE HEALTH CENTRE, 5 BERYL DR	LOGAN LAKE	BC	V0K 1W0	CANADA	50.4954097	-120.8124342	1 IHA	Interior	3
**PCC_0346**		MIDWAY MEDICAL CLINIC, 500 HAYNES ST	MIDWAY	BC	V0H 1M0	CANADA	49.0068943	-118.7704068	1 IHA	Interior	1
**PCC_0347**		BURQUITLAM MEDICAL CLINIC, 504 COTTONWOOD AVE	COQUITLAM	BC	V3J 2R5	CANADA	49.2586143	-122.8924151	2 FHA	Fraser	1
**PCC_0348**		BURQUITLAM MEDICAL CLINIC, 100B, 504 COTTONWOOD AVE	COQUITLAM	BC	V3J 2R5	CANADA	49.2586143	-122.8924151	2 FHA	Fraser	5
**PCC_0349**		CROSSROADS CLINICS, 3 FLOOR, 507 BROADWAY ST W	VANCOUVER	BC	V5Z 1E6	CANADA	49.2637082	-123.1157821	3 VCHA	Vancouver Coastal	1
**PCC_0350**		PACIFIC STATION MEDICAL CLINIC, 104, 5160 DUBLIN WAY	NANAIMO	BC	V9T 0H2	CANADA	49.2170015	-124.0327075	4 VIHA	Vancouver Island	5
**PCC_0351**		5317 HEADLAND DR	WEST VANCOUVER	BC	V7W 3C5	CANADA	49.3503891	-123.2531819	3 VCHA	Vancouver Coastal	3
**PCC_0352**	PO BOX 700	HILLSIDE MEDICAL CENTRE, 541 3 AVE	LADYSMITH	BC	V9G 1A5	CANADA	48.9921793	-123.8203421	4 VIHA	Vancouver Island	8
**PCC_0353**		AVI HEALTH CENTRE, 102, 55 VICTORIA RD	NANAIMO	BC	V9R 5N9	CANADA	49.1603538	-123.9350276	4 VIHA	Vancouver Island	1
**PCC_0354**		216 55 VICTORIA RD, TREW BEGINNINGS CLINIC	NANAIMO	BC	V9R 4N9	CANADA	49.1608625	-123.9347858	4 VIHA	Vancouver Island	1
**PCC_0355**		ARC CLINIC, 200, 55 VICTORIA RD	NANAIMO	BC	V9R 5N9	CANADA	49.1607003	-123.9347793	4 VIHA	Vancouver Island	2
**PCC_0356**		STEIN MEDICAL CLINIC, 188, 550 BURRARD ST	VANCOUVER	BC	V6C 2B5	CANADA	49.2857132	-123.1181517	3 VCHA	Vancouver Coastal	1
**PCC_0357**		CITY SQUARE MEDICAL CENTRE, 550 BROADWAY W	VANCOUVER	BC	V5Z 1E9	CANADA	49.2629359	-123.1165444	3 VCHA	Vancouver Coastal	1
**PCC_0358**		LITTLE MOUNTAIN MEDICAL CLINIC, 355, 550 BROADWAY W	VANCOUVER	BC	V5Z 0E9	CANADA	49.2629359	-123.1165444	3 VCHA	Vancouver Coastal	2
**PCC_0359**		CITY SQUARE MEDICAL CENTRE, 307, 550 BROADWAY W, SUITE	VANCOUVER	BC	V5Z 0E9	CANADA	49.2629359	-123.1165444	3 VCHA	Vancouver Coastal	3
**PCC_0360**		731, 550 BROADWAY W	VANCOUVER	BC	V5Z 0E9	CANADA	49.2629359	-123.1165444	3 VCHA	Vancouver Coastal	6
**PCC_0361**		FOUR OAKS MEDICAL CENTER, 5500 206 ST N	LANGLEY	BC	V3A 8R3	CANADA	49.1026749	-122.6516613	2 FHA	Fraser	5
**PCC_0362**	PO BOX 638	SECHELT MEDICAL ASSOCIATES, 102, 5531 INLET AVE	SECHELT	BC	V0N 3A0	CANADA	49.4729755	-123.7564763	3 VCHA	Vancouver Coastal	1
**PCC_0363**	PO BOX 638	SECHELT MEDICAL ASSOCIATES, 5531 INLET AVE	SECHELT	BC	V0N 3A0	CANADA	49.4729755	-123.7564763	3 VCHA	Vancouver Coastal	5
**PCC_0364**		FALSE CREEK HEALTHCARE CENTRE, 555 8 AVE W, FLOOR 6	VANCOUVER	BC	V5Z 1C6	CANADA	49.2643964	-123.116591	3 VCHA	Vancouver Coastal	1
**PCC_0365**		FALSE CREEK MEDICAL CENTER, 4 FLOOR, 555 W 8 AVE	VANCOUVER	BC	V5Z 1C6	CANADA	49.2643964	-123.116591	3 VCHA	Vancouver Coastal	1
**PCC_0366**		559 6 ST	COURTENAY	BC	V9N 1M5	CANADA	49.688887	-125.0023425	4 VIHA	Vancouver Island	6
**PCC_0367**		VICTORIA SQUARE MEDICAL CENTRE, 5619 VICTORIA DR	VANCOUVER	BC	V5P 3W2	CANADA	49.2332534	-123.0655836	3 VCHA	Vancouver Coastal	3
**PCC_0368**		5655 CAMBIE ST	VANCOUVER	BC	V5Z 3A4	CANADA	49.2340007	-123.1167767	3 VCHA	Vancouver Coastal	3
**PCC_0369**		DOWNTOWN COMMUNITY HEALTH CLINIC, 569 POWELL ST	VANCOUVER	BC	V6A 1G8	CANADA	49.2833039	-123.0917898	3 VCHA	Vancouver Coastal	8
**PCC_0370**	PO BOX 250	102, 5699 COWRIE ST	SECHELT	BC	V0N 3A0	CANADA	49.472274	-123.7582486	3 VCHA	Vancouver Coastal	3
**PCC_0371**	PO BOX 250	COWRIE MEDICAL CLINIC, 102, 5699 COWRIE ST, 102, 5699 COWRIE ST	SECHELT	BC	V0N 3A0	CANADA	49.472274	-123.7582486	3 VCHA	Vancouver Coastal	6
**PCC_0372**	PO BOX 1120	570 PINE AVE	SPARWOOD	BC	V0B 2G0	CANADA	49.7215776	-114.8923638	1 IHA	Interior	5
**PCC_0373**	PO BOX 1282	5713 DOLPHIN ST	SECHELT	BC	V0N 3A0	CANADA	49.4741072	-123.7585321	3 VCHA	Vancouver Coastal	4
**PCC_0374**		HOLLYBURN MEDICAL CENTRE, 307, 575 16 ST	WEST VANCOUVER	BC	V7V 4Y1	CANADA	49.3289966	-123.1595365	3 VCHA	Vancouver Coastal	1
**PCC_0375**		HOLLYBURN MEDICAL CENTRE, 207D, 575 16 ST	WEST VANCOUVER	BC	V7V 4Y1	CANADA	49.3289966	-123.1595365	3 VCHA	Vancouver Coastal	3
**PCC_0376**		577 5 AVE	KAMLOOPS	BC	V2C 3R1	CANADA	50.6724355	-120.3292991	1 IHA	Interior	3
**PCC_0377**		ACQUA MEDICAL CENTRE, 118, 5811 NO 3	RICHMOND	BC	V6X 4L7	CANADA	49.1724164	-123.1375566	3 VCHA	Vancouver Coastal	3
**PCC_0378**		582 GOLDSTREAM AVE UNIT 104	VICTORIA	BC	V9B 2W7	CANADA	48.4468276	-123.4871348	4 VIHA	Vancouver Island	5
**PCC_0379**		59 PENDER ST W	VANCOUVER	BC	V6B 1R3	CANADA	49.2812296	-123.1067763	3 VCHA	Vancouver Coastal	10
**PCC_0380**		WEST COLUMBIA MEDICAL CENTRE, 203, 595 COLUMBIA ST W	KAMLOOPS	BC	V2C 1K7	CANADA	50.6723644	-120.3528973	1 IHA	Interior	1
**PCC_0381**		UBC HEALTH CLINIC, 320, 5950 UNIVERSITY BLVD	VANCOUVER	BC	V6T 1Z3	CANADA	49.2658095	-123.2462171	3 VCHA	Vancouver Coastal	2
**PCC_0382**		HIGHROADS MEDICAL CLINICS KERRISDALE, 5960 EAST BOULEVARD	VANCOUVER	BC	V6M 3V4	CANADA	49.2322108	-123.1550281	3 VCHA	Vancouver Coastal	3
**PCC_0383**		102 5990 FRASER ST	VANCOUVER	BC	V5W 2Z7	CANADA	49.2304857	-123.0902114	3 VCHA	Vancouver Coastal	10
**PCC_0384**		KOOTENAY MEDICAL CENTRE, 601 KOOTENAY ST	NELSON	BC	V1L 1L1	CANADA	49.4901652	-117.2956268	1 IHA	Interior	6
**PCC_0385**	PO BOX 400	GOLD RIVER HEALTH CENTRE, 601 TRUMPETER DR	GOLD RIVER	BC	VOP 1G0	CANADA	49.7807229	-126.0516239			2
**PCC_0386**		140, 6011 NO 1 RD	RICHMOND	BC	V7C 1T4	CANADA	49.1697706	-123.1817142	3 VCHA	Vancouver Coastal	5
**PCC_0387**		EXPRESS CARE MEDICAL CLINIC, 25, 6014 VEDDER RD	CHILLIWACK	BC	V2R 5M4	CANADA	49.1144022	-121.9573526	2 FHA	Fraser	1
**PCC_0388**		MISSION PARK MALL, MISSION PARK MEDICAL CENTRE, 11, 605 KLO RD	KELOWNA	BC	V1Y 8E7	CANADA	49.8617421	-119.4888808	1 IHA	Interior	1
**PCC_0389**		MEDICAL CLINIC, 6111 RIVER RD	RICHMOND	BC	V7C 0A2	CANADA	49.1747959	-123.1515328	3 VCHA	Vancouver Coastal	1
**PCC_0390**		HEALTH CONNECTIONS CLINIC, 615 10TH ST	COURTENAY	BC	V9N 1R2	CANADA	49.6862324	-125.0002666	4 VIHA	Vancouver Island	2
**PCC_0391**	PO BOX 70	6173 KOOTENAY ST	OLIVER	BC	V0H 1T0	CANADA	49.1825723	-119.5514712	1 IHA	Interior	3
**PCC_0392**		6184 FRASER ST, PACIFIC MEDICAL CENTRE	VANCOUVER	BC	V5W 3A1	CANADA	49.2285234	-123.0902657	3 VCHA	Vancouver Coastal	3
**PCC_0393**		STATION SQUARE MEDICAL CLINIC, 6200 MCKAY AVE	BURNABY	BC	V5H 4L7	CANADA	49.2268691	-123.0042019	2 FHA	Fraser	1
**PCC_0394**		DOWNTOWN EASTSIDE CONNECTIONS CLINIC, 623 POWELL ST	VANCOUVER	BC	V6A 1H2	CANADA	49.2830574	-123.0908314	3 VCHA	Vancouver Coastal	2
**PCC_0395**		304, 625 FIFTH AVE	NEW WESTMINSTER	BC	V3M 1X4	CANADA	49.211409	-122.9200988	2 FHA	Fraser	2
**PCC_0396**		HOPE TO HEALTH RESEARCH AND INNOVATION CLINIC, 625 POWELL ST	VANCOUVER	BC	V6A 1H2	CANADA	49.2832949	-123.0907871	3 VCHA	Vancouver Coastal	4
**PCC_0397**		MAIN ST MEDICAL ASSOCIATES, 6307 MAIN ST	OLIVER	BC	V0H 1T0	CANADA	49.1842832	-119.5500087	1 IHA	Interior	1
**PCC_0398**		LANGLEY CROSSING MEDICAL CENTRE, 201, 6339 200 TH ST	LANGLEY	BC	V2Y 1A2	CANADA	49.11817	-122.6704536	2 FHA	Fraser	2
**PCC_0399**		644 FRONT ST	QUESNEL	BC	V2J 2K8	CANADA	52.9832085	-122.4985814	5 NHA	Northern	5
**PCC_0400**		CARETEL MEDICAL CLINIC, 6447 FRASER ST	VANCOUVER	BC	V5W 3A6	CANADA	49.2260278	-123.0908069	3 VCHA	Vancouver Coastal	2
**PCC_0401**		MEDICAL ARTS CENTRE, 30, 650 TERMINAL AVE S	NANAIMO	BC	V9R 5E2	CANADA	49.1642304	-123.9333756	4 VIHA	Vancouver Island	1
**PCC_0402**		OAKRIDGE CENTRE, MED/DENTAL BLDG, 226, 650 41ST AVE W	VANCOUVER	BC	V5Z 2M9	CANADA	49.2319917	-123.1191254	3 VCHA	Vancouver Coastal	1
**PCC_0403**		SOUTH TOWER, 222, 650 41 AVE W	VANCOUVER	BC	V5Z 2M9	CANADA	49.2319917	-123.1191254	3 VCHA	Vancouver Coastal	2
**PCC_0404**		OAKRIDGE CENTRE, SOUTH TOWER, 203, 650 41 AVE W	VANCOUVER	BC	V5Z 2M9	CANADA	49.2319917	-123.1191254	3 VCHA	Vancouver Coastal	4
**PCC_0405**		OAKRIDGE CENTRE, SOUTH TOWER, SUITE 312, 650 41ST AVE W	VANCOUVER	BC	V5Z 2M9	CANADA	49.2319917	-123.1191254	3 VCHA	Vancouver Coastal	7
**PCC_0406**		OAKRIDGE, FORWARD CARE MEDICAL CLINIC, 209, 650 41ST AVE W	VANCOUVER	BC	V5Z 2M9	CANADA	49.2319917	-123.1191254	3 VCHA	Vancouver Coastal	9
**PCC_0407**		FRASERVIEW MEDICAL ASSOCIATES, 665 FRONT ST	QUESNEL	BC	V2J 5J5	CANADA	52.9830269	-122.4994183	5 NHA	Northern	1
**PCC_0408**		FRASERVIEW CLINIC, 32, 665 FRONT ST	QUESNEL	BC	V2J 5J5	CANADA	52.9830269	-122.4994183	5 NHA	Northern	5
**PCC_0409**		GABRIOLA COMMUNITY HEALTH CENTRE, 695 CHURCH ST	GABRIOLA	BC	V0R 1X3	CANADA	49.1785071	-123.8363367	4 VIHA	Vancouver Island	5
**PCC_0410**		SNAW, NAW, AS HEALTH CENTRE, 700 CAPILANO RD	LANTZVILLE	BC	V0R 2H0	CANADA	49.2489671	-124.1232206	4 VIHA	Vancouver Island	1
**PCC_0411**		702, 1228 MARINASIDE CENTRE	VANCOUVER	BC	V6Z 2W4	CANADA	49.1801403	-123.9470898	3 VCHA	Vancouver Coastal	1
**PCC_0412**	PO BOX 159	7040 CHEAM AVE	AGASSIZ	BC	V0M 1A3	CANADA	49.2381485	-121.7648095	2 FHA	Fraser	5
**PCC_0413**		COOL AID COMMUNITY HEALTH SERVICES, 1 FLOOR, 713 JOHNSON ST	VICTORIA	BC	V8W 1M8	CANADA	48.4268521	-123.3639443	4 VIHA	Vancouver Island	3
**PCC_0414**		COOL AID COMMUNITY HEALTH CLINIC, 713 JOHNSON ST	VICTORIA	BC	V8W 1M8	CANADA	48.4268521	-123.3639443	4 VIHA	Vancouver Island	6
**PCC_0415**	PO BOX 476	LANTZVILLE MEDICAL CLINIC, 7175 LANTZVILLE RD	LANTZVILLE	BC	V0R 2H0	CANADA	49.249061	-124.0739584	4 VIHA	Vancouver Island	1
**PCC_0416**	PO BOX 190	SOW'S EAR MEDICAL CLINIC, 7186 LANTZVILLE RD	LANTZVILLE	BC	V0R 2H0	CANADA	49.2504312	-124.0740526	4 VIHA	Vancouver Island	8
**PCC_0417**		HARMONY COURT CARE CENTER, 7197 CANADA WAY, 2 FLOOR	BURNABY	BC	V5E 4A6	CANADA	49.2246217	-122.941799	2 FHA	Fraser	1
**PCC_0418**		7297 MAIN ST	VANCOUVER	BC	V5X 3J3	CANADA	49.2185799	-123.1019728	3 VCHA	Vancouver Coastal	3
**PCC_0419**		MISSION OAKS MEDICAL CENTRE, 7298 HURD ST, 2 FLOOR	MISSION	BC	V2V 3H5	CANADA	49.1348623	-122.3319461	2 FHA	Fraser	2
**PCC_0420**		NEW CANADIAN CLINIC, BURNABY, 204, 7315 EDMONDS ST	BURNABY	BC	V3N 1A7	CANADA	49.2188456	-122.9516147	2 FHA	Fraser	1
**PCC_0421**		PRIMECARE MEDICAL CENTRE, 202, 7315 EDMONDS ST	BURNABY	BC	V3N 1A7	CANADA	49.2188456	-122.9516147	2 FHA	Fraser	13
**PCC_0422**		ASANTE CENTRE PRIMARY CARE CLINIC, SURREY OFFICE, 303, 7337 137 ST	SURREY	BC	V3W 1A4	CANADA	49.1360178	-122.8430695	2 FHA	Fraser	1
**PCC_0423**	PO BOX 1000	HOPE MEDICAL CENTRE, 735 4 AVE	HOPE	BC	V0X 1L0	CANADA	49.3814862	-121.4398775	2 FHA	Fraser	9
**PCC_0424**		WESTWIND MEDICAL CLINIC, 107, 735 GOLDSTREAM AVE	VICTORIA	BC	V9B 2X4	CANADA	48.4476792	-123.4979688	4 VIHA	Vancouver Island	1
**PCC_0425**		7380 WESTMINSTER HWY, MINORU MEDICAL CLINIC	RICHMOND	BC	V6X 1A1	CANADA	49.1698938	-123.1416193	3 VCHA	Vancouver Coastal	1
**PCC_0426**		IN, PHASE CARE CLINIC, 7381 HURD ST	MISSION	BC	V2V 3H8	CANADA	49.1361325	-122.3329837	2 FHA	Fraser	1
**PCC_0427**		BURNS LAKE MEDICAL CLINIC, 744 CENTER ST	BURNS LAKE	BC	V0J 1E0	CANADA	54.2356547	-125.7589747	5 NHA	Northern	3
**PCC_0428**	PO BOX 400	GRAND FORKS MEDICAL CLINIC, 7486 2 ST	GRAND FORKS	BC	V0H 1H0	CANADA	49.0348458	-118.4378994	1 IHA	Interior	3
**PCC_0429**		EDMONDS MEDICAL CLINIC, C, 7487 EDMONDS ST	BURNABY	BC	V3N 1B3	CANADA	49.221894	-122.9456905	2 FHA	Fraser	1
**PCC_0430**		912, 750 BROADWAY W	VANCOUVER	BC	V5Z 1H8	CANADA	49.2630118	-123.1210731	3 VCHA	Vancouver Coastal	4
**PCC_0431**		MEDICAL CLINIC, 7511 120 ST APT 105	DELTA	BC	V4C 0C1	CANADA	49.1396287	-122.8909504	2 FHA	Fraser	2
**PCC_0432**		107, 755 GOLDSTREAM AVE	LANGFORD	BC	V9B 2X4	CANADA	48.4480525	-123.4985025	4 VIHA	Vancouver Island	5
**PCC_0433**		BURNABY MEDICAL CLINIC, 7568 6 ST	BURNABY	BC	V3N 3M3	CANADA	49.223172	-122.9323184	2 FHA	Fraser	1
**PCC_0434**		MEDCARE PLUS MEDICAL CLINIC, 7618 6 ST	BURNABY	BC	V3N 0G6	CANADA	49.2225183	-122.9315105	2 FHA	Fraser	2
**PCC_0435**		KINGSFOIL CLINIC, 764 BERNARD AVE	KELOWNA	BC	V1Y 6P5	CANADA	49.8866003	-119.4867304	1 IHA	Interior	2
**PCC_0436**		ANNE VOGEL CLINIC, 7671 ALDERBRIDGE WAY	RICHMOND	BC	V6X 1Z9	CANADA	49.1746793	-123.1444101	3 VCHA	Vancouver Coastal	1
**PCC_0437**		THE DOCTORS' OFFICE, 103, 777 BROADWAY W	VANCOUVER	BC	V5Z 4J7	CANADA	49.2636141	-123.1213655	3 VCHA	Vancouver Coastal	1

PCC_0438	BOX 330	OSOYOOS MEDICAL CENTRE, 7802 SPARTAN DR	OSOYOOS	BC	V0H 1V2	CANADA	49.0341854	-119.4641714	1 IHA	Interior	4
PCC_0439		788 GRANT AVE	COURTENAY	BC	V9N 2T3	CANADA	49.6879278	-125.0007285	4 VIHA	Vancouver Island	7
PCC_0440		ORCHID MEDICAL CLINIC, UNIT D120, 805 BOYD ST	NEW WESTMINSTER	BC	V3M 5X2	CANADA	49.192676	-122.9475427	2 FHA	Fraser	1
PCC_0441		TRU MEDICAL CLINIC, 805 TRU WAY	KAMLOOPS	BC	V2C 0C8	CANADA	50.672534	-120.3703013	1 IHA	Interior	1
PCC_0442		105, 8056 KING GEORGE BLVD	SURREY	BC	V3W 5B5	CANADA	49.1490121	-122.8452577	2 FHA	Fraser	3
PCC_0443		806 VERNON ST	NELSON	BC	V1L 4G5	CANADA	49.49425	-117.28996	1 IHA	Interior	9
PCC_0444		COPEMAN HEALTHCARE CENTRE, 200, 808 NELSON ST	VANCOUVER	BC	V6Z 2H2	CANADA	49.2800024	-123.124897	3 VCHA	Vancouver Coastal	1
PCC_0445		SUITE 300, 808 NELSON ST	VANCOUVER	BC	V6Z 2H2	CANADA	49.2800024	-123.124897	3 VCHA	Vancouver Coastal	20
PCC_0446		OAKMONT MEDICAL CENTRE, 809 41ST AVE W, SUITE 270, 809 WEST 41ST	VANCOUVER	BC	V5Z 2N6	CANADA	49.2341301	-123.1243196	3 VCHA	Vancouver Coastal	1
PCC_0447		VANCOUVER COASTAL HEALTH, GILWEST CLINIC, 8100 GRANVILLE AVE	RICHMOND	BC	V6Y 3T6	CANADA	49.1621702	-123.1347193	3 VCHA	Vancouver Coastal	2
PCC_0448		MEDICAL CLINIC, 815 CAMBIE ST	VANCOUVER	BC	V6B 2P4	CANADA	49.2781424	-123.1156127	3 VCHA	Vancouver Coastal	2
PCC_0449		816 103 AVE	DAWSON CREEK	BC	V1G 2E9	CANADA	55.7569231	-120.2259247	5 NHA	Northern	3
PCC_0450		VICTORIA YOUTH CLINIC, 3 FLOOR, 818 DOUGLAS ST	VICTORIA	BC	V8W 2B6	CANADA	48.4227881	-123.3662486	4 VIHA	Vancouver Island	2
PCC_0451		VICTORIA YOUTH CLINIC, 818 DOUGLAS ST	VICTORIA	BC	V8W 2B6	CANADA	48.4227881	-123.3662486	4 VIHA	Vancouver Island	2
PCC_0452		8195 120 ST	DELTA	BC	V4C 6P7	CANADA	49.1513153	-122.8918962	2 FHA	Fraser	3
PCC_0453		SOLIS INTEGRATIVE HEALTH CENTRE, 823 BAKER ST	NELSON	BC	V1L 4J8	CANADA	49.4941951	-117.2889418	1 IHA	Interior	1
PCC_0454		8249 EAGLE LANDING PKY UNIT 620	CHILLIWACK	BC	V2R 0P9	CANADA	49.1527121	-121.978137	2 FHA	Fraser	2
PCC_0455	PO BOX 1170	GOLDEN MEDICAL CLINIC, 826 9 AVE	GOLDEN	BC	V0A 1H0	CANADA	51.2970109	-116.9655912	1 IHA	Interior	9
PCC_0456	BOX 1170	GOLDEN MEDICAL CLINIC, 826 9 ST N	GOLDEN	BC	V0A 1H2	CANADA	51.3058846	-116.9741356	1 IHA	Interior	3
PCC_0457	PO BOX 410	826 THOMPSON AVE	CHASE	BC	V0E 1M1	CANADA	50.8186279	-119.6854186	1 IHA	Interior	2
PCC_0458		DESERT DOCTORS CLINIC, 8305 78 AVE	OSOYOOS	BC	V0H 1V0	CANADA	49.0338638	-119.4644495	1 IHA	Interior	1
PCC_0459		IMPERIAL FAMILY HEALTH CENTRE, 8318 120 ST UNIT 309, 120TH ST	SURREY	BC	V3W 3N4	CANADA	49.1543914	-122.8898401	2 FHA	Fraser	1
PCC_0460	PO BOX 159	SADDLE MOUNTAIN MEDICAL CLINIC, 2, 84 BROADWAY ST	NAKUSP	BC	V0G 1R0	CANADA	50.2384139	-117.7981406	1 IHA	Interior	1
PCC_0461		84 HASTINGS ST W, PROVIDENCE CROSSTOWN CLINIC	VANCOUVER	BC	V6B 1G6	CANADA	49.2818947	-123.1069414	3 VCHA	Vancouver Coastal	2
PCC_0462		SOUTH COWICHAN MEDICAL CLINIC, 845 DELOUME RD UNIT A	MILL BAY	BC	V0R 2P2	CANADA	48.6516588	-123.5615611	4 VIHA	Vancouver Island	1
PCC_0463		SOUTH COWICHAN MEDICAL CLINIC, 845 DELOUME RD, UNIT A	MILL BAY	BC	V0R 2P2	CANADA	48.6516588	-123.5615611	4 VIHA	Vancouver Island	2
PCC_0464		MEDLIFE MEDICAL CLINIC, 108, 845 MARINE DR	NORTH VANCOUVER	BC	V7P 0A8	CANADA	49.3208076	-123.0964231	3 VCHA	Vancouver Coastal	3
PCC_0465		PARSA MEDICAL CLINIC, 850 MARINE DR	NORTH VANCOUVER	BC	V7P 1M5	CANADA	49.3220884	-123.0964942	3 VCHA	Vancouver Coastal	1
PCC_0466		851 SEYMOUR ST	KAMLOOPS	BC	V2C 2H6	CANADA	50.6747136	-120.3198926	1 IHA	Interior	5
PCC_0467		HAPPY FACE MEDICAL CLINIC, UNIT 7, 8590 200ST	LANGLEY	BC	V2Y 2B9	CANADA	49.1584631	-122.6670334	2 FHA	Fraser	2
PCC_0468		MARPOLE MEDICAL CLINIC, 8675 GRANVILLE ST	VANCOUVER	BC	V6P 5A3	CANADA	49.2073962	-123.1409718	3 VCHA	Vancouver Coastal	5
PCC_0469		8679 120 ST	DELTA	BC	V4C 6R4	CANADA	49.1612473	-122.8904775	2 FHA	Fraser	2
PCC_0470		130, 8780 BLUNDELL RD	RICHMOND	BC	V6Y 3Y8	CANADA	49.1549946	-123.1257594	3 VCHA	Vancouver Coastal	2
PCC_0471		SUITE 202, 888 8 AVE W	VANCOUVER	BC	V5Z 3Y1	CANADA	49.2640136	-123.1235113	3 VCHA	Vancouver Coastal	4
PCC_0472		SIMON FRASER UNIVERSITY, HEALTH & COUNSELLING SERVICES, 8888 UNIVERSITY DR	BURNABY	BC	V5A 1S6	CANADA	49.2765276	-122.9183303	2 FHA	Fraser	6
PCC_0473		NORTH DELTA MEDICAL CLINIC, 8919 120 ST	DELTA	BC	V4C 6R6	CANADA	49.1654776	-122.8904933	2 FHA	Fraser	2
PCC_0474		106, 8927 152 ST	SURREY	BC	V3R 4E5	CANADA	49.1651524	-122.8016852	2 FHA	Fraser	2
PCC_0475		8999 SCHOOL ST	CHILLIWACK	BC	V2P 4L5	CANADA	49.1655841	-121.9607173	2 FHA	Fraser	2
PCC_0476	PO BOX 2019	ROCKY MOUNTAIN HEALTH CENTRE, 901 5 AVE	FERNIE	BC	V0B 1M0	CANADA	49.508097	-115.0618739	1 IHA	Interior	3
PCC_0477	BOX 100	KOOTENAY MICRO PRACTICE, 300, 901 FRONT ST, MAIL	NELSON	BC	V1L 4C1	CANADA	49.4967906	-117.2904374	1 IHA	Interior	1
PCC_0478		4, 9025,160TH ST	SURREY	BC	V4N 2X7	CANADA	49.1669154	-122.7813251	2 FHA	Fraser	3
PCC_0479		GALIANO HEALTH CARE CENTRE, 908 BURRILL RD	GALIANO	BC	V0N 1P0	CANADA	48.8714644	-123.3206518	4 VIHA	Vancouver Island	2
PCC_0480		VILLAGE MEDICAL CLINIC, 9089 GLOVER RD	LANGLEY	BC	V1M 3S3	CANADA	49.112429	-122.6454178	2 FHA	Fraser	1
PCC_0481	PO BOX 130	VILLAGE MEDICAL CLINIC, 1, 9089 GLOVER RD	FORT LANGLEY	BC	V1M 2R5	CANADA	49.1676937	-122.5812094	2 FHA	Fraser	2
PCC_0482		BEAR CREEK MEDICAL CLINIC, 9093 KING GEORGE BLVD	SURREY	BC	V3V 5V7	CANADA	49.1677528	-122.8461524	2 FHA	Fraser	1
PCC_0483		BEAR CREEK MEDICAL CLINIC, 105, 9093 KING GEORGE BLVD	SURREY	BC	V3V 5V7	CANADA	49.1677528	-122.8461524	2 FHA	Fraser	6
PCC_0484		THE YOUNG CLINIC, 203, 9123 MARY ST	CHILLIWACK	BC	V2P 4H7	CANADA	49.1681573	-121.9610562	2 FHA	Fraser	6
PCC_0485	PO BOX 1619	9140 GRANVILLE ST	PORT HARDY	BC	V0N 2P0	CANADA	50.7210678	-127.5024595	4 VIHA	Vancouver Island	6
PCC_0486		KING EDWARD MEDICAL CENTRE, 920 KING EDWARD AVE W	VANCOUVER	BC	V5Z 2E2	CANADA	49.249086	-123.1257305	3 VCHA	Vancouver Coastal	1
PCC_0487		921A CANADA AVE	DUNCAN	BC	V9L 1V2	CANADA	48.7842866	-123.7088712	4 VIHA	Vancouver Island	2
PCC_0488		PANDORA CLINIC, 922 PANDORA ST	VICTORIA	BC	V8V 3P3	CANADA	48.4277813	-123.3578071	4 VIHA	Vancouver Island	5
PCC_0489		SCOTT CARE PHARMACY AND MEDICAL CLINIC, 202, 9278 SCOTT RD	SURREY	BC	V3V 4B8	CANADA	49.1716583	-122.8896584	2 FHA	Fraser	2
PCC_0490		CAPILANO MALL, 935 MARINE DR, CURE MEDICAL CLINIC	NORTH VANCOUVER	BC	V7P 1S3	CANADA	49.3214885	-123.1003144	3 VCHA	Vancouver Coastal	2
PCC_0491	PO BOX 8000	937 7 AVE	INVERMERE	BC	V0A 1K0	CANADA	50.5058308	-116.0284293	1 IHA	Interior	5
PCC_0492		AISHA MEDICAL CLINIC, 107, 9385 SCOTT RD	DELTA	BC	V4C 0B5	CANADA	49.1737148	-122.8906727	2 FHA	Fraser	2
PCC_0493		ELICARE MEDICAL CLINIC, 282 9600 CAMERON ST	BURNABY	BC	V3J 7N3	CANADA	49.2526712	-122.8981429	2 FHA	Fraser	3
PCC_0494		CAMERON MEDICAL CENTRE, 240, 9600 CAMERON ST	BURNABY	BC	V3J 7N3	CANADA	49.2526712	-122.8981429	2 FHA	Fraser	4
PCC_0495		G2, 9639 137A ST	SURREY	BC	V3T 0M1	CANADA	49.178002	-122.8423162	2 FHA	Fraser	4
PCC_0496		GRACE MEDICAL CLINIC, 9655 137 ST	SURREY	BC	V3T 4G8	CANADA	49.1780932	-122.8435855	2 FHA	Fraser	2
PCC_0497	BOX 59	CORTES COMMUNITY HEALTH CENTRE, 983 BEASLEY RD	MANSONS LANDING	BC	V0P 1K0	CANADA	50.0590026	-124.9792259	4 VIHA	Vancouver Island	1
PCC_0498		LIVEWELL MEDICAL CENTRE, 9839 138 ST	SURREY	BC	V3T 5E3	CANADA	49.1815689	-122.8398244	2 FHA	Fraser	2
PCC_0499		ABC MEDICAL CLINIC, 9842 101 AVE	FORT ST JOHN	BC	V1J 2B2	CANADA	56.2476385	-120.8452216	5 NHA	Northern	8
PCC_0500		LOUGHED MALL, 300, 9855 AUSTIN RD, VIVA CARE MEDICAL GROUP	BURNABY	BC	V3J 1N5	CANADA	49.2512273	-122.8958631	2 FHA	Fraser	2
PCC_0501		HIGHROADS MEDICAL CLINIC, 101, 9855 AUSTIN AVE	BURNABY	BC	V3J 1N4	CANADA	49.2512273	-122.8958631	2 FHA	Fraser	2
PCC_0502		CHEMAINUS MEDICAL CLINIC, 9892 ESPLANADE ST	CHEMAINUS	BC	V0R 1K1	CANADA	48.9262952	-123.7163167	4 VIHA	Vancouver Island	5
PCC_0503	BAG 999	WRINCH MEMORIAL DOCTORS CLINIC	HAZELTON	BC	V0J 1Y0	CANADA			5 NHA	Northern	2
PCC_0504	BOX 1090	UCLUELET MEDICAL CENTRE	UCLUELET	BC	V0R 3A0	CANADA			4 VIHA	Vancouver Island	1
PCC_0505	BOX 1878	BASTION MEDICAL CENTRE	REVELSTOKE	BC	V0E 2S0	CANADA			1 IHA	Interior	1
PCC_0506	BOX 211	OKANAGAN FALLS MEDICAL CLINIC	OKANAGAN FALLS	BC	V0H 1R0	CANADA			1 IHA	Interior	1
PCC_0507	BOX 369	RIVER CITY MEDICAL CENTRE, STATION A	CAMPBELL RIVER	BC	V9W 5B6	CANADA			4 VIHA	Vancouver Island	1
PCC_0508	BOX 730	SALMON ARM MEDICAL CLINIC, STN MAIN	SALMON ARM	BC	V1E 4N8	CANADA			1 IHA	Interior	1
PCC_0509	BOX 9200	DIAMOND HEAD MEDICAL CLINIC	SQUAMISH	BC	V8B 0C1	CANADA			3 VCHA	Vancouver Coastal	1
PCC_0510	BOX 998	THE MEDICAL CLINIC	MERRITT	BC	V1K 1B8	CANADA			1 IHA	Interior	1
PCC_0511	BOX, 11666	DR JENNIFER CHOI'S MAIL LAITY ST, DR JENNIFER CHOIS MAILBOX DOCTORS LOUNGE AT	MAPLE RIDGE	BC	V2X 5A3	CANADA			2 FHA	Fraser	1
PCC_0512	BOX, ROYAL C	DOCTOR'S MAIL COLUMBIA ST E	NEW WESTMINSTER	BC	V3L 3W7	CANADA			2 FHA	Fraser	1
PCC_0513	BOX, ROYAL J	DOCTOR'S MAIL BAY ST	VICTORIA	BC	V8R 1J8	CANADA			4 VIHA	Vancouver Island	1
PCC_0514		TLC MEDICAL CLINIC, 250 7155, KINGSWAY	BURNABY	BC	V5E 0A6	CANADA	49.8407852	-124.5231098	2 FHA	Fraser	1
PCC_0515		PRIMACY CLINIC, 8195 SCOTT RD	DELTA	BC	V4C 6P7	CANADA	49.1500573	-122.8915485	2 FHA	Fraser	1
PCC_0516		WESTGATE MEDICAL CLINIC, 402, 40395 LOUGHEED HIGHWAY	MAPLE RIDGE	BC	V2X 2P9	CANADA	49.1829614	-122.5531452	2 FHA	Fraser	1
PCC_0517	PO BOX 4316	RPO RICHMOND CENTRE	RICHMOND	BC	V6Y 3Y3	CANADA			3 VCHA	Vancouver Coastal	1
PCC_0518	PO BOX 10	TEXADA HEALTH CENTRE	GILLIES BAY	BC	V0N 1W0	CANADA			3 VCHA	Vancouver Coastal	1
PCC_0519	PO BOX 1149	FORT ST JAMES MEDICAL CLINIC	FORT ST JAMES	BC	V0J 1P0	CANADA			5 NHA	Northern	3
PCC_0520	PO BOX 1170	GOLDEN MEDICAL CLINIC	GOLDEN	BC	V0A 1H0	CANADA			1 IHA	Interior	5
PCC_0521	PO BOX 160	KIMBERLEY MEDICAL CLINIC, STN MAIN	KIMBERLEY	BC	V1A 2Y6	CANADA			1 IHA	Interior	6
PCC_0522	PO BOX 161	EAST SHORE COMMUNITY HEALTH CENTRE	CRAWFORD BAY	BC	V0B 1E0	CANADA			1 IHA	Interior	1
PCC_0523	PO BOX 1690	TUMBLER RIDGE COMMUNITY HEALTH CENTRE, COMMUNITY HEALTH CENTRE	TUMBLER RIDGE	BC	V0C 2W0	CANADA			5 NHA	Northern	3
PCC_0524	PO BOX 1720	VILLAGE MEDICAL CLINIC	100 MILE HOUSE	BC	V0K 2E0	CANADA			1 IHA	Interior	1
PCC_0525	PO BOX 188	GREENWOOD MEDICAL CLINIC	GREENWOOD	BC	V0H 1J0	CANADA			1 IHA	Interior	2
PCC_0526	PO BOX 2001	COMOX CENTER PO	COMOX	BC	V9M 4H3	CANADA			4 VIHA	Vancouver Island	1
PCC_0527	PO BOX 20017	SOOKE CENTER PO	SOOKE	BC	V9Z 1L6	CANADA			4 VIHA	Vancouver Island	1
PCC_0528	PO BOX 2019	ROCKY MOUNTAIN HEALTH CENTRE	FERNIE	BC	V0B 1M0	CANADA			1 IHA	Interior	3
PCC_0529	PO BOX 220	BELLA COOLA MEDICAL CLINIC	BELLA COOLA	BC	V0T 1C0	CANADA			3 VCHA	Vancouver Coastal	2
PCC_0530	PO BOX 2214	CAPRI CENTRE	KELOWNA	BC	V1Y 9N9	CANADA			1 IHA	Interior	1
PCC_0531	PO BOX 250	PARKSVILLE MEDICAL CLINIC	PARKSVILLE	BC	V9P 2G4	CANADA			4 VIHA	Vancouver Island	2
PCC_0532	PO BOX 250	HARBOURSIDE MEDICAL AND WALK IN CLINIC	QUATHIASKI COVE	BC	V0P 1N0	CANADA			4 VIHA	Vancouver Island	2
PCC_0533	PO BOX 250	COWRIE MEDICAL CLINIC	SECHELT	BC	V0N 3A0	CANADA			3 VCHA	Vancouver Coastal	1
PCC_0534	PO BOX 2769	CHISEL PEAK MEDICAL CLINIC	INVERMERE	BC	V0A 1K0	CANADA			1 IHA	Interior	2
PCC_0535	PO BOX 277	STN DEL CENTRE	MAPLE RIDGE	BC	V2X 7G2	CANADA			2 FHA	Fraser	1
PCC_0536	PO BOX 279	NK'MIP RESOURCE CENTRE 1165,SENPOKCHIN B	OLIVER	BC	V0H 1T0	CANADA			1 IHA	Interior	1
PCC_0537	PO BOX 2907	BROADWAY MEDICAL CLINIC, BROADWAY AVE	SMITHERS	BC	V0J 2N0	CANADA			5 NHA	Northern	1
PCC_0538	PO BOX 30035	REYNOLDS POST OFFICE RPO SAANICH CENTRE	VICTORIA	BC	V8X 5E1	CANADA			4 VIHA	Vancouver Island	1
PCC_0539	PO BOX 340	QUADRA ISLAND MEDICAL CLINIC	QUATHIASKI COVE	BC	V0P 1N0	CANADA			4 VIHA	Vancouver Island	1
PCC_0540	PO BOX 369	STATION A RIVER CITY MEDICAL CLINIC, STN A	CAMPBELL RIVER	BC	V9W 5B6	CANADA			4 VIHA	Vancouver Island	3
PCC_0541	PO BOX 39	LYTTON MEDICAL CLINIC	LYTTON	BC	V0K 1Z0	CANADA			1 IHA	Interior	1

**PCC_0542**	PO BOX 410	CHASE MEDICAL CENTRE	CHASE	BC	V0E 1M0	CANADA			1 IHA	Interior	2
**PCC_0543**	PO BOX 45058	WHITEFOOT MEDICAL CLINIC	KELOWNA	BC	V1P 1P3	CANADA			1 IHA	Interior	1
**PCC_0544**	PO BOX 489	MAIN ST MEDICAL ASSOCIATES	OLIVER	BC	V0H 1T0	CANADA			1 IHA	Interior	1
**PCC_0545**	PO BOX 49	NAMGIS CLINIC	ALERT BAY	BC	V0N 1A0	CANADA			4 VIHA	Vancouver Island	1
**PCC_0546**	PO BOX 500	STATION MAIN, SURREY NORTH COMMUNITY HEALTH CENTRE	SURREY	BC	V3T 5B7	CANADA			2 FHA	Fraser	1
**PCC_0547**	PO BOX 51032	RPO CREST CENTRE	BURNABY	BC	V3N 5B9	CANADA			2 FHA	Fraser	1
**PCC_0548**	PO BOX 51089	CREST CENTER PO	BURNABY	BC	V3N 2S7	CANADA			2 FHA	Fraser	1
**PCC_0549**	PO BOX 52510	COQUITLAM CENTRE	COQUITLAM	BC	V3B 5R0	CANADA			2 FHA	Fraser	1
**PCC_0550**	PO BOX 669	MCBRIDE MEDICAL CLINIC	MCBRIDE	BC	V0J 2E0	CANADA			5 NHA	Northern	1
**PCC_0551**	PO BOX 69	PEMBERTON MEDICAL CLINIC	PEMBERTON	BC	V0N 2L0	CANADA			3 VCHA	Vancouver Coastal	3
**PCC_0552**	PO BOX 700	MONASHEE MEDICAL CLINIC, MONASHEE CLINIC	ROSSLAND	BC	V0G 1Y0	CANADA			1 IHA	Interior	1
**PCC_0553**	PO BOX 730	SALMO WELLNESS CENTRE	SALMO	BC	V0G 1Z0	CANADA			1 IHA	Interior	2
**PCC_0554**	PO BOX 730	SALMON ARM MEDICAL CLINIC	SALMON ARM	BC	V1E 4N8	CANADA			1 IHA	Interior	10
**PCC_0555**	PO BOX 781	COLUMBIA COAST CLINIC, COLUMBIA COAST CLINIC 1371B CEDAR ST, STN A	CAMPBELL RIVER	BC	V9W 6Y4	CANADA			4 VIHA	Vancouver Island	1
**PCC_0556**	PO BOX 799	GENERAL PRACTICE	BARRIERE	BC	V0E 1E0	CANADA			1 IHA	Interior	1
**PCC_0557**	PO BOX 8	STEWART HEALTH CENTRE	STEWART	BC	V0T 1W0	CANADA			5 NHA	Northern	1
**PCC_0558**	PO BOX 8000	INVERMERE MEDICAL CLINIC	INVERMERE	BC	V0A 1K0	CANADA			1 IHA	Interior	1
**PCC_0559**	PO BOX 860	DESERT DOCTORS' CLINIC	OSOYOOS	BC	V0H 1V0	CANADA			1 IHA	Interior	2
**PCC_0560**	PO BOX 879	EXETER CLINIC	100 MILE HOUSE	BC	V0K 2E0	CANADA			1 IHA	Interior	3
**PCC_0561**	PO BOX 88568	NEWTON CENTRE	SURREY	BC	V3W 0X1	CANADA			2 FHA	Fraser	1
**PCC_0562**	PO BOX 9	NEW DENVER MEDICAL CLINIC	NEW DENVER	BC	V0G 1S0	CANADA			1 IHA	Interior	1
**PCC_0563**		CLAYTON MARKET MEDICAL CLINIC, UNIT 104B, 191161 FRASER HIGHWAY	SURREY	BC	V3S 8E7	CANADA	49.1500793	-122.7631934	2 FHA	Fraser	1
**UNM_0001**		600, 1001 BROADWAY W	VANCOUVER	BC	V6H 4B1	CANADA	49.2637133	-123.1271155	3 VCHA	Vancouver Coastal	2
**UNM_0002**		SUITE 111, 151, 10090 152 ST	SURREY	BC	V3R 8X8	CANADA	49.1857533	-122.8005828	2 FHA	Fraser	2
**UNM_0003**	PO BOX 590	SELKIRK MEDICAL GROUP, SUITE 201, 101 1 ST WEST	REVELSTOKE	BC	V0E 2S0	CANADA	50.9988139	-118.1956834	1 IHA	Interior	4
**UNM_0004**		201, 101 WEST 16 ST	NORTH VANCOUVER	BC	V7M 1T3	CANADA	49.3228636	-123.0728134	3 VCHA	Vancouver Coastal	4
**UNM_0005**		103, 1015 AUSTIN AVE	COQUITLAM	BC	V3K 3N9	CANADA	49.2493466	-122.8666498	2 FHA	Fraser	2
**UNM_0006**		AUSTIN FAMILY PHYSICIANS, 102, 1015 AUSTIN AVE	COQUITLAM	BC	V3K 3N9	CANADA	49.2493466	-122.8666498	2 FHA	Fraser	6
**UNM_0007**		101, 10151 NO 3	RICHMOND	BC	V7A 4R6	CANADA	49.1392707	-123.1378012	3 VCHA	Vancouver Coastal	3
**UNM_0008**		201, 10190 152A ST	SURREY	BC	V3R 1J7	CANADA	49.1877452	-122.7990876	2 FHA	Fraser	2
**UNM_0009**		101, 10277 154 ST	SURREY	BC	V3R 4J7	CANADA	49.1892799	-122.795854	2 FHA	Fraser	2
**UNM_0010**		201, 1077 56 ST	DELTA	BC	V4L 2A2	CANADA	49.0228099	-123.0686993	2 FHA	Fraser	5
**UNM_0011**		SPECTRUM HEALTH, 702, 1080 HOWE ST	VANCOUVER	BC	V6Z 2T1	CANADA	49.2788187	-123.125094	3 VCHA	Vancouver Coastal	8
**UNM_0012**		MANHAS MEDICAL, 101, 1106 AUSTIN AVE	COQUITLAM	BC	V3K 3P5	CANADA	49.2487042	-122.8615973	2 FHA	Fraser	3
**UNM_0013**		111 CORBETT RD	SALT SPRING ISLAND	BC	V8K 2R8	CANADA	48.8611808	-123.5080596	4 VIHA	Vancouver Island	2
**UNM_0014**		1111 SPRINGFIELD RD	KELOWNA	BC	V1Y 8R7	CANADA	49.8765889	-119.4763412	1 IHA	Interior	9
**UNM_0015**		1113 103 AVE	DAWSON CREEK	BC	V1G 2G8	CANADA	55.7582524	-120.2327642	5 NHA	Northern	2
**UNM_0016**		402, 1120 YATES ST	VICTORIA	BC	V8V 3M9	CANADA	48.4254202	-123.3524611	4 VIHA	Vancouver Island	2
**UNM_0017**		11291 BLUNDELL RD	RICHMOND	BC	V6Y 1L4	CANADA	49.1572862	-123.0986845	3 VCHA	Vancouver Coastal	2
**UNM_0018**		NORTH SHORE MEDICAL GROUP, 300, 1133 LONSDALE AVE	NORTH VANCOUVER	BC	V7M 2H4	CANADA	49.3185166	-123.0729368	3 VCHA	Vancouver Coastal	14
**UNM_0019**		WESTSIDE MEDICAL ASSOC, 1135 STEVENS RD	WEST KELOWNA	BC	V1Z 2S8	CANADA	49.8660094	-119.5634336	1 IHA	Interior	2
**UNM_0020**		305, 1160 BURRARD ST	VANCOUVER	BC	V6Z 2E8	CANADA	49.2792372	-123.1282976	3 VCHA	Vancouver Coastal	2
**UNM_0021**		306, 1160 BURRARD ST	VANCOUVER	BC	V6Z 2E8	CANADA	49.2792372	-123.1282976	3 VCHA	Vancouver Coastal	2
**UNM_0022**		BURRARD MEDICAL, 304, 1160 BURRARD ST	VANCOUVER	BC	V6Z 2E8	CANADA	49.2792372	-123.1282976	3 VCHA	Vancouver Coastal	2
**UNM_0023**		505, 1160 BURRARD ST	VANCOUVER	BC	V6Z 2E8	CANADA	49.2792372	-123.1282976	3 VCHA	Vancouver Coastal	3
**UNM_0024**		20, 1161 THE HIGH ST	COQUITLAM	BC	V3B 7W3	CANADA	49.2823566	-122.7955253	2 FHA	Fraser	2
**UNM_0025**		102, 1173 THE HIGH ST	COQUITLAM	BC	V3B 0B1	CANADA	49.2834959	-122.7953641	2 FHA	Fraser	2
**UNM_0026**		1173 THE HIGH ST	COQUITLAM	BC	V3B 0B1	CANADA	49.2831379	-122.7960487	2 FHA	Fraser	2
**UNM_0027**		326, 1175 COOK ST	VICTORIA	BC	V8V 4A1	CANADA	48.4239008	-123.3533691	4 VIHA	Vancouver Island	2
**UNM_0028**		107, 1175 COOK ST	VICTORIA	BC	V8V 4A1	CANADA	48.4239008	-123.3533691	4 VIHA	Vancouver Island	4
**UNM_0029**		201, 1190 HORNBY ST	VANCOUVER	BC	V6Z 2K5	CANADA	49.2784952	-123.1274915	3 VCHA	Vancouver Coastal	3
**UNM_0030**		11944 88 AVE	DELTA	BC	V4C 3C8	CANADA	49.163182	-122.891468	2 FHA	Fraser	7
**UNM_0031**		209, 1200 LYNN VALLEY RD	NORTH VANCOUVER	BC	V7J 2A2	CANADA	49.3360334	-123.0408014	3 VCHA	Vancouver Coastal	3
**UNM_0032**		201, 12005 238B ST	MAPLE RIDGE	BC	V4R 1W1	CANADA	49.2206034	-122.5617849	2 FHA	Fraser	2
**UNM_0033**		6, 1230 ALWARD ST	PRINCE GEORGE	BC	V2M 7B1	CANADA	53.912914	-122.7661766	5 NHA	Northern	2
**UNM_0034**		3, 1230 ALWARD ST	PRINCE GEORGE	BC	V2M 7B1	CANADA	53.912914	-122.7661766	5 NHA	Northern	2
**UNM_0035**		1230 ALWARD ST, PRINCE GEORGE, SUITE: 4	PRINCE GEORGE	BC	V2M 7B1	CANADA	53.912914	-122.7661766	5 NHA	Northern	3
**UNM_0036**		5, 1230 ALWARD ST	PRINCE GEORGE	BC	V2M 7B1	CANADA	53.912914	-122.7661766	5 NHA	Northern	4
**UNM_0037**		100, 12321 BEECHER ST	SURREY	BC	V4A 3A7	CANADA	49.0532438	-122.8804774	2 FHA	Fraser	3
**UNM_0038**	PO BOX 698	124 14 AVE N	CRESTON	BC	V0B 1G0	CANADA	49.0961022	-116.5093598	1 IHA	Interior	2
**UNM_0039**		114, 12578 72 AVE	SURREY	BC	V3W 2M6	CANADA	49.1332766	-122.8741661	2 FHA	Fraser	2
**UNM_0040**	PO BOX 308	1260 MAIN ST	SMITHERS	BC	V0J 2N0	CANADA	54.780659	-127.1688594	5 NHA	Northern	3
**UNM_0041**		101, 12827 76 AVE	SURREY	BC	V3W 1V3	CANADA	49.1417274	-122.8670066	2 FHA	Fraser	4
**UNM_0042**		1283 NANAIMO ST	VANCOUVER	BC	V5L 4T5	CANADA	49.2732641	-123.0570225	3 VCHA	Vancouver Coastal	2
**UNM_0043**	PO BOX 45085	12851 16 AVE	SURREY	BC	V4A 9L1	CANADA	49.0315074	-122.8659358	2 FHA	Fraser	2
**UNM_0044**		1295 W BROADWAY	VANCOUVER	BC	V6H 1G7	CANADA	49.2635749	-123.1335093	3 VCHA	Vancouver Coastal	2
**UNM_0045**	PO BOX 1620	13214 KELLY AVE	SUMMERLAND	BC	V0H 1Z0	CANADA	49.6022788	-119.6812158	1 IHA	Interior	5
**UNM_0046**		200, 1330 8 AVE W	VANCOUVER	BC	V6H 4A6	CANADA	49.2643048	-123.1344561	3 VCHA	Vancouver Coastal	2
**UNM_0047**		CENTRAL CITY OFFICE, 13450 102 AVE SUITE 400	SURREY	BC	V3T 0H1	CANADA	49.1878023	-122.8493047	2 FHA	Fraser	4
**UNM_0048**		502, 13737 96 AVE	SURREY	BC	V3V 0C6	CANADA	49.1774643	-122.842723	2 FHA	Fraser	2
**UNM_0049**		13737 96 AVE, SUITE 103	SURREY	BC	V3V 0C6	CANADA	49.1774643	-122.842723	2 FHA	Fraser	5
**UNM_0050**		101, 13771 72A AVE	SURREY	BC	V3W 9C6	CANADA	49.1349362	-122.8393749	2 FHA	Fraser	2
**UNM_0051**		4, 13791 72 AVE	SURREY	BC	V3W 9Y9	CANADA	49.1342693	-122.8390639	2 FHA	Fraser	3
**UNM_0052**		335, 138 E13 ST	NORTH VANCOUVER	BC	V7L OE5	CANADA	49.3204014	-123.0710295			2
**UNM_0053**		310, 138 13 ST E	NORTH VANCOUVER	BC	V7L 0E5	CANADA	49.3204014	-123.0710295	3 VCHA	Vancouver Coastal	2
**UNM_0054**		QUINSAM MEDICAL GROUP, 280, 1400 DOGWOOD ST	CAMPBELL RIVER	BC	V9W 3A6	CANADA	50.0294981	-125.2509917	4 VIHA	Vancouver Island	10
**UNM_0055**		1406 COLEMAN ST	NORTH VANCOUVER	BC	V7K 1W6	CANADA	49.3510278	-123.0348189	3 VCHA	Vancouver Coastal	2
**UNM_0056**		1408 8 AVE W, SUITE 400	VANCOUVER	BC	V6H 1E1	CANADA	49.2642284	-123.1363291	3 VCHA	Vancouver Coastal	2
**UNM_0057**		106, 14199 62 AVE	SURREY	BC	V3X 0B1	CANADA	49.1157303	-122.829448	2 FHA	Fraser	3
**UNM_0058**		201, 143 FOURTH AVE S	WILLIAMS LAKE	BC	V2G 1J8	CANADA	52.1263498	-122.1412404	1 IHA	Interior	2
**UNM_0059**		SUITE 305, 1434 IRONWOOD ST	CAMPBELL RIVER	BC	V9W 5T5	CANADA	50.0299483	-125.2549816	4 VIHA	Vancouver Island	2
**UNM_0060**		203, 1448 COMMERCIAL DR	VANCOUVER	BC	V5L 3X9	CANADA	49.2715949	-123.0691652	3 VCHA	Vancouver Coastal	4
**UNM_0061**		103, 149 INGRAM ST	DUNCAN	BC	V9L 1N8	CANADA	48.7794707	-123.7091381	4 VIHA	Vancouver Island	10
**UNM_0062**		205, 1497 ADMIRALS RD	VICTORIA	BC	V9A 7K2	CANADA	48.4475395	-123.4264501	4 VIHA	Vancouver Island	12
**UNM_0063**		TAMARACK MEDICAL GROUP, 191, 1500 CRANBROOK ST N	CRANBROOK	BC	V1C 3S8	CANADA	49.5245223	-115.7504569	1 IHA	Interior	5
**UNM_0064**		102, 15122 72 AVE	SURREY	BC	V3S 2G2	CANADA	49.1335165	-122.8026133	2 FHA	Fraser	3
**UNM_0065**		1516 FAIRVIEW RD	PENTICTON	BC	V2A 6A3	CANADA	49.4819532	-119.5930404	1 IHA	Interior	2
**UNM_0066**		301A, 15252 32 AVE	SURREY	BC	V3S 0R7	CANADA	49.0610132	-122.7984631	2 FHA	Fraser	2
**UNM_0067**		37, 15300 105 AVE	SURREY	BC	V3R 6A7	CANADA	49.1931375	-122.7977145	2 FHA	Fraser	2
**UNM_0068**		28, 15300 105 AVE	SURREY	BC	V3R 6A7	CANADA	49.1931375	-122.7977145	2 FHA	Fraser	2
**UNM_0069**		15331 16 AVE	SURREY	BC	V4A 0C1	CANADA	49.0313142	-122.798629	2 FHA	Fraser	3
**UNM_0070**		1555 WINDERMERE LOOP RD	WINDERMERE	BC	V0B 2L1	CANADA	50.4804182	-115.9601438	1 IHA	Interior	2
**UNM_0071**		101, 156 MORISON AVE	PARKSVILLE	BC	V9P 2G4	CANADA	49.3209432	-124.3150098	4 VIHA	Vancouver Island	2
**UNM_0072**		230, 1590 CEDAR HILL CROSS RD	VICTORIA	BC	V8P 2P5	CANADA	48.4612336	-123.3320773	4 VIHA	Vancouver Island	2
**UNM_0073**		SUITE 309, 16088 84 AVE	SURREY	BC	V4N 0V9	CANADA	49.1551695	-122.7763394	2 FHA	Fraser	5
**UNM_0074**		388, 1641 HILLSIDE AVE	VICTORIA	BC	V8T 5G1	CANADA	48.4450083	-123.3337894	4 VIHA	Vancouver Island	2
**UNM_0075**		103, 1660 FELTHAM RD	VICTORIA	BC	V8N 2A1	CANADA	48.4764944	-123.3336197	4 VIHA	Vancouver Island	4
**UNM_0076**		600, 1665 BROADWAY W, PHYSICIAN HEALTH PROGRAM	VANCOUVER	BC	V6J 1X1	CANADA	49.2639565	-123.1427202	3 VCHA	Vancouver Coastal	3
**UNM_0077**		515, 1669 VICTORIA ST	PRINCE GEORGE	BC	V2L 2L5	CANADA	53.9085796	-122.7554255	5 NHA	Northern	2
**UNM_0078**		513, 1669 VICTORIA ST	PRINCE GEORGE	BC	V2L 2L5	CANADA	53.9085796	-122.7554255	5 NHA	Northern	2
**UNM_0079**		207, 1669 VICTORIA ST	PRINCE GEORGE	BC	V2L 2L5	CANADA	53.9085796	-122.7554255	5 NHA	Northern	2
**UNM_0080**		311, 1669 VICTORIA ST	PRINCE GEORGE	BC	V2L 2L5	CANADA	53.9085796	-122.7554255	5 NHA	Northern	2
**UNM_0081**		VICTORIA MEDICAL BLDG, 212, 1669 VICTORIA ST	PRINCE GEORGE	BC	V2L 2L5	CANADA	53.9085796	-122.7554255	5 NHA	Northern	2
**UNM_0082**		212, 168 EAST13 ST	NORTH VANCOUVER	BC	V7L 4W8	CANADA	49.3202668	-123.0697176	3 VCHA	Vancouver Coastal	2

UNM_0083		302, 1711 COOK ST	VICTORIA	BC	V8T 3P2	CANADA	48.4288185	-123.3527566	4 VIHA	Vancouver Island	2
UNM_0084		304, 1711 COOK ST	VICTORIA	BC	V8T 3P2	CANADA	48.4288185	-123.3527566	4 VIHA	Vancouver Island	2
UNM_0085		203, 1711 COOK ST	VICTORIA	BC	V8T 3P2	CANADA	48.4288185	-123.3527566	4 VIHA	Vancouver Island	3
UNM_0086		301, 1711 COOK ST	VICTORIA	BC	V8T 3P2	CANADA	48.4288185	-123.3527566	4 VIHA	Vancouver Island	4
UNM_0087		406, 1750 10 AVE E	VANCOUVER	BC	V5N 5K4	CANADA	49.2610979	-123.0683859	3 VCHA	Vancouver Coastal	2
UNM_0088		202, 1757 BEAUFORT AVE	COMOX	BC	V9M 1R8	CANADA	49.6718576	-124.9255551	4 VIHA	Vancouver Island	3
UNM_0089		1757 ROBSON ST	VANCOUVER	BC	V6G 1C9	CANADA	49.2911436	-123.1348772	3 VCHA	Vancouver Coastal	3
UNM_0090	PO BOX 1540	202, 180 MCCARTER ST	PARKSVILLE	BC	V9P 2H4	CANADA	49.3187511	-124.3095221	4 VIHA	Vancouver Island	2
UNM_0091		204, 1800 TRANQUILLE RD	KAMLOOPS	BC	V2B 3L9	CANADA	50.7000431	-120.3945679	1 IHA	Interior	3
UNM_0092		202, 1824 GORDON DR	KELOWNA	BC	V1Y 0E2	CANADA	49.8814892	-119.4774988	1 IHA	Interior	3
UNM_0093		2, 1836 COMOX AVE	COMOX	BC	V9M 3M7	CANADA	49.6743515	-124.9285162	4 VIHA	Vancouver Island	2
UNM_0094	PO BOX 5095	1845 FORT ST STN B	VICTORIA	BC	V8R 6N3	CANADA	48.4294848	-123.3293751	4 VIHA	Vancouver Island	2
UNM_0095		360, 1855 KIRSCHNER RD	KELOWNA	BC	V1Y 4N7	CANADA	49.8797352	-119.457534	1 IHA	Interior	2
UNM_0096		1872 KINGSWAY	VANCOUVER	BC	V5N 2S7	CANADA	49.2457108	-123.0669959	3 VCHA	Vancouver Coastal	2
UNM_0097		MD ESTHETICS, 201, 1910 SOOKE RD	VICTORIA	BC	V9B 1V7	CANADA	48.441496	-123.4707503	4 VIHA	Vancouver Island	2
UNM_0098		104B 19161 FRASER HWY	SURREY	BC	V3S 8E7	CANADA	49.1196133	-122.6918167	2 FHA	Fraser	3
UNM_0099		204, 1931 MOUNT NEWTON CROSS RD	SAANICHTON	BC	V8M 2A9	CANADA	48.5943454	-123.4191629	4 VIHA	Vancouver Island	2
UNM_0100		219, 1940 LONSDALE AVE	NORTH VANCOUVER	BC	V7M 2K2	CANADA	49.3267424	-123.0717016	3 VCHA	Vancouver Coastal	2
UNM_0101		306 1964 FORT ST	VICTORIA	BC	V8R 6R3	CANADA	48.4325195	-123.3234819	4 VIHA	Vancouver Island	2
UNM_0102		212, 1964 FORT ST	VICTORIA	BC	V8R 6R3	CANADA	48.4325195	-123.3234819	4 VIHA	Vancouver Island	2
UNM_0103		101, 1978 CLIFFE AVE	COURTENAY	BC	V9N 2L1	CANADA	49.6820275	-124.9861867	4 VIHA	Vancouver Island	2
UNM_0104		PORTLAND HOTEL SOCIETY, 20 HASTINGS ST W	VANCOUVER	BC	V6B 1G6	CANADA	49.2813928	-123.1054742	3 VCHA	Vancouver Coastal	2
UNM_0105		D114, 20159 88 AVE	LANGLEY	BC	V1M 0A4	CANADA	49.1649752	-122.6646057	2 FHA	Fraser	4
UNM_0106		2071 GLENMOHR DR	KAMLOOPS	BC	V1S 1N4	CANADA	50.6458514	-120.3797534	1 IHA	Interior	2
UNM_0107	PO BOX 279	2087 GRANITE AVE	MERRITT	BC	V1K 1B8	CANADA	50.1107497	-120.7870126	1 IHA	Interior	2
UNM_0108		ASPEN MEDICAL, 101B, 2099, 152 ST	SURREY	BC	V4A 4N7	CANADA	49.0398873	-122.8014317	2 FHA	Fraser	2
UNM_0109	PO BOX 1479	211 PARK ST	HOPE	BC	V0X 1L0	CANADA	49.3803795	-121.4452819	2 FHA	Fraser	2
UNM_0110	PO BOX 247	2135 NORRIS AVE	LUMBY	BC	V0E 2G0	CANADA	50.2493668	-118.9619637	1 IHA	Interior	2
UNM_0111		405, 2155 10 AVE	PRINCE GEORGE	BC	V2M 5J6	CANADA	53.9152456	-122.765827	5 NHA	Northern	2
UNM_0112		307, 2155 TENTH AVE	PRINCE GEORGE	BC	V2M 5J6	CANADA	53.9152456	-122.765827	5 NHA	Northern	3
UNM_0113		106, 2155 10TH AVE	PRINCE GEORGE	BC	V2M 5J6	CANADA	53.9152456	-122.765827	5 NHA	Northern	8
UNM_0114		228, 2155 ALLISON RD	VANCOUVER	BC	V6T 1T5	CANADA	49.2660996	-123.2427655	3 VCHA	Vancouver Coastal	9
UNM_0115		103, 2180 GLADWIN RD	ABBOTSFORD	BC	V2S 0H4	CANADA	49.0413244	-122.3149854	2 FHA	Fraser	3
UNM_0116		2185 WILSON AVE	PORT COQUITLAM	BC	V3C 6C1	CANADA	49.2611675	-122.7770801	2 FHA	Fraser	4
UNM_0117		212, 2187 OAK BAY AVE	VICTORIA	BC	V8R 1G1	CANADA	48.4263689	-123.3163548	4 VIHA	Vancouver Island	2
UNM_0118		3, 219 FERN RD	QUALICUM BEACH	BC	V9K 2M2	CANADA	49.3464455	-124.4480103	4 VIHA	Vancouver Island	2
UNM_0119		101, 2210 MAIN ST	PENTICTON	BC	V2A 5H8	CANADA	49.4738742	-119.5800415	1 IHA	Interior	4
UNM_0120		302 22112 52 AVE	LANGLEY	BC	V2Y 2M6	CANADA	49.0963994	-122.6114208	2 FHA	Fraser	2
UNM_0121		B, 22195 DEWDNEY TRUNK RD	MAPLE RIDGE	BC	V2X 3H7	CANADA	49.2208051	-122.6073939	2 FHA	Fraser	6
UNM_0122		201, 22242 48 AVE	LANGLEY	BC	V3A 3N5	CANADA	49.0889955	-122.6075649	2 FHA	Fraser	2
UNM_0123		230 ROBSON RD W	KELOWNA	BC	V1X 3C8	CANADA	49.887343	-119.3881672	1 IHA	Interior	3
UNM_0124	PO BOX 19094	RPO FOURTH AVE, 2302 4 AVE W	VANCOUVER	BC	V6K 4R8	CANADA	49.2679563	-123.1577515	3 VCHA	Vancouver Coastal	2
UNM_0125		225, 2306 HIGHWAY 6	VERNON	BC	V1T 7E3	CANADA	50.2563341	-119.2644204	1 IHA	Interior	2
UNM_0126	PO BOX 99	201, 23148 96 AVE	FORT LANGLEY	BC	V1M 2R4	CANADA	49.1687943	-122.5818142	2 FHA	Fraser	2
UNM_0127		234 HASTINGS ST E	VANCOUVER	BC	V6A 1P1	CANADA	49.2812741	-123.0987392	3 VCHA	Vancouver Coastal	4
UNM_0128		2349 MILLSTREAM RD	VICTORIA	BC	V9B 3R5	CANADA	48.4641647	-123.4992024	4 VIHA	Vancouver Island	2
UNM_0129		235 MARINE DR SE	VANCOUVER	BC	V5X 2S4	CANADA	49.2109845	-123.1013685	3 VCHA	Vancouver Coastal	4
UNM_0130		202, 236 GEORGIA ST E	VANCOUVER	BC	V6A 1Z7	CANADA	49.2782969	-123.0985215	3 VCHA	Vancouver Coastal	2
UNM_0131		105, 2362 WHATCOM RD	ABBOTSFORD	BC	V3G 0C1	CANADA	49.0451839	-122.2234927	2 FHA	Fraser	8
UNM_0132		1, 2379 BEVAN AVE	SIDNEY	BC	V8L 4M9	CANADA	48.6473855	-123.4008276	4 VIHA	Vancouver Island	2
UNM_0133		108, 2419 BELLEVUE AVE	WEST VANCOUVER	BC	V7V 4T4	CANADA	49.3335883	-123.1797683	3 VCHA	Vancouver Coastal	3
UNM_0134		PHARMASAVE WEST KELOWNA, 9A, 2484 MAIN ST	WEST KELOWNA	BC	V4T 2G2	CANADA	49.8305126	-119.6303314	1 IHA	Interior	2
UNM_0135		ROCKY POINT MEDICAL 2523 ST JOHNS ST	PORT MOODY	BC	V3H 2B3	CANADA	49.2763653	-122.8549287	2 FHA	Fraser	3
UNM_0136		2, 2629 CEDAR PARK PL	ABBOTSFORD	BC	V2T 3S4	CANADA	49.0499479	-122.3356748	2 FHA	Fraser	2
UNM_0137		2667 BROADWAY W	VANCOUVER	BC	V6K 2G2	CANADA	49.2642879	-123.1655319	3 VCHA	Vancouver Coastal	2
UNM_0138		203, 2689 PANDOSY ST	KELOWNA	BC	V1Y 9V9	CANADA	49.8665047	-119.4909109	1 IHA	Interior	2
UNM_0139		350, 2755 LOUGHEED HWY	PORT COQUITLAM	BC	V3B 5Y9	CANADA	49.2735893	-122.7891082	2 FHA	Fraser	3
UNM_0140		2755 TUTT ST APT 303	KELOWNA	BC	V1Y 0G1	CANADA	49.86577	-119.4894933	1 IHA	Interior	2
UNM_0141		PRIME MEDICAL, 2755 TUTT ST KELOWNA, BC V1Y 0G1, 201	KELOWNA	BC	V1Y 0G1	CANADA	49.86577	-119.4894933	1 IHA	Interior	3
UNM_0142	PO BOX 74685	2768 BROADWAY W	VANCOUVER	BC	V6K 4P4	CANADA	49.2640115	-123.1675927	3 VCHA	Vancouver Coastal	2
UNM_0143		205, 2786 16 AVE W	VANCOUVER	BC	V6K 4M1	CANADA	49.2573174	-123.1672901	3 VCHA	Vancouver Coastal	3
UNM_0144		2817 DUFFERIN AVE	VICTORIA	BC	V8R 3L6	CANADA	48.4419315	-123.3116111	4 VIHA	Vancouver Island	2
UNM_0145		201, 284 HELMCKEN RD	VICTORIA	BC	V9B 1T2	CANADA	48.4555464	-123.4411345	4 VIHA	Vancouver Island	3
UNM_0146		284 HELMCKEN RD APT 101	VICTORIA	BC	V9B 1T2	CANADA	48.4555464	-123.4411345	4 VIHA	Vancouver Island	3
UNM_0147		108, 284 HELMCKEN RD	VICTORIA	BC	V9B 1T2	CANADA	48.4555464	-123.4411345	4 VIHA	Vancouver Island	3
UNM_0148		109, 284 HELMCKEN RD	VICTORIA	BC	V9B 1T2	CANADA	48.4555464	-123.4411345	4 VIHA	Vancouver Island	3
UNM_0149		102, 2845 CRUICKSHANK ST	ABBOTSFORD	BC	V2T 6X1	CANADA	49.0539702	-122.3246446	2 FHA	Fraser	3
UNM_0150		304, 2845 CRUICKSHANK ST	ABBOTSFORD	BC	V2T 6X1	CANADA	49.0539702	-122.3246446	2 FHA	Fraser	3
UNM_0151		DR LOUIS BOUCHER MEDICAL SERVICES INC, 204, 2903 32 AVE	VERNON	BC	V1T 2L6	CANADA	50.2655568	-119.2699663	1 IHA	Interior	2
UNM_0152		100, 2917 28 AVE	VERNON	BC	V1T 8L1	CANADA	50.2624531	-119.2704423	1 IHA	Interior	3
UNM_0153		118, 10151 3	RICHMOND	BC	V7A 4R6	CANADA	49.2165206	-122.8939474	3 VCHA	Vancouver Coastal	2
UNM_0154		142, 3000 LOUGHEED HWY	COQUITLAM	BC	V3B 1C5	CANADA	49.2736099	-122.793436	2 FHA	Fraser	3
UNM_0155		MEDICALWEST, 3001 GORDON AVE SUITE 212	COQUITLAM	BC	V3C 2K7	CANADA	49.2716022	-122.792874	2 FHA	Fraser	7
UNM_0156		101, 3002 32 AVE	VERNON	BC	V1T 2L7	CANADA	50.2651377	-119.2713816	1 IHA	Interior	3
UNM_0157		202, 3005 30 ST	VERNON	BC	V1T 9J5	CANADA	50.264402	-119.2706674	1 IHA	Interior	5
UNM_0158		303, 301 COLUMBIA E	NEW WESTMINSTER	BC	V3L 3W5	CANADA	49.2260098	-122.8933071	2 FHA	Fraser	2
UNM_0159		207, 301 COLUMBIA ST E	NEW WESTMINSTER	BC	V3L 3W5	CANADA	49.2260098	-122.8933071	2 FHA	Fraser	3
UNM_0160		SOPA SQUARE, 206, 3030 PANDOSY ST	KELOWNA	BC	V1Y 1W2	CANADA	49.8630374	-119.4918242	1 IHA	Interior	2
UNM_0161		480, 3033 IMMEL ST	ABBOTSFORD	BC	V2S 6S2	CANADA	49.0571105	-122.2732175	2 FHA	Fraser	3
UNM_0162		102, 3040 TUTT ST	KELOWNA	BC	V1Y 2H5	CANADA	49.8626485	-119.490148	1 IHA	Interior	2
UNM_0163	PO BOX 75555	EDGEMONT PO, 3050 EDGEMONT BLVD	NORTH VANCOUVER	BC	V7R 2N4	CANADA	49.3371401	-123.1009168	3 VCHA	Vancouver Coastal	2
UNM_0164		235, 3066 SHELBOURNE ST	VICTORIA	BC	V8R 6T9	CANADA	48.4450083	-123.3337894	4 VIHA	Vancouver Island	2
UNM_0165		325, 3066 SHELBOURNE ST	VICTORIA	BC	V8R 6T9	CANADA	48.4450083	-123.3337894	4 VIHA	Vancouver Island	3
UNM_0166		133, 3066 SHELBOURNE ST	VICTORIA	BC	V8R 6T9	CANADA	48.4450083	-123.3337894	4 VIHA	Vancouver Island	4
UNM_0167		300, 307 BROADWAY W	VANCOUVER	BC	V5Y 1P8	CANADA	49.2633249	-123.1112175	3 VCHA	Vancouver Coastal	2
UNM_0168		3167 MOUNTAIN HWY	NORTH VANCOUVER	BC	V7K 2H4	CANADA	49.3378063	-123.0391056	3 VCHA	Vancouver Coastal	2
UNM_0169		3185 KINGSWAY	VANCOUVER	BC	V5R 5K2	CANADA	49.2353846	-123.0378818	3 VCHA	Vancouver Coastal	3
UNM_0170		THE MEDICINE CABINET, 3188 BARONS RD SUITE 2	NANAIMO	BC	V9T 4B5	CANADA	49.2078315	-124.0016602	4 VIHA	Vancouver Island	2
UNM_0171		115, 3195 GRANVILLE ST	VANCOUVER	BC	V6H 3K2	CANADA	49.2572074	-123.1392212	3 VCHA	Vancouver Coastal	7
UNM_0172	PO BOX 32	302, 320 ALEXANDER ST NE	SALMON ARM	BC	V1E 4N2	CANADA	50.7019232	-119.2824431	1 IHA	Interior	3
UNM_0173		200, 321 NICOLA ST	KAMLOOPS	BC	V2C 6G6	CANADA	50.6720422	-120.3339644	1 IHA	Interior	2
UNM_0174		301, 321 NICOLA ST	KAMLOOPS	BC	V2C 6G6	CANADA	50.6720422	-120.3339644	1 IHA	Interior	2
UNM_0175		PREVENTUM: PERSONALIZED HEALTH CARE, 3210 DUNBAR ST	VANCOUVER	BC	V6S 2B7	CANADA	49.2577437	-123.1850331	3 VCHA	Vancouver Coastal	2
UNM_0176		110, 32156 HILLCREST AVE	ABBOTSFORD	BC	V2T 1S4	CANADA	49.0476582	-122.3330365	2 FHA	Fraser	2
UNM_0177		32243 HURD ST	MISSION	BC	V2V 3J9	CANADA	49.1342436	-122.3315152	2 FHA	Fraser	12
UNM_0178		101, 323 LOWER GANGES RD	SALT SPRING ISLAND	BC	V8K 2V4	CANADA	48.8598826	-123.5080333	4 VIHA	Vancouver Island	2
UNM_0179		3238 KING GEORGE BLVD	SURREY	BC	V4P 1A2	CANADA	49.0609444	-122.8119022	2 FHA	Fraser	2
UNM_0180		3, 3238 KING GEORGE BLVD	SURREY	BC	V4P 1A5	CANADA	49.0609444	-122.8119022	2 FHA	Fraser	3
UNM_0181		THE MARK MEDICAL, 104, 32625 SOUTH FRASER WAY	ABBOTSFORD	BC	V2T 1X8	CANADA	49.0518521	-122.3204469	2 FHA	Fraser	7
UNM_0182		23C, 3300 SMITH DR	ARMSTRONG	BC	V0E 1B1	CANADA	50.447125	-119.1835349	1 IHA	Interior	5
UNM_0183		3317 WESBROOK MALL	VANCOUVER	BC	V6S 0B1	CANADA	49.254767	-123.2355159	3 VCHA	Vancouver Coastal	2
UNM_0184		103, 3325 KINGSWAY	VANCOUVER	BC	V5R 5K6	CANADA	49.2339956	-123.0348675	3 VCHA	Vancouver Coastal	2
UNM_0185		1005, 333 BROOKSBANK AVE	NORTH VANCOUVER	BC	V7J 3S8	CANADA	49.3090528	-123.0419468	3 VCHA	Vancouver Coastal	2
UNM_0186		3407 32 AVE	VERNON	BC	V1T 2M9	CANADA	50.2654693	-119.2779982	1 IHA	Interior	3

**UNM_0187**		3438 MAIN ST	VANCOUVER	BC	V5V 3N2	CANADA	49.2543315	-123.0999926	3 VCHA	Vancouver Coastal	2
**UNM_0188**		301, 3491 SAANICH RD	VICTORIA	BC	V8X 1W8	CANADA	48.4544132	-123.3730997	4 VIHA	Vancouver Island	3
**UNM_0189**		VINTAGE VIEW MEDICAL, 107, 3500 CARRINGTON RD	WESTBANK	BC	V4T 3C1	CANADA	49.837993	-119.6095438	1 IHA	Interior	4
**UNM_0190**		3525 4 AVE W	VANCOUVER	BC	V5R 1N9	CANADA	49.2688325	-123.1822355	3 VCHA	Vancouver Coastal	2
**UNM_0191**		REBALANCE MD, 104, 3551 BLANSHARD ST	VICTORIA	BC	V8Z 0B9	CANADA	48.4556711	-123.3750955	4 VIHA	Vancouver Island	2
**UNM_0192**		3591 31ST ST	VERNON	BC	V1T 5J4	CANADA	50.2680782	-119.2719305	1 IHA	Interior	4
**UNM_0193**		390, 3600 LYSANDER LANE	RICHMOND	BC	V7B 1C3	CANADA	49.1895966	-123.1416692	3 VCHA	Vancouver Coastal	5
**UNM_0194**		SUITE 101, 3607 31ST ST	VERNON	BC	V1T 5J4	CANADA	50.2686619	-119.2717309	1 IHA	Interior	2
**UNM_0195**		WESTVIEW MEDICAL, 104, 3670 TOWNLINE RD	ABBOTSFORD	BC	V2T 0H2	CANADA	49.0691735	-122.3589093	2 FHA	Fraser	4
**UNM_0196**		2270, 3700 NO 3	RICHMOND	BC	V6X 3X2	CANADA	49.1863097	-123.1339168	3 VCHA	Vancouver Coastal	2
**UNM_0197**		BCIT STUDENT HEALTH CLINIC, 3700 WILLINGDON AVE, SE16, 127	BURNABY	BC	V5G 3H2	CANADA	49.247296	-123.001139	2 FHA	Fraser	3
**UNM_0198**		377 2 AVE E	VANCOUVER	BC	V5T 1B9	CANADA	49.2672098	-123.0965496	3 VCHA	Vancouver Coastal	3
**UNM_0199**		201, 37989 CLEVELAND AVE	SQUAMISH	BC	V8B 0R2	CANADA	49.6983179	-123.1540768	3 VCHA	Vancouver Coastal	2
**UNM_0200**		SUITE 202, 3811 CHATHAM ST	RICHMOND	BC	V7E 2Z4	CANADA	49.1267089	-123.1830756	3 VCHA	Vancouver Coastal	3
**UNM_0201**		STEVESTON MEDICAL GROUP, 204, 3811 CHATHAM ST	RICHMOND	BC	V7E 2Z4	CANADA	49.1267089	-123.1830756	3 VCHA	Vancouver Coastal	7
**UNM_0202**		207, 3825 SUNSET ST	BURNABY	BC	V5G 1T4	CANADA	49.2499871	-123.01893	2 FHA	Fraser	2
**UNM_0203**		203, 3825 SUNSET ST	BURNABY	BC	V5G 1T4	CANADA	49.2499871	-123.01893	2 FHA	Fraser	8
**UNM_0204**		207 3930 SHELBOURNE ST	VICTORIA	BC	V8P 5P6	CANADA	48.4672493	-123.3331826	4 VIHA	Vancouver Island	2
**UNM_0205**		106, 3960 QUADRA ST	VICTORIA	BC	V8X 4A3	CANADA	48.4680964	-123.3641552	4 VIHA	Vancouver Island	2
**UNM_0206**		104, 3965 KINGSWAY	BURNABY	BC	V5H 1Y8	CANADA	49.2330893	-123.0167063	2 FHA	Fraser	4
**UNM_0207**	PO BOX 400	400 TRUMPETER DR	GOLD RIVER	BC	V0P 1G0	CANADA	49.7792895	-126.0519894	4 VIHA	Vancouver Island	2
**UNM_0208**		4041 200B ST	LANGLEY	BC	V3A 1P1	CANADA	49.0754355	-122.6681705	2 FHA	Fraser	3
**UNM_0209**		4186 DUNBAR ST	VANCOUVER	BC	V6S 2E7	CANADA	49.2492829	-123.1848371	3 VCHA	Vancouver Coastal	4
**UNM_0210**		430 5 AVE	KAMLOOPS	BC	V2C 5Y2	CANADA	50.6736931	-120.3297507	1 IHA	Interior	2
**UNM_0211**		4313 GALLANT AVE	NORTH VANCOUVER	BC	V7G 2C1	CANADA	49.3265114	-122.9513122	3 VCHA	Vancouver Coastal	3
**UNM_0212**		4349 HASTINGS ST E	BURNABY	BC	V5C 2J8	CANADA	49.2812944	-123.0070629	2 FHA	Fraser	4
**UNM_0213**		201, 4380 LORIMER RD	WHISTLER	BC	V0N 1B4	CANADA	50.1198186	-122.9551699	3 VCHA	Vancouver Coastal	4
**UNM_0214**		4419 HASTINGS ST	BURNABY	BC	V5C 2K1	CANADA	49.2810825	-123.0050411	2 FHA	Fraser	3
**UNM_0215**		304, 4420 CHATTERTON WAY	VICTORIA	BC	V8X 5J2	CANADA	48.4874856	-123.3831491	4 VIHA	Vancouver Island	6
**UNM_0216**		4425 GERTRUDE ST	PORT ALBERNI	BC	V9Y 6J7	CANADA	49.2566482	-124.8087649	4 VIHA	Vancouver Island	2
**UNM_0217**		DR GEORGE S URBAN INC, 4460 CHATTERTON WAY, SUITE 220	VICTORIA	BC	V8X 5J2	CANADA	48.4904288	-123.3843912	4 VIHA	Vancouver Island	5
**UNM_0218**		1612, 4500 KINGSWAY AVE	BURNABY	BC	V5H 2A9	CANADA	49.2291216	-123.0048665	2 FHA	Fraser	2
**UNM_0219**		206, 4515 HARVEST DR	DELTA	BC	V4K 4L1	CANADA	49.0850087	-123.0647032	2 FHA	Fraser	2
**UNM_0220**		105, 4515 HARVEST DR	DELTA	BC	V4K 4L1	CANADA	49.0850087	-123.0647032	2 FHA	Fraser	5
**UNM_0221**		4539 MARINE AVE	POWELL RIVER	BC	V8A 2K5	CANADA	49.8387389	-124.5280618	3 VCHA	Vancouver Coastal	3
**UNM_0222**		VILLAGE PARK MEDICAL, 103, 45485 KNIGHT RD	CHILLIWACK	BC	V2R 3G3	CANADA	49.1398362	-121.96582	2 FHA	Fraser	3
**UNM_0223**		4556 GERTRUDE ST	PORT ALBERNI	BC	V9Y 6K1	CANADA	49.2578064	-124.8109451	4 VIHA	Vancouver Island	3
**UNM_0224**		101, 45625 HODGINS AVE	CHILLIWACK	BC	V2P 1P2	CANADA	49.167376	-121.9620983	2 FHA	Fraser	4
**UNM_0225**		104, 45744 GAETZ ST	CHILLIWACK	BC	V2R 3P1	CANADA	49.1360223	-121.9578967	2 FHA	Fraser	3
**UNM_0226**		475, 4789 KINGSWAY	BURNABY	BC	V5H 0A3	CANADA	49.2281054	-122.9968324	2 FHA	Fraser	4
**UNM_0227**		NISGA'A VALLEY HEALTH AUTHORITY, 4920 TAIT ST	GITLAXT'AAMIKS	BC	V0J 1A0	CANADA	55.1989799	-129.0733867	5 NHA	Northern	2
**UNM_0228**		495 DUNSMUIR ST SUITE A	NANAIMO	BC	V9R 6B9	CANADA	49.1630153	-123.9376363	4 VIHA	Vancouver Island	2
**UNM_0229**		495 40 AVE W	VANCOUVER	BC	V5Y 2R5	CANADA	49.2344777	-123.1154982	3 VCHA	Vancouver Coastal	2
**UNM_0230**		4955 PROSPECT LAKE RD	VICTORIA	BC	V9E 1J5	CANADA	48.516083	-123.4458552	4 VIHA	Vancouver Island	2
**UNM_0231**		498 KINGSWAY	VANCOUVER	BC	V5T 3J9	CANADA	49.2575955	-123.093415	3 VCHA	Vancouver Coastal	2
**UNM_0232**		5017 47A AVE	DELTA	BC	V4K 1T9	CANADA	49.0895026	-123.0844849	2 FHA	Fraser	3
**UNM_0233**		FLOOR 22, 2280, 510 WEST GEORGIA ST	VANCOUVER	BC	V6B 0M3	CANADA	49.2811779	-123.1169631	3 VCHA	Vancouver Coastal	2
**UNM_0234**		BX MEDICAL, 204, 5100 ANDERSON WAY	VERNON	BC	V1T 9V2	CANADA	50.2843481	-119.2741751	1 IHA	Interior	2
**UNM_0235**		5143 CANOE PASS WAY	DELTA	BC	V4M 0B2	CANADA	49.0422757	-123.0813536	2 FHA	Fraser	2
**UNM_0236**		104, 515 MAIN ST	VANCOUVER	BC	V6A 2V1	CANADA	49.2800436	-123.1001713	3 VCHA	Vancouver Coastal	2
**UNM_0237**		SIMON FRASER UNIVERSITY, VANCOUVER CAMPUS, 300, 515 HASTINGS ST W	VANCOUVER	BC	V6B 5K3	CANADA	49.284199	-123.1120152	3 VCHA	Vancouver Coastal	2
**UNM_0238**		COMMUNITY FIRST HEALTH COOP, 518 LAKE ST, SUITE 203, B	NELSON	BC	V1L 4C6	CANADA	49.4937306	-117.2943118	1 IHA	Interior	2
**UNM_0239**		301, 520 ST PAUL ST	KAMLOOPS	BC	V2C 2J9	CANADA	50.6743061	-120.3286931	1 IHA	Interior	2
**UNM_0240**		CHINATOWN MAIN MEDICAL, 523 MAIN ST	VANCOUVER	BC	V6A 2V1	CANADA	49.2799806	-123.1001752	3 VCHA	Vancouver Coastal	2
**UNM_0241**		202, 525 HIGHWAY 97 S	KELOWNA	BC	V1Z 4C9	CANADA	49.8835169	-119.492294	1 IHA	Interior	2
**UNM_0242**	PO BOX 20077	525 BROADWAY W	VANCOUVER	BC	V5Z 0C1	CANADA	49.2632871	-123.1161913	3 VCHA	Vancouver Coastal	2
**UNM_0243**		5320 VICTORIA DR	VANCOUVER	BC	V5P 3V7	CANADA	49.2367033	-123.065025	3 VCHA	Vancouver Coastal	2
**UNM_0244**		536 HERALD ST UNIT 11	VICTORIA	BC	V8W 1S6	CANADA	48.4306217	-123.3679308	4 VIHA	Vancouver Island	2
**UNM_0245**		275, 546 ST PAUL ST	KAMLOOPS	BC	V2C 5T1	CANADA	50.6742308	-120.3282766	1 IHA	Interior	3
**UNM_0246**		101, 5461 12 AVE	DELTA	BC	V4M 2B2	CANADA	49.024931	-123.0724614	2 FHA	Fraser	3
**UNM_0247**		510, 550 BROADWAY W	VANCOUVER	BC	V5Z 0E9	CANADA	49.2629359	-123.1165444	3 VCHA	Vancouver Coastal	2
**UNM_0248**		112, 5501 204 ST	LANGLEY	BC	V3A 5N8	CANADA	49.1026748	-122.6581415	2 FHA	Fraser	7
**UNM_0249**		201, 556 JOSEPHINE ST	NELSON	BC	V1L 1W6	CANADA	49.4919603	-117.2923678	1 IHA	Interior	2
**UNM_0250**		5570 CAMBIE ST	VANCOUVER	BC	V5Z 3A2	CANADA	49.2349046	-123.115976	3 VCHA	Vancouver Coastal	4
**UNM_0251**		5570 JOYCE ST	VANCOUVER	BC	V5R 4H6	CANADA	49.233155	-123.036404	3 VCHA	Vancouver Coastal	3
**UNM_0252**		5571 VICTORIA DR	VANCOUVER	BC	V5P 3W2	CANADA	49.2340339	-123.0659077	3 VCHA	Vancouver Coastal	2
**UNM_0253**		5573 VICTORIA DR	VANCOUVER	BC	V5P 3W2	CANADA	49.2340339	-123.0659077	3 VCHA	Vancouver Coastal	2
**UNM_0254**		5633 177B ST	SURREY	BC	V3S 4H9	CANADA	49.1047118	-122.7305352	2 FHA	Fraser	2
**UNM_0255**		380, 5655 CAMBIE ST	VANCOUVER	BC	V5Z 3A4	CANADA	49.2340007	-123.1167767	3 VCHA	Vancouver Coastal	2
**UNM_0256**		568 MOUNTAINVIEW SQ	KITIMAT	BC	V8C 2N2	CANADA	54.0516466	-128.6491597	5 NHA	Northern	3
**UNM_0257**		5715 CANAL RD	PENDER ISLAND	BC	V0N 2M1	CANADA	48.7652864	-123.2669839	4 VIHA	Vancouver Island	3
**UNM_0258**		WEST BAY WELLNESS, 101, 585 16 ST	WEST VANCOUVER	BC	V7V 3R8	CANADA	49.3294771	-123.1593144	3 VCHA	Vancouver Coastal	4
**UNM_0259**		101, 595 COLUMBIA ST W	KAMLOOPS	BC	V2C 1K7	CANADA	50.6723644	-120.3528973	1 IHA	Interior	3
**UNM_0260**		6051 GILBERT RD APT 208	RICHMOND	BC	V7C 3V3	CANADA	49.1697558	-123.1483045	3 VCHA	Vancouver Coastal	4
**UNM_0261**		120, 6180 BLUNDELL RD	RICHMOND	BC	V7C 4W7	CANADA	49.1549916	-123.1565148	3 VCHA	Vancouver Coastal	7
**UNM_0262**		6239 VICTORIA DR	VANCOUVER	BC	V5P 3X5	CANADA	49.2273093	-123.0660718	3 VCHA	Vancouver Coastal	3
**UNM_0263**		10, 626 MAIN ST	PENTICTON	BC	V2A 5C8	CANADA	49.4937847	-119.5894825	1 IHA	Interior	2
**UNM_0264**		320, 6330 FRASER ST	VANCOUVER	BC	V5W 3A4	CANADA	49.2270471	-123.0903034	3 VCHA	Vancouver Coastal	2
**UNM_0265**	PO BOX 31003	6389 120 ST, SUNSHINE VILLAGE	DELTA	BC	V4E 3M9	CANADA	49.1190708	-122.890776	2 FHA	Fraser	2
**UNM_0266**		6408 MAIN ST	VANCOUVER	BC	V5W 2V4	CANADA	49.2265118	-123.101195	3 VCHA	Vancouver Coastal	3
**UNM_0267**		6430 MAIN ST	VANCOUVER	BC	V5W 2V4	CANADA	49.2263219	-123.1015015	3 VCHA	Vancouver Coastal	3
**UNM_0268**		104, 645 FORT ST	VICTORIA	BC	V8W 1G2	CANADA	48.4244439	-123.366831	4 VIHA	Vancouver Island	6
**UNM_0269**		65 RICHMOND ST	NEW WESTMINSTER	BC	V3L 5P5	CANADA	49.2179305	-122.8971282	2 FHA	Fraser	3
**UNM_0270**		360, 650 41ST AVE W	VANCOUVER	BC	V5Z 2M9	CANADA	49.2319917	-123.1191254	3 VCHA	Vancouver Coastal	2
**UNM_0271**		6683 VICTORIA DR	VANCOUVER	BC	V5P 3Y2	CANADA	49.2236369	-123.0661803	3 VCHA	Vancouver Coastal	3
**UNM_0272**		COLLEGE OF PHYSICIANS SURGEONS OF BC, 300, 669 HOWE ST	VANCOUVER	BC	V6C 0B4	CANADA	49.2836521	-123.1188846	3 VCHA	Vancouver Coastal	7
**UNM_0273**		6704 PALFREY DR W	COLDSTREAM	BC	V1B 1A6	CANADA	50.2137687	-119.2658131	1 IHA	Interior	2
**UNM_0274**		6, 6828 128 ST	SURREY	BC	V3W 4C9	CANADA	49.1269655	-122.8675348	2 FHA	Fraser	2
**UNM_0275**		685 NARAMATA RD	PENTICTON	BC	V2A 8T3	CANADA	49.5180856	-119.559945	1 IHA	Interior	2
**UNM_0276**		103, 6935 120 ST	DELTA	BC	V4E 2A8	CANADA	49.129964	-122.8904454	2 FHA	Fraser	2
**UNM_0277**		105, 6935 120 ST	DELTA	BC	V4E 2A8	CANADA	49.1290965	-122.8909829	2 FHA	Fraser	2
**UNM_0278**		6935 HARVIE AVE	POWELL RIVER	BC	V8A 5H9	CANADA	49.8487503	-124.5234434	3 VCHA	Vancouver Coastal	4
**UNM_0279**		DR JOHANNES VAN HEERDEN, 694 SEYMOUR ST	KAMLOOPS	BC	V2C 2H2	CANADA	50.6751026	-120.3245161	1 IHA	Interior	2
**UNM_0280**		6962 ALBERNI ST	POWELL RIVER	BC	V8A 2C1	CANADA	49.8451713	-124.5221684	3 VCHA	Vancouver Coastal	2
**UNM_0281**		180, 6971 ELMBRIDGE WAY	RICHMOND	BC	V7C 0A5	CANADA	49.1736453	-123.1489023	3 VCHA	Vancouver Coastal	6
**UNM_0282**		FAMILY TREE HEALTH, 7019 ALBERNI ST	POWELL RIVER	BC	V8A 2C2	CANADA	49.8465584	-124.5196487	3 VCHA	Vancouver Coastal	6
**UNM_0283**		101, 7093 KING GEORGE BLVD	SURREY	BC	V3W 5A2	CANADA	49.1310033	-122.8459856	2 FHA	Fraser	2
**UNM_0284**		117, 7130 120 ST	SURREY	BC	V3W 3M8	CANADA	49.1325667	-122.8894631	2 FHA	Fraser	2
**UNM_0285**		103, 7130 120 ST	SURREY	BC	V3W 3M8	CANADA	49.1325667	-122.8894631	2 FHA	Fraser	3
**UNM_0286**	PO BOX 63008	HIGHGATE PO, 7155 KINGSWAY	BURNABY	BC	V5E 4J4	CANADA	49.2190296	-122.9562146	2 FHA	Fraser	2
**UNM_0287**		250, 7155 KINGSWAY	BURNABY	BC	V5E 0A6	CANADA	49.2190296	-122.9562146	2 FHA	Fraser	4
**UNM_0288**		7170 120 ST	SURREY	BC	V3W 3M8	CANADA	49.133436	-122.8899256	2 FHA	Fraser	2
**UNM_0289**		720 BROADWAY W	VANCOUVER	BC	V5Z 1G8	CANADA	49.263242	-123.1203899	3 VCHA	Vancouver Coastal	2
**UNM_0290**		114, 725 CARMI AVE	PENTICTON	BC	V2A 3G8	CANADA	49.4829978	-119.5754598	1 IHA	Interior	3

**UNM_0291**		1111, 7330 137 ST	SURREY	BC	V3W 1A3	CANADA	49.1360162	-122.8423982	2 FHA	Fraser	2
**UNM_0292**		230, 7360 WESTMINSTER HWY	RICHMOND	BC	V6X 1A1	CANADA	49.1698939	-123.141962	3 VCHA	Vancouver Coastal	2
**UNM_0293**		101, 7380 WESTMINSTER HWY	RICHMOND	BC	V6X 1A1	CANADA	49.1698938	-123.1416193	3 VCHA	Vancouver Coastal	2
**UNM_0294**		108, 7445 120 ST	DELTA	BC	V4C 0B3	CANADA	49.1386809	-122.8908904	2 FHA	Fraser	2
**UNM_0295**		510, 750 BROADWAY W	VANCOUVER	BC	V5Z 1H4	CANADA	49.2630118	-123.1210731	3 VCHA	Vancouver Coastal	2
**UNM_0296**		1317, 750 BROADWAY W	VANCOUVER	BC	V5Z 1J3	CANADA	49.2630118	-123.1210731	3 VCHA	Vancouver Coastal	3
**UNM_0297**		1013, 750 BROADWAY W	VANCOUVER	BC	V5Z 1H9	CANADA	49.2630118	-123.1210731	3 VCHA	Vancouver Coastal	3
**UNM_0298**		101, 7784 EAST SAANICH RD	SAANICHTON	BC	V8M 2B3	CANADA	48.5938832	-123.4175769	4 VIHA	Vancouver Island	2
**UNM_0299**		1101, 805 BROADWAY W	VANCOUVER	BC	V5Z 1K1	CANADA	49.2638177	-123.1222458	3 VCHA	Vancouver Coastal	2
**UNM_0300**		245, 809 41ST AVE W	VANCOUVER	BC	V5Z 2N6	CANADA	49.2341301	-123.1243196	3 VCHA	Vancouver Coastal	2
**UNM_0301**		205, 8120 COOK RD	RICHMOND	BC	V6Y 1T9	CANADA	49.1660378	-123.1351664	3 VCHA	Vancouver Coastal	2
**UNM_0302**		120, 8120 COOK RD	RICHMOND	BC	V6Y 1T9	CANADA	49.1660378	-123.1351664	3 VCHA	Vancouver Coastal	4
**UNM_0303**		237, 186, 8120 NO 2	RICHMOND	BC	V7C 5J8	CANADA	49.1539913	-123.1575216	3 VCHA	Vancouver Coastal	2
**UNM_0304**		150A, 8220 LANSDOWNE RD	RICHMOND	BC	V6X 1B9	CANADA	49.1733073	-123.133457	3 VCHA	Vancouver Coastal	3
**UNM_0305**		135, 8291 ACKROYD RD	RICHMOND	BC	V6X 3K5	CANADA	49.1720156	-123.1323556	3 VCHA	Vancouver Coastal	3
**UNM_0306**		831 ANDERS RD	WEST KELOWNA	BC	V1Z 1K1	CANADA	49.8600388	-119.5496283	1 IHA	Interior	2
**UNM_0307**		DR KJ SINGH INC, 101, 8338 120 ST	SURREY	BC	V3W 3N4	CANADA	49.1548935	-122.8900669	2 FHA	Fraser	3
**UNM_0308**	BOX 159	OYETOLA, 84 BROADWAY ST	NAKUSP	BC	V0G 1R0	CANADA	50.2384139	-117.7981406	1 IHA	Interior	2
**UNM_0309**		110, 8556 120 ST	SURREY	BC	V3W 3N5	CANADA	49.1583175	-122.890071	2 FHA	Fraser	3
**UNM_0310**		102, 8584 GRANVILLE ST	VANCOUVER	BC	V6P 4Z7	CANADA	49.2087878	-123.1401178	3 VCHA	Vancouver Coastal	2
**UNM_0311**		102, 8585 160TH ST	SURREY	BC	V4N 1G4	CANADA	49.1587056	-122.7791789	2 FHA	Fraser	2
**UNM_0312**		8630 120 ST	SURREY	BC	V3W 3N6	CANADA	49.1601376	-122.8900726	2 FHA	Fraser	2
**UNM_0313**		8685 120 ST	DELTA	BC	V4C 6R4	CANADA	49.1612875	-122.8904771	2 FHA	Fraser	2
**UNM_0314**		313, 877 GOLDSTREAM AVE	VICTORIA	BC	V9B 2X8	CANADA	48.4495587	-123.504632	4 VIHA	Vancouver Island	2
**UNM_0315**		215, 888 8 AVE W	VANCOUVER	BC	V5Z 3Y1	CANADA	49.2640136	-123.1235113	3 VCHA	Vancouver Coastal	7
**UNM_0316**		900 HASTINGS ST W, SUITE 800	VANCOUVER	BC	V6C 1E5	CANADA	49.2864734	-123.1162627	3 VCHA	Vancouver Coastal	5
**UNM_0317**		101, 9006 EDWARD ST	CHILLIWACK	BC	V2P 4E1	CANADA	49.1656569	-121.9636196	2 FHA	Fraser	3
**UNM_0318**		185, 911 YATES ST	VICTORIA	BC	V8V 4Y9	CANADA	48.4250452	-123.3576632	4 VIHA	Vancouver Island	2
**UNM_0319**		9146 MARY ST	CHILLIWACK	BC	V2P 4J1	CANADA	49.1681442	-121.9599871	2 FHA	Fraser	5
**UNM_0320**		9181 MAIN ST UNIT 106	CHILLIWACK	BC	V2P 4M9	CANADA	49.1691017	-121.9578732	2 FHA	Fraser	2
**UNM_0321**		921 CANADA AVE	DUNCAN	BC	V9L 1V2	CANADA	48.7842635	-123.7090808	4 VIHA	Vancouver Island	2
**UNM_0322**		340, 943 BROADWAY W	VANCOUVER	BC	V5Z 4E1	CANADA	49.2636765	-123.1250571	3 VCHA	Vancouver Coastal	2
**UNM_0323**		620, 943 BROADWAY W	VANCOUVER	BC	V5Z 4E1	CANADA	49.2636765	-123.1250571	3 VCHA	Vancouver Coastal	2
**UNM_0324**		SUITE 750, 943 W BROADWAY	VANCOUVER	BC	V5Z 4E1	CANADA	49.2636765	-123.1250571	3 VCHA	Vancouver Coastal	2
**UNM_0325**		260, 9600 CAMERON ST	BURNABY	BC	V3J 7N3	CANADA	49.2526712	-122.8981429	2 FHA	Fraser	2
**UNM_0326**		969 KANGAROO RD	VICTORIA	BC	V9C 4E1	CANADA	48.3794742	-123.5619288	4 VIHA	Vancouver Island	2
**UNM_0327**		210 9764 FIFTH ST	SIDNEY	BC	V8L 2X2	CANADA	48.6480936	-123.4010325	4 VIHA	Vancouver Island	2
**UNM_0328**		101, 9845 RESTHAVEN DR	SIDNEY	BC	V8L 3E7	CANADA	48.6499356	-123.4018932	4 VIHA	Vancouver Island	2
**UNM_0329**		1, 9966 POLLARD RD	WINFIELD	BC	V4V 1Z4	CANADA	50.0262429	-119.4060695	1 IHA	Interior	2
**UNM_0330**	BOX 1149		FORT ST JAMES	BC	V0J 1P0	CANADA			5 NHA	Northern	2
**UNM_0331**	PO BOX 23122	PLAZA 33	KELOWNA	BC	V1X 7K7	CANADA	49.8587963	-119.2535998	1 IHA	Interior	3
**UNM_0332**	PO BOX 1391		VERNON	BC	V1T 6N7	CANADA			1 IHA	Interior	2
**UNM_0333**	PO BOX 159		NAKUSP	BC	V0G 1R0	CANADA			1 IHA	Interior	2
**UNM_0334**	PO BOX 1700	UNIVERSITY OF VICTORIA, UNIVERSITY HEALTH SERVICES, STN CSC	VICTORIA	BC	V8W 2Y2	CANADA			4 VIHA	Vancouver Island	10
**UNM_0335**	PO BOX 17000	CF H SVCS C(P), STN FORCES	VICTORIA	BC	V9A 7N2	CANADA			4 VIHA	Vancouver Island	4
**UNM_0336**	PO BOX 1870	STN MAIN	SALMON ARM	BC	V1E 4P9	CANADA			1 IHA	Interior	2
**UNM_0337**	PO BOX 189		TOFINO	BC	V0R 2Z0	CANADA			4 VIHA	Vancouver Island	2
**UNM_0338**	PO BOX 2278		100 MILE HOUSE	BC	V0K 2E0	CANADA			1 IHA	Interior	2
**UNM_0339**	PO BOX 27024	CITYVIEW PO	KAMLOOPS	BC	V2E 0B2	CANADA			1 IHA	Interior	2
**UNM_0340**	PO BOX 32301	DOCTOR, 1 DOMESTIC RPO YVR TERMINAL, LEVEL	RICHMOND	BC	V7B 1W2	CANADA			3 VCHA	Vancouver Coastal	3
**UNM_0341**	PO BOX 346	STN MAIN	DUNCAN	BC	V9L 3X5	CANADA			4 VIHA	Vancouver Island	2
**UNM_0342**	PO BOX 3760		SMITHERS	BC	V0J 2N0	CANADA			5 NHA	Northern	2
**UNM_0343**	PO BOX 379		CHRISTINA LAKE	BC	V0H 1E0	CANADA			1 IHA	Interior	2
**UNM_0344**	PO BOX 389		SQUAMISH	BC	V8B 0A3	CANADA			3 VCHA	Vancouver Coastal	4
**UNM_0345**	PO BOX 400		GRAND FORKS	BC	V0H 1H0	CANADA			1 IHA	Interior	2
**UNM_0346**	PO BOX 458	NORTHERN ROOTS PRIMARY CARE	SMITHERS	BC	V0J 2N0	CANADA			5 NHA	Northern	3
**UNM_0347**	PO BOX 4700	STN TERMINAL	VANCOUVER	BC	V6B 1J1	CANADA			3 VCHA	Vancouver Coastal	2
**UNM_0348**	PO BOX 47088	RPO CITY SQ RPO CITY SQUARE	VANCOUVER	BC	V5Z 4L6	CANADA			3 VCHA	Vancouver Coastal	2
**UNM_0349**	PO BOX 47136	CITY SQUARE	VANCOUVER	BC	V5Z 4L6	CANADA			3 VCHA	Vancouver Coastal	2
**UNM_0350**	PO BOX 487		PRINCE RUPERT	BC	V8J 3R2	CANADA			5 NHA	Northern	2
**UNM_0351**	PO BOX 52007		SIDNEY	BC	V8L 5V9	CANADA			4 VIHA	Vancouver Island	2
**UNM_0352**	PO BOX 5253	STN B	VICTORIA	BC	V8R 6N4	CANADA			4 VIHA	Vancouver Island	2
**UNM_0353**	PO BOX 56625	RPO LOUGHEED MALL	BURNABY	BC	V3J 7W2	CANADA			2 FHA	Fraser	3
**UNM_0354**	PO BOX 590		REVELSTOKE	BC	V0E 2S0	CANADA			1 IHA	Interior	7
**UNM_0355**	PO BOX 6000		BURNS LAKE	BC	V0J 1E0	CANADA			5 NHA	Northern	3
**UNM_0356**	PO BOX 60437	FRASER PO	VANCOUVER	BC	V5W 4B5	CANADA			3 VCHA	Vancouver Coastal	2
**UNM_0357**	PO BOX 610		FRUITVALE	BC	V0G 1L0	CANADA			1 IHA	Interior	6
**UNM_0358**	PO BOX 624		SMITHERS	BC	V0J 2N0	CANADA			5 NHA	Northern	2
**UNM_0359**	PO BOX 638		SECHELT	BC	V0N 3A0	CANADA			3 VCHA	Vancouver Coastal	3
**UNM_0360**	PO BOX 820	SPARLING EAST MEDICAL	FERNIE	BC	V0B 1M0	CANADA			1 IHA	Interior	5
**UNM_0361**	PO BOX 850		LILLOOET	BC	V0K 1V0	CANADA			1 IHA	Interior	4
**UNM_0362**	PO BOX 9200		SQUAMISH	BC	V8B 0C1	CANADA			3 VCHA	Vancouver Coastal	8
**UNM_0363**	PO BOX 98978	OLYMPIC PO	VANCOUVER	BC	V5Y 0J9	CANADA			3 VCHA	Vancouver Coastal	2
**UNM_0364**	PO BOX 99		FORT LANGLEY	BC	V1M 2R4	CANADA			2 FHA	Fraser	2
**UNM_0365**	PO BOX 99182	DAVIE PO	VANCOUVER	BC	V6G 0B7	CANADA			3 VCHA	Vancouver Coastal	2
**UNM_0366**	PO BOX 998	STN MAIN	MERRITT	BC	V1K 1B8	CANADA			1 IHA	Interior	2
**UNM_0367**		REBALANCEMD, 3551, 104 BLANSHARD ST N	VICTORIA	BC	V8Z 0B9	CANADA	48.4337557	-123.3625051	4 VIHA	Vancouver Island	2
**UPC_0001**		KELOWNA URGENT AND PRIMARY CARE CENTRE, 1141 Harvey Avenue	Kelowna	BC	V1Y 6E8	CANADA	49.8830837	-119.4754685	1 IHA	Interior	4
**UPC_0002**		REACH URGENT AND PRIMARY CARE CENTRE, 1145 Commercial Drive	Vancouver	BC	V5L 3X3	CANADA	49.2743852	-123.0696418	3 VCHA	Vancouver Coastal	10
**UPC_0003**		CITY CENTRE URGENT AND PRIMARY CARE CENTRE, 1290 Hornby Street	Vancouver	BC	V6Z 1W2	CANADA	49.2772743	-123.1293398	3 VCHA	Vancouver Coastal	15
**UPC_0009**		VERNON URGENT AND PRIMARY CARE CENTRE, 101-3105 28th Avenue	Vernon	BC	V1T 8Y4	CANADA	50.2625031	-119.272591	1 IHA	Interior	2
**UPC_0010**		JAMES BAY URGENT AND PRIMARY CARE CENTRE, 547 Michigan Street	Victoria	BC	V8V 1S5	CANADA	48.4167543	-123.371306	4 VIHA	Vancouver Island	4
**UPC_0011**		WESTSHORE URGENT AND PRIMARY CARE CENTRE, 582 Goldstream Avenue	Victoria	BC	V9W 2W7	CANADA	48.4468276	-123.4871348	4 VIHA	Vancouver Island	2
**UPC_0012**		SURREY-NEWTON URGENT AND PRIMARY CARE CENTRE, 6830 King George Blvd.	Surrey	BC	V3W 4Z9	CANADA	49.1265358	-122.8451239	2 FHA	Fraser	1
**WIC_0003**		NORTH PEACE PRIMARY CARE CLINIC, 10011 96th Street	Fort St John	BC	V1J 3P3	CANADA	56.2475137	-120.8396942	5 NHA	Northern	21
**WIC_0004**		BURNSIDE FAMILY MEDICAL CLINIC, 101 Burnside Road W	Victoria	BC	V9A 1B7	CANADA	48.4468486	-123.3794777	4 VIHA	Vancouver Island	8
**WIC_0005**		WELLNESS FIRST MEDICAL CLINIC, 140-10111 No. 3 Road	Richmond	BC	V7A 1G3	CANADA	49.1396916	-123.136772	3 VCHA	Vancouver Coastal	2
**WIC_0006**		DENNING HEALTH GROUP, 3110-10153 King George Highway	Surrey	BC	V3T 2W1	CANADA	49.1855931	-122.8473969	2 FHA	Fraser	1
**WIC_0007**		VIVA CARE, 2151-10153 King George Blvd.	Surrey	BC	V3T 2W3	CANADA	49.1858825	-122.8458958	2 FHA	Fraser	1
**WIC_0008**		COAST MEDICAL, 1018 Seymour Street	Vancouver	BC	V6B 3M6	CANADA	49.2783299	-123.1223508	3 VCHA	Vancouver Coastal	5
**WIC_0009**		MILE ZERO CLINIC, 121-10200 8th Street	Dawson Creek	BC	V1G 3P8	CANADA	55.7570619	-120.2228765	5 NHA	Northern	1
**WIC_0010**		UNA MEDICAL CLINIC, 1027 West 15th Avenue	Vancouver	BC	V6H 1R7	CANADA	49.2578574	-123.1275009	3 VCHA	Vancouver Coastal	5
**WIC_0011**		VIVA CARE, 1000-10355 152nd Street	Surrey	BC	V3R 1N3	CANADA	49.1888291	-122.8043356	2 FHA	Fraser	1
**WIC_0012**		ULTIMA MEDICAL SERVICES, 1055 Dunsmuir Street	Vancouver	BC	V7X 1L4	CANADA	49.2865896	-123.1209561	3 VCHA	Vancouver Coastal	1
**WIC_0013**		LOYAL MEDICAL CLINIC, 112-1055 W Georgia Street	Vancouver	BC	V6E 3P1	CANADA	49.2854092	-123.1219503	3 VCHA	Vancouver Coastal	5
**WIC_0014**		LONSDALE MEDICAL CLINIC, 110-1100 Lonsdale Avenue	North Vancouver	BC	V7M 2H3	CANADA	49.3181729	-123.0718856	3 VCHA	Vancouver Coastal	9
**WIC_0015**		GIBSONS MEDICAL ASSOCIATES, 211-1100 Sunshine Coast Highway	Gibsons	BC	V0N 1V7	CANADA	49.4065803	-123.5303743	3 VCHA	Vancouver Coastal	15
**WIC_0016**		CARE POINT MEDICAL AND WELLNESS CLINICS, 1123 Davie Street	Vancouver	BC	V6E 1N2	CANADA	49.2809898	-123.1315423	3 VCHA	Vancouver Coastal	4
**WIC_0018**		KOOTENAY LAKE MEDICAL CLINIC, 1140 Lakeside Drive	Nelson	BC	V1L 5Z3	CANADA	49.4995576	-117.2898345	1 IHA	Interior	3
**WIC_0019**		INTEGRATED WELLNESS MEDICAL CLINIC, 502-1150 Marine Drive	North Vancouver	BC	V7P 1S8	CANADA	49.324316	-123.1056673	3 VCHA	Vancouver Coastal	5
**WIC_0020**		ESQUIMALT MEDICAL CLINIC, 3-1153 Esquimalt Road	Victoria	BC	V9A 3N7	CANADA	48.4299531	-123.409933	4 VIHA	Vancouver Island	9
**WIC_0021**		WAVE MEDICAL CLINIC, 201-1160 Burrard Street	Vancouver	BC	V6Z 2E8	CANADA	49.2792372	-123.1282976	3 VCHA	Vancouver Coastal	2
**WIC_0022**		HEALTHVUE MEDICAL CLINIC, 180-11700 Cambie Road	Richmond	BC	V6X 1L5	CANADA	49.1845147	-123.093212	3 VCHA	Vancouver Coastal	7
**WIC_0023**		NORTHSHORE MEDICAL CLINIC, 101-1171 Marine Drive	North Vancouver	BC	V7P 1T1	CANADA	49.3233767	-123.1065789	3 VCHA	Vancouver Coastal	3

**WIC_0024**		DENMAN MEDICAL CENTRE, 1175 Denman Street	Vancouver	BC	V6G 2N1	CANADA	49.2879633	-123.1411309	3 VCHA	Vancouver Coastal	7
**WIC_0025**		KEEFER MEDICAL CLINIC, 118 Keefer Street	Vancouver	BC	V6A 1X4	CANADA	49.2792923	-123.1019513	3 VCHA	Vancouver Coastal	4
**WIC_0026**		TURTLE BAY MEDICAL CLINIC, 802-11850 Oceola Road	Lake Country	BC	V4V 2T5	CANADA	50.0543298	-119.4124848	1 IHA	Interior	8
**WIC_0027**		IHEALTHMD MEDICAL CENTRE, 403-1200 Lonsdale Avenue	North Vancouver	BC	V7M 3H6	CANADA	49.3192811	-123.0718981	3 VCHA	Vancouver Coastal	5
**WIC_0028**		LYNN VALLEY MEDICAL AND TRAVEL CLINIC, 102-1200 Lynn Valley Road	North Vancouver	BC	V7J 2A2	CANADA	49.3360334	-123.0408014	3 VCHA	Vancouver Coastal	5
**WIC_0029**		COTTONWOOD MEDICAL CLINIC, 102-12005 238B Street	Maple Ridge	BC	V4R 1W1	CANADA	49.2206931	-122.5614899	2 FHA	Fraser	1
**WIC_0030**		DENNING HEALTH GROUP, 115-12080 Nordel Way	Surrey	BC	V3W 1P6	CANADA	49.1610954	-122.8886102	2 FHA	Fraser	4
**WIC_0031**		NEW PITT MEADOWS MEDICAL CLINIC, 12181 Harris Road	Pitt Meadows	BC	V3Y 2E9	CANADA	49.2240382	-122.6902248	2 FHA	Fraser	4
**WIC_0033**		TRITON MEDICAL CLINIC, 102-12565 88th Avenue	Surrey	BC	V3W 3J7	CANADA	49.1641663	-122.8748725	2 FHA	Fraser	4
**WIC_0034**		CAREFINITI HEALTH CORPORATION, 101-1277 Marine Drive	North Vancouver	BC	V7P 1T3	CANADA	49.3236058	-123.1097559	3 VCHA	Vancouver Coastal	3
**WIC_0035**		KWANTLEN MEDICAL CLINIC, 12818 72nd Avenue	Surrey	BC	V3W 2M9	CANADA	49.13371	-122.8673821	2 FHA	Fraser	2
**WIC_0036**		MOSS ROCK MEDICAL CENTRE, 1282 Fairfield Road	Victoria	BC	V8V 4W3	CANADA	48.4157697	-123.3491836	4 VIHA	Vancouver Island	9
**WIC_0038**		YALETOWN MEDICAL CLINIC, 1296 Pacific Boulevard	Vancouver	BC	V6Z 2V1	CANADA	49.2732254	-123.1227339	3 VCHA	Vancouver Coastal	5
**WIC_0039**		ELJEN MEDICAL CLINIC, 1300 111th Avenue	Dawson Creek	BC	V1G 2X5	CANADA	55.7493956	-120.2380091	5 NHA	Northern	4
**WIC_0040**		ROSEDALE MEDICAL ASSOCIATES, 200-13009 Rosedale Avenue	Summerland	BC	V0H 1Z0	CANADA	49.6012166	-119.6777108	1 IHA	Interior	4
**WIC_0041**		MERCY MEDICAL CLINIC, 1301 Lougheed Highway	Coquitlam	BC	V3K 6P9	CANADA	49.2349055	-122.8570652	2 FHA	Fraser	9
**WIC_0042**		CIVIC FAMILY HEALTH CENTRE, 110-13588 88th Avenue	Surrey	BC	V3W 3K8	CANADA	49.1626537	-122.8464846	2 FHA	Fraser	2
**WIC_0043**		VALLEYVIEW TREATMENT CENTRE, 1400 Cowichan Bay Road	Cobble Hill	BC	V0R 1L0	CANADA	48.7101595	-123.6082151	4 VIHA	Vancouver Island	1
**WIC_0044**		WEST VANCOUVER BEACHSIDE MEDICAL, 1405 Bellevue Avenue	West Vancouver	BC	V7T 1C3	CANADA	49.3273444	-123.154542	3 VCHA	Vancouver Coastal	2
**WIC_0045**		PLATEAU MEDICAL CLINIC, 1410 Parkway Boulevard	Coquitlam	BC	V3E 3J7	CANADA	49.2960702	-122.8031985	2 FHA	Fraser	2
**WIC_0046**		RIVER CITY MEDICAL CLINIC, 1424 Island Highway	Campbell River	BC	V9W 2E3	CANADA	50.0340816	-125.2509981	4 VIHA	Vancouver Island	2
**WIC_0047**		NORTHERN HEALTH AUTHORITY, 1445 5th Avenue	Valemount	BC	V0E 2Z0	CANADA	52.8318371	-119.2738684	5 NHA	Northern	5
**WIC_0048**		GUILDFORD MEDICAL CLINIC, 14650 104th Avenue	Surrey	BC	V3R 1M3	CANADA	49.1912385	-122.8160231	2 FHA	Fraser	2
**WIC_0049**		DISCOVERY MEDICAL CLINIC, 1477 Island Highway	Campbell River	BC	V9W 8E5	CANADA	50.0326112	-125.260868	4 VIHA	Vancouver Island	2
**WIC_0050**		FIRST CANADIAN MEDICAL CLINIC, 14888 104th Avenue	Surrey	BC	V3R 1M4	CANADA	49.1913505	-122.8097596	2 FHA	Fraser	1
**WIC_0051**		VERALIFE HEALTH CENTRE, 104-15122 72nd Avenue	Surrey	BC	V3S 2G2	CANADA	49.1336948	-122.8019433	2 FHA	Fraser	8
**WIC_0052**		COMMERCIAL DRIVE MEDICAL CLINIC, 1515 Commercial Drive	Vancouver	BC	V5L 3Y1	CANADA	49.2710695	-123.0697233	3 VCHA	Vancouver Coastal	1
**WIC_0053**		PANORAMA VILLAGE MEDICAL CLINIC, 15157 56th Avenue	Surrey	BC	V3S 9A5	CANADA	49.1053336	-122.8027414	2 FHA	Fraser	2
**WIC_0054**		COLEBROOK†MEDICAL CLINIC, 107-15240 56th Avenue	Surrey	BC	V3S 5K7	CANADA	49.1035474	-122.7990436	2 FHA	Fraser	10
**WIC_0055**		VIVA CARE, 125-15299 68th Avenue	Surrey	BC	V3S 2C1	CANADA	49.1269034	-122.7996153	2 FHA	Fraser	3
**WIC_0056**		SEYMOUR HEALTH CENTRE, 1530 West 7th Avenue	Vancouver	BC	V6J 1S3	CANADA	49.2653242	-123.1391941	3 VCHA	Vancouver Coastal	18
**WIC_0057**		SULLIVAN MEDICAL CLINIC, 108-15325 56th Avenue	Surrey	BC	V3S 0X9	CANADA	49.1045093	-122.7986626	2 FHA	Fraser	3
**WIC_0058**		HILLTOP MEDICAL CLINIC, 107-15331 16th Avenue	Surrey	BC	V4A 0C1	CANADA	49.0313232	-122.7986284	2 FHA	Fraser	1
**WIC_0059**		HIGHROADS MEDICAL CLINICS, 410-15355 24th Avenue	Surrey	BC	V4A 2H9	CANADA	49.0464844	-122.7966025	2 FHA	Fraser	4
**WIC_0061**		SOUTH SURREY MEDICAL CLINIC, 101-15850 26th Avenue	Surrey	BC	V3Z 2N6	CANADA	49.0485569	-122.78333	2 FHA	Fraser	9
**WIC_0062**		GORDON HEAD MEDICAL TREATMENT CENTRE, 103-1595 McKenzie Avenue	Victoria	BC	V8N 1A4	CANADA	48.4685733	-123.3331214	4 VIHA	Vancouver Island	4
**WIC_0063**		DENNING HEALTH GROUP, 106-15966 108th Avenue	Surrey	BC	V4N 5V6	CANADA	49.198601	-122.7799941	2 FHA	Fraser	4
**WIC_0065**		LONSDALE MEDICAL CLINICS, 1616 Lonsdale Avenue	North Vancouver	BC	V7M 2J3	CANADA	49.3236461	-123.0714434	3 VCHA	Vancouver Coastal	5
**WIC_0066**		CARE POINT MEDICAL AND WELLNESS CLINICS, 1623 Commercial Drive	Vancouver	BC	V5L 3Y3	CANADA	49.2701237	-123.0697494	3 VCHA	Vancouver Coastal	3
**WIC_0067**		OAK BAY MEDICAL CLINIC, 101-1640 Oak Bay Avenue	Victoria	BC	V8R 1B2	CANADA	48.4267034	-123.334592	4 VIHA	Vancouver Island	4
**WIC_0068**		LANSDOWNE TREATMENT CENTRE, 120-1641 Hillside Avenue	Victoria	BC	V8T 5G1	CANADA	48.4450083	-123.3337894	4 VIHA	Vancouver Island	2
**WIC_0069**		MEDI-KEL, 1715 Ellis Street	Kelowna	BC	V1Y 8M9	CANADA	49.8833078	-119.493433	1 IHA	Interior	1
**WIC_0070**		YYOUNG MEDICAL CLINIC AND PHARMACY, 1721 Main Street	Vancouver	BC	V5T 3B5	CANADA	49.2699167	-123.1007928	3 VCHA	Vancouver Coastal	2
**WIC_0071**		BRICKYARD MEDICAL CLINIC, 150-17475 56th Avenue	Surrey	BC	V3S 2X6	CANADA	49.1041117	-122.7383692	2 FHA	Fraser	3
**WIC_0072**		CLOVER CARE MEDICAL CLINIC, 102-17770 56th Avenue	Surrey	BC	V3S 1C7	CANADA	49.1041267	-122.7302613	2 FHA	Fraser	5
**WIC_0073**		AQUARIUS MEDICAL CLINIC, 202-179 Davie Street	Vancouver	BC	V6Z 2Y1	CANADA	49.273787	-123.1198134	3 VCHA	Vancouver Coastal	12
**WIC_0074**		SIMPLY WELLNESS, 1818 Kingsway	Vancouver	BC	V5N 2S7	CANADA	49.24594	-123.0674758	3 VCHA	Vancouver Coastal	7
**WIC_0075**		CLOVERHILL MEDICAL CLINIC, 111-18438 64th Avenue	Surrey	BC	V3S 1E9	CANADA	49.1186041	-122.7115047	2 FHA	Fraser	2
**WIC_0077**		CLAYTON HEIGHTS MEDICAL CLINIC, 204-18730 Fraser Highway	Surrey	BC	V3S 7Y4	CANADA	49.1243903	-122.7032867	2 FHA	Fraser	3
**WIC_0079**		OCEAN MEDICAL, 1884 Marine Drive	West Vancouver	BC	V7V 1J6	CANADA	49.3285836	-123.1637579	3 VCHA	Vancouver Coastal	11
**WIC_0081**		COLWOOD MEDICAL TREATMENT CENTRE, 102-1910 Sooke Road	Victoria	BC	V9B 1V7	CANADA	48.441184	-123.470524	4 VIHA	Vancouver Island	3
**WIC_0082**		HILLSIDE MEDICAL CLINIC, 114-1920 Summit Drive	Kelowna	BC	V1V 3E9	CANADA	49.9015403	-119.4526587	1 IHA	Interior	8
**WIC_0083**		BEYOND HEALTH AND MEDICAL CENTRE, 101-19211 Fraser Highway	Surrey	BC	V3S 7C9	CANADA	49.1189669	-122.6916023	2 FHA	Fraser	4
**WIC_0084**		CENTRAL SAANICH MEDICAL CLINIC, 1932 Mount Newton Cross Road	Saanichton	BC	V8M 2B1	CANADA	48.5947257	-123.4188577	4 VIHA	Vancouver Island	2
**WIC_0085**		SPALL MEDICAL CENTRE, 150-1940 Harvey Avenue	Kelowna	BC	V1Y 8J8	CANADA	49.8828073	-119.4537962	1 IHA	Interior	5
**WIC_0086**		LONSDALE MEDICAL CLINICS, 108-1940 Lonsdale Avenue	North Vancouver	BC	V7M 2K2	CANADA	49.3271904	-123.0719484	3 VCHA	Vancouver Coastal	13
**WIC_0087**		WILLOWBROOK MEDICAL CLINIC, 158A-19653 Willowbrook Drive	Langley	BC	V2Y 1A5	CANADA	49.11576	-122.6793653	2 FHA	Fraser	2
**WIC_0089**		VALLEY CENTRE MEDICAL CLINIC, 19851 Willowbrook Drive	Langley	BC	V2Y 1A7	CANADA	49.1179171	-122.6733484	2 FHA	Fraser	4
**WIC_0090**		ORCHARD MEDICAL CENTRE, 104-1990 Cooper Road	Kelowna	BC	V1Y 8K5	CANADA	49.8776938	-119.4433789	1 IHA	Interior	4
**WIC_0091**		FAIRFIELD MEDICAL TREATMENT CENTRE, 302-1990 Fort Street	Victoria	BC	V8R 6V4	CANADA	48.4323767	-123.3228287	4 VIHA	Vancouver Island	1
**WIC_0092**		MOUNT IDA MEDICAL CENTRE, 200 Trans Canada Highway SW	Salmon Arm	BC	V1E 1V4	CANADA	50.6991521	-119.2872131	1 IHA	Interior	4
**WIC_0093**		HEALTHWAY MEDICAL CLINIC, 20200 Fraser Highway	Langley	BC	V3A 4E4	CANADA	49.1060814	-122.6625836	2 FHA	Fraser	2
**WIC_0094**		WILLOUGHBY MEDICAL CENTRE, 20202 66th Avenue	Langley	BC	V2Y 1P3	CANADA	49.1208293	-122.6629203	2 FHA	Fraser	5
**WIC_0095**		MAPLE MEDICAL CLINIC, 103-2025 West Broadway	Vancouver	BC	V6J 1Z6	CANADA	49.2638554	-123.1511614	3 VCHA	Vancouver Coastal	4
**WIC_0096**		PACIFIC MEDICAL CLINIC, 2032 Kingsway	Vancouver	BC	V5N 2T3	CANADA	49.244801	-123.0645789	3 VCHA	Vancouver Coastal	2
**WIC_0097**		ABBOTSFORD VILLAGE MEDICAL CLINIC, 302-2050 Sumas Way	Abbotsford	BC	V2S 2C7	CANADA	49.0390766	-122.2701053	2 FHA	Fraser	2
**WIC_0098**		KERRISDALE MEDICAL CLINIC, 2077 West 42nd Avenue	Vancouver	BC	V6M 2B4	CANADA	49.2338558	-123.1540458	3 VCHA	Vancouver Coastal	12
**WIC_0099**		PEACH CITY MEDICAL AND URGENT CARE CLINIC, 166-2111 Main Street	Penticton	BC	V2A 6W6	CANADA	49.4728095	-119.5838727	1 IHA	Interior	4
**WIC_0100**		VIVA CARE, 401-21183 88th Avenue	Langley	BC	V1M 2G5	CANADA	49.162667	-122.6338781	2 FHA	Fraser	2
**WIC_0101**		HIGHROADS MEDICAL CLINICS, 2145 Louie Drive	Kelowna	BC	V4T 3E6	CANADA	49.8403284	-119.6122849	1 IHA	Interior	4
**WIC_0102**		VIVA CARE, 2150 Hawkins Street	Port Coquitlam	BC	V3B 0G6	CANADA	49.2545079	-122.7353803	2 FHA	Fraser	3
**WIC_0104**		SALVEO MEDICAL CLINIC, 2155 Ferry Avenue	Prince George	BC	V2N 5E8	CANADA	53.888623	-122.7694709	5 NHA	Northern	2
**WIC_0105**		SICAMOUS MEDICAL CLINIC, 217 Finlayson Street	Sicamous	BC	V0E 2V0	CANADA	50.8364286	-118.9896402	1 IHA	Interior	1
**WIC_0106**		THE BRIDGE MEDICAL CLINIC, 2207F Glenmore Road	Campbell River	BC	V9H 1E1	CANADA	49.8698329	-125.1286184	4 VIHA	Vancouver Island	1
**WIC_0108**		WEST KELOWNA WALK-IN AND FAMILY PRACTICE, 105-2231 Louie Drive	West Kelowna	BC	V4T 3K3	CANADA	49.8377731	-119.6157393	1 IHA	Interior	2
**WIC_0109**		MURALS MEDICAL CLINIC, 100-22314 Fraser Highway	Langley	BC	V3A 8M6	CANADA	49.0905941	-122.6062436	2 FHA	Fraser	3
**WIC_0110**		PURE CARE CLINIC, 105-22470 Dewdney Trunk Road	Maple Ridge	BC	V2X 5Z6	CANADA	49.2197886	-122.6000132	2 FHA	Fraser	1
**WIC_0111**		LAKESHORE MEDICAL CENTRE, 2280 Baron Road	Kelowna	BC	V1X 7W3	CANADA	49.8810241	-119.4317675	1 IHA	Interior	6
**WIC_0112**		HASTINGS SUNRISE MEDICAL CLINIC, 102-2280 E Hastings Street	Vancouver	BC	V5L 1V4	CANADA	49.2808918	-123.0582838	3 VCHA	Vancouver Coastal	7
**WIC_0113**		KHATSAHLANO MEDICAL CLINICS, 101A-230 Cook Street	Victoria	BC	V8V 3X3	CANADA	48.4140962	-123.3572765	4 VIHA	Vancouver Island	6
**WIC_0115**		MERCY MEDICAL CLINIC, 2332 160th Street	Surrey	BC	V3S 9N6	CANADA	49.0438341	-122.7764076	2 FHA	Fraser	2
**WIC_0116**		WHEELHOUSE INTEGRATIVE MEDICAL CLINIC, 107-2349 Millstream Road	Victoria	BC	V9B 3R5	CANADA	48.4639094	-123.4990809	4 VIHA	Vancouver Island	2
**WIC_0117**		SHORELINE MEDICAL, 2A-2379 Bevan Avenue	Sidney	BC	V8L 4M9	CANADA	48.6473855	-123.4008276	4 VIHA	Vancouver Island	14
**WIC_0118**		ROBSON MEDICAL CENTRE, 238 Robson Street	Vancouver	BC	V6B 6A1	CANADA	49.2785132	-123.1155079	3 VCHA	Vancouver Coastal	1
**WIC_0119**		TOWNE CENTRE MEDICAL CLINIC, 19-2475 Dobbin Road	West Kelowna	BC	V4T 2E9	CANADA	49.8284216	-119.6308601	1 IHA	Interior	3
**WIC_0120**		BROADWAY STATION MEDICAL CLINIC, 2516 Commercial Drive	Vancouver	BC	V5N 4C2	CANADA	49.2619422	-123.0696239	3 VCHA	Vancouver Coastal	3
**WIC_0121**		GRANVILLE MEDICAL CLINIC, 2578 Granville Street	Vancouver	BC	V6H 3G8	CANADA	49.2629517	-123.1384591	3 VCHA	Vancouver Coastal	3
**WIC_0122**		QUADRA VILLAGE MEDICAL CLINIC, 2631 Quadra Street	Victoria	BC	V8T 4E4	CANADA	48.4383861	-123.3581457	4 VIHA	Vancouver Island	3
**WIC_0123**		KHATSAHLANO MEDICAL CLINICS, 2685 West Broadway	Vancouver	BC	V6K 2G2	CANADA	49.2642879	-123.1655319	3 VCHA	Vancouver Coastal	7
**WIC_0125**		ALLWOOD MEDICAL CENTRE, 102-2752 Allwood Street	Abbotsford	BC	V2T 3R7	CANADA	49.0517312	-122.3198134	2 FHA	Fraser	2
**WIC_0126**		SOLTANI MEDICAL CLINIC, 6-2755 Lougheed Highway	Port Coquitlam	BC	V3B 5Y9	CANADA	49.2738341	-122.7885553	2 FHA	Fraser	4
**WIC_0127**		BEVERLY MEDICAL CLINIC, 201-2763 Beverly Street	Duncan	BC	V9L 6X2	CANADA	48.7857453	-123.7080025	4 VIHA	Vancouver Island	7
**WIC_0128**		ALDER MEDICAL CENTRE, 277 Evergreen Road	Campbell River	BC	V9W 5Y4	CANADA	50.0071695	-125.2411602	4 VIHA	Vancouver Island	13
**WIC_0130**		ABBY CENTRAL MEDICAL AND DENTAL CLINIC, 2855 Gladwin Road	Abbotsford	BC	V2T 6Y4	CANADA	49.0540272	-122.3168407	2 FHA	Fraser	1
**WIC_0131**		COWICHAN WAY CLINIC, 291 Cowichan Way	Duncan	BC	V9L 6P5	CANADA	48.7743604	-123.7036524	4 VIHA	Vancouver Island	1
**WIC_0132**		CAREMART MEDICAL, 2929 Barnet Highway	Coquitlam	BC	V3B 5R5	CANADA	49.2792693	-122.7980479	2 FHA	Fraser	1
**WIC_0133**		SHUSWAP PROVIDENCE MEDICAL CLINIC, 2991 10th Avenue SW	Salmon Arm	BC	V1E 3J9	CANADA	50.6988461	-119.3190169	1 IHA	Interior	3
**WIC_0134**		ABLE CARE MEDICAL CLINIC, 56-2991 Lougheed Highway	Coquitlam	BC	V3B 6J6	CANADA	49.2774225	-122.7950141	2 FHA	Fraser	8
**WIC_0135**		SUNWOOD MEDICAL CLINIC, 3000 Lougheed Highway	Coquitlam	BC	V3B 1C5	CANADA	49.2736099	-122.793436	2 FHA	Fraser	2
**WIC_0136**		ROYAL COLUMBIA MEDICAL CLINIC, 107-301 East Columbia Street	New Westminster	BC	V3L 3W5	CANADA	49.2256281	-122.8928796	2 FHA	Fraser	3
**WIC_0137**		MD MEDICAL CENTRE, 3020 Louie Drive	West Kelowna	BC	V4T 3E1	CANADA	49.835718	-119.6185284	1 IHA	Interior	2
**WIC_0138**		ELLWOOD MEDICAL CLINIC, 100-30495 Cardinal Avenue	Abbotsford	BC	V2T 5R9	CANADA	49.0607782	-122.3788197	2 FHA	Fraser	3
**WIC_0139**		EDGEMONT MEDICAL CLINIC, 104-3053 Edgemont Boulvard	North Vancouver	BC	V7R 2N5	CANADA	49.3369247	-123.1016183	3 VCHA	Vancouver Coastal	2
**WIC_0140**		SUN PEAKS COMMUNITY HEALTH CENTRE, 3115 Creekside Way	Sun Peaks	BC	V0E 5N0	CANADA	50.8845302	-119.8903788	1 IHA	Interior	2

**WIC_0141**		VIVA CARE, 3122 Mount Lehman Road	Abbotsford	BC	V4X 0B3	CANADA	49.0579461	-122.3785062	2 FHA	Fraser	3
**WIC_0142**		TILLICUM MEDICAL CLINIC, 14-3170 Tillicum Road	Victoria	BC	V9A 7C5	CANADA	48.4533704	-123.3940427	4 VIHA	Vancouver Island	3
**WIC_0143**		PACIFIC MEDICAL CLINIC, 3185 Grandview Highway	Vancouver	BC	V5M 2E9	CANADA	49.2592546	-123.0365226	3 VCHA	Vancouver Coastal	6
**WIC_0144**		COMOX VALLEY MEDICAL CLINIC, 3199 Cliffe Avenue	Courtenay	BC	V9N 2L9	CANADA	49.6672092	-124.9810572	4 VIHA	Vancouver Island	2
**WIC_0145**		SHELBOURNE MEDICAL CLINIC, 150-3200 Shelbourne Street	Victoria	BC	V8P 5G8	CANADA	48.4485832	-123.3332233	4 VIHA	Vancouver Island	5
**WIC_0146**		STERLING CENTRE CLINIC, 101-3210 25th Avenue	Vernon	BC	V1T 1P1	CANADA	50.2603819	-119.2749639	1 IHA	Interior	1
**WIC_0148**		CEDAR CARE CLINIC, 104-32423 Lougheed Highway	Mission	BC	V2V 7B8	CANADA	49.1337774	-122.3270973	2 FHA	Fraser	4
**WIC_0149**		MISSION HILLS MEDICAL CLINIC, 107-32471 Lougheed Highway	Mission	BC	V2V 0C8	CANADA	49.1330011	-122.3257469	2 FHA	Fraser	3
**WIC_0150**		ARCHWAY COMMUNITY SERVICES, 101-32555 Simon Avenue	Abbotsford	BC	V2T 4Y2	CANADA	49.0495837	-122.3042761	2 FHA	Fraser	1
**WIC_0151**		DENNING HEALTH GROUP, 310-32700 South Fraser Way	Abbotsford	BC	V2T 4M5	CANADA	49.0499256	-122.3171101	2 FHA	Fraser	2
**WIC_0152**		MISSION MEDICAL CENTRE, 102-3320 Richter Street	Kelowna	BC	V1W 4V5	CANADA	49.8575241	-119.4897756	1 IHA	Interior	1
**WIC_0153**		PARK AND TILFORD MEDICAL TREATMENT CENTRE, 333 Brooksbank Avenue	North Vancouver	BC	V7J 3S8	CANADA	49.3090528	-123.0419468	3 VCHA	Vancouver Coastal	2
**WIC_0154**		MOUNT SEYMOUR MEDICAL CLINIC, 333 Seymour Boulevard	North Vancouver	BC	V7J 2J4	CANADA	49.3115536	-123.024775	3 VCHA	Vancouver Coastal	2
**WIC_0155**		UNIVERSITY OF NORTHERN BRITISH COLUMBIA, 3333 University Way	Prince George	BC	V2N 4Z9	CANADA	53.8875836	-122.8139433	5 NHA	Northern	2
**WIC_0156**		FOOTHILLS MEDICAL CLINIC, 203-3387 David Avenue	Coquitlam	BC	V3E 0K4	CANADA	49.2933762	-122.7554674	2 FHA	Fraser	5
**WIC_0157**		UPTOWN MEDICAL CLINIC, 3460 Saanich Road	Victoria	BC	V8X 1W7	CANADA	48.4540374	-123.3743087	4 VIHA	Vancouver Island	4
**WIC_0158**		COOK AND QUADRA MEDICAL CLINIC, 3461 Cook Street	Victoria	BC	V8X 1B3	CANADA	48.4551019	-123.3576434	4 VIHA	Vancouver Island	5
**WIC_0159**		SOUTH VANCOUVER MEDICAL CLINIC, 350 SE Marine Drive	Vancouver	BC	V5X 2S5	CANADA	49.2090685	-123.10039	3 VCHA	Vancouver Coastal	6
**WIC_0160**		SAANICH PLAZA MEDICAL CLINIC, 3544 Blanshard Street	Victoria	BC	V8X 1W3	CANADA	48.4563514	-123.3741666	4 VIHA	Vancouver Island	8
**WIC_0162**		MID-MAIN COMMUNITY HEALTH CENTRE, 3998 Main Street	Vancouver	BC	V5V 3P2	CANADA	49.2494931	-123.1006109	3 VCHA	Vancouver Coastal	8
**WIC_0164**		ANCRON MEDICAL CENTRE, 108-402 Baker Street	Nelson	BC	V1L 4H8	CANADA	49.4916244	-117.2948361	1 IHA	Interior	6
**WIC_0166**		QE PARK MEDICAL CLINIC, 4060 Cambie Street	Vancouver	BC	V5Z 2X8	CANADA	49.2494905	-123.1149628	3 VCHA	Vancouver Coastal	3
**WIC_0167**		FOREMED CLINIC, 420 Columbia Street	New Westminster	BC	V3L 1B1	CANADA	49.2042922	-122.9057727	2 FHA	Fraser	6
**WIC_0168**		DAWSON MEDICAL CLINIC, 4238 Dawson Street	Burnaby	BC	V5C 0B8	CANADA	49.2638994	-123.010933	2 FHA	Fraser	1
**WIC_0169**		POWELL RIVER MEDICAL CLINIC, 4280 Joyce Avenue	Powell River	BC	V8A 3A2	CANADA	49.832024	-124.5203548	3 VCHA	Vancouver Coastal	2
**WIC_0170**		TOWN PLAZA MEDICAL CLINIC, 40-4314 Main Street	Whistler	BC	V0N 1B4	CANADA	50.1171423	-122.955349	3 VCHA	Vancouver Coastal	4
**WIC_0171**		WHISTLER MEDICAL CLINIC, 4380 Lorimer Road	Whistler	BC	V8E 1A7	CANADA	50.1198186	-122.9551699	3 VCHA	Vancouver Coastal	15
**WIC_0173**		MANGO MEDICAL CLINIC, 4392 Beresford Street	Burnaby	BC	V5H 0E7	CANADA	49.2259095	-123.0059797	2 FHA	Fraser	5
**WIC_0174**		CARE POINT MEDICAL AND WELLNESS CLINICS, 100-4420 Chatterton Way	Victoria	BC	V8X 5J2	CANADA	48.4874856	-123.3831491	4 VIHA	Vancouver Island	2
**WIC_0175**		OLD ORCHARD MEDICAL CLINIC, 11-4429 Kingsway	Burnaby	BC	V5H 2A1	CANADA	49.2314081	-123.0051308	2 FHA	Fraser	7
**WIC_0176**		POINT GREY MEDICAL CLINIC, 4448 West 10th Avenue	Vancouver	BC	V6R 2H9	CANADA	49.2637562	-123.2076858	3 VCHA	Vancouver Coastal	6
**WIC_0177**		SPRING MEDICAL CENTRE, 4453 Lougheed Highway	Burnaby	BC	V5C 3Z2	CANADA	49.2665341	-123.0047659	2 FHA	Fraser	6
**WIC_0178**		J-VON MEDICAL CENTRE, 4468 Beresford Street	Burnaby	BC	V5H 0B8	CANADA	49.2250788	-123.0042899	2 FHA	Fraser	8
**WIC_0179**		KINETIC ENERGY HEALTH AND WELLNESS CENTRE, 207-450 Lansdowne Street	Kamloops	BC	V2C 1Y4	CANADA	50.6766685	-120.3283658	1 IHA	Interior	1
**WIC_0180**		BRENTWOOD MEDICAL CLINIC, 212-4567 Lougheed Highway	Burnaby	BC	V5C 3Z6	CANADA	49.2664886	-123.0016568	2 FHA	Fraser	1
**WIC_0182**		EDWARDS CROSSING MEDICAL, 9-45955 Yale Road	Chilliwack	BC	V2P 2M4	CANADA	49.1681835	-121.9583921	2 FHA	Fraser	3
**WIC_0184**		RICHMOND IN-STORE MEDICAL CLINIC, 4651 No. 3 Road	Richmond	BC	V6X 2C4	CANADA	49.1792161	-123.1385092	3 VCHA	Vancouver Coastal	2
**WIC_0185**		CANWAY MEDICAL CENTRE, 4695 Canada Way	Burnaby	BC	V5G 1K8	CANADA	49.2541427	-122.9895153	2 FHA	Fraser	3
**WIC_0186**		MARINE GATEWAY MEDICAL CLINIC, 301-470 SW Marine Drive	Vancouver	BC	V5X 0C4	CANADA	49.2102311	-123.1166169	3 VCHA	Vancouver Coastal	4
**WIC_0187**		CALEDONIAN CLINIC, 195-4750 Rutherford Road	Nanaimo	BC	V9T 4K6	CANADA	49.216944	-124.0266054	4 VIHA	Vancouver Island	17
**WIC_0188**		DELTA MEDICAL CENTER, 140-4977 Trenant Street	Delta	BC	V4K 2K5	CANADA	49.0923722	-123.0856466	2 FHA	Fraser	5
**WIC_0189**		NORTH OKANAGAN MEDICAL CLINIC, 5001 Anderson Way	Vernon	BC	V1T 9V1	CANADA	50.284086	-119.2723954	1 IHA	Interior	2
**WIC_0190**		SINA PHARMACY AND HEALTH CENTRE, 505 Smithe Street	Vancouver	BC	V6B 6H1	CANADA	49.2794762	-123.1193502	3 VCHA	Vancouver Coastal	4
**WIC_0191**		CROSS ROADS CLINICS, 350-507 West Broadway	Vancouver	BC	V5Z 1E6	CANADA	49.2633184	-123.115353	3 VCHA	Vancouver Coastal	12
**WIC_0192**		CHETWYND PRIMARY CARE CLINIC, 5125 50th Street SW	Chetwynd	BC	V0C 1J0	CANADA	55.6954894	-121.6303491	5 NHA	Northern	2
**WIC_0193**		DENNING HEALTH GROUP, 5132 Ladner Trunk Road	Delta	BC	V4K 1W3	CANADA	49.0901888	-123.0801194	2 FHA	Fraser	6
**WIC_0194**		CARE POINT MEDICAL AND WELLNESS CLINICS, 5138 Joyce Street	Vancouver	BC	V5R 4H1	CANADA	49.2378026	-123.0317957	3 VCHA	Vancouver Coastal	2
**WIC_0195**		CONTINUUM MEDICAL CARE, 201-520 17th Street	West Vancouver	BC	V7V 3S8	CANADA	49.3286439	-123.1601634	3 VCHA	Vancouver Coastal	2
**WIC_0196**		AIRPORT WAY MEDICAL CLINIC, 5216 Airport Drive	Fort Nelson	BC	V0C 1R0	CANADA	58.8078731	-122.6948845	5 NHA	Northern	2
**WIC_0197**		CAULFIELD VILLAGE MEDICAL CLINIC, 220-5317 Headland Drive	West Vancouver	BC	V7W 3C5	CANADA	49.3503891	-123.2531819	3 VCHA	Vancouver Coastal	2
**WIC_0198**		CARE POINT MEDICAL AND WELLNESS CLINICS, 140-555 6th Street	New Westminster	BC	V3L 5H1	CANADA	49.2127795	-122.9197849	2 FHA	Fraser	4
**WIC_0199**		AVECINA MEDICAL CLINIC, 100-5581 204th Street	Langley	BC	V3A 1Z4	CANADA	49.103206	-122.6505988	2 FHA	Fraser	1
**WIC_0200**		MATTERS OF HEALTH MEDICAL CLINIC, 5591 West Boulevard	Vancouver	BC	V6M 3W6	CANADA	49.2357151	-123.1558951	3 VCHA	Vancouver Coastal	2
**WIC_0201**		ELICARE MEDICAL, 140-5611 Cooney Road	Richmond	BC	V6X 3J6	CANADA	49.1724739	-123.1319317	3 VCHA	Vancouver Coastal	2
**WIC_0202**		ALDER MEDICAL CLINIC, 2-575B Alder Avenue	100 Mile House	BC	V0K 2E1	CANADA	51.6386427	-121.2979623	1 IHA	Interior	1
**WIC_0203**		GLOVER MEDICAL CENTRE, 101-5796 Glover Road	Langley	BC	V3A 4H9	CANADA	49.1079097	-122.6502877	2 FHA	Fraser	5
**WIC_0204**		SALMON ARM MEDICAL CLINIC, 581 Hudson Avenue NE	Salmon Arm	BC	V1E 4N8	CANADA	50.7034328	-119.278602	1 IHA	Interior	3
**WIC_0205**		UNIVERSITY VILLAGE MEDICAL, 5933 Birney Avenue	Vancouver	BC	V6T 1W5	CANADA	49.2535612	-123.2360393	3 VCHA	Vancouver Coastal	2
**WIC_0206**		CALEDONIAN CLINIC, 6010 Brickyard Road	Nanaimo	BC	V9V 1S5	CANADA	49.2346378	-124.0261691	4 VIHA	Vancouver Island	3
**WIC_0207**		ELICARE MEDICAL, 228B-610 6th Street	New Westminster	BC	V3L 3C3	CANADA	49.2134839	-122.9217729	2 FHA	Fraser	4
**WIC_0208**		WEST STEVESTON MEDICAL CLINIC, 100-6168 London Road	Richmond	BC	V7E 0C1	CANADA	49.117269	-123.1581346	3 VCHA	Vancouver Coastal	3
**WIC_0209**		PACIFIC MEDICAL CLINIC, 6176 Fraser Street	Vancouver	BC	V5W 3A1	CANADA	49.2285234	-123.0902657	3 VCHA	Vancouver Coastal	1
**WIC_0210**		STATION SQUARE MEDICAL CLINIC, 150-6200 McKay Avenue	Burnaby	BC	V5H 4L7	CANADA	49.2278674	-123.0044669	2 FHA	Fraser	4
**WIC_0211**		COAL HARBOUR HEALTH CENTRE, 622 Bute Street	Vancouver	BC	V6E 3M1	CANADA	49.2876684	-123.1244098	3 VCHA	Vancouver Coastal	4
**WIC_0212**		NEWGEN MEDICAL CENTRE, 104-6321 King George Boulevard	Surrey	BC	V3X 1G1	CANADA	49.1175876	-122.8460318	2 FHA	Fraser	2
**WIC_0213**		VIVA CARE, 120-6345 120th Street	Delta	BC	V4E 2A6	CANADA	49.1181698	-122.8904664	2 FHA	Fraser	2
**WIC_0214**		BOUNDARY PARK MEDICAL CENTRE, 115-6350 120th Street	Surrey	BC	V3X 3K1	CANADA	49.1182335	-122.8901745	2 FHA	Fraser	4
**WIC_0215**		GREEN CROSS MEDICAL CLINIC AND PHARMACY, 115-6363 168th Street	Surrey	BC	V3S 3Y2	CANADA	49.1178356	-122.7568173	2 FHA	Fraser	1
**WIC_0216**		MEDICAL ARTS CENTRE, 103-650 South Terminal Avenue	Nanaimo	BC	V9R 5E2	CANADA	49.1642304	-123.9333756	4 VIHA	Vancouver Island	23
**WIC_0217**		KENSINGTON WALK-IN AND PAEDIATRIC MEDICAL CLINIC, 6548 Hastings Street	Burnaby	BC	V5B 1S2	CANADA	49.280274	-122.9671596	2 FHA	Fraser	11
**WIC_0222**		GEORGIA MEDICAL CLINIC, 683 Denman Street	Vancouver	BC	V6G 2L3	CANADA	49.2923611	-123.1342019	3 VCHA	Vancouver Coastal	3
**WIC_0223**		RAI MEDICAL CLINIC, 101-6905 120th Street	Delta	BC	V4E 2A8	CANADA	49.1287712	-122.8909498	2 FHA	Fraser	2
**WIC_0224**		SHORELINE MEDICAL, 102-7143 W Saanich Road	Brentwood Bay	BC	V8M 1P7	CANADA	48.5768217	-123.4468817	4 VIHA	Vancouver Island	9
**WIC_0226**		MERCY MEDICAL CLINIC, 7155 120th Street	Delta	BC	V4E 2B1	CANADA	49.1324949	-122.8904664	2 FHA	Fraser	4
**WIC_0228**		THE CLINIC AT CLAYTON HEIGHTS, 103-7170 188th Street	Surrey	BC	V4N 6R4	CANADA	49.132928	-122.7013525	2 FHA	Fraser	1
**WIC_0229**		HIGHGATE MEDICAL CLINIC, 103-7188 Kingsway	Burnaby	BC	V5E 1G3	CANADA	49.2182045	-122.9553902	2 FHA	Fraser	2
**WIC_0232**		TENTH STREET MEDICENTRE, 75 Tenth Street	New Westminster	BC	V3M 3X4	CANADA	49.2011444	-122.9161719	2 FHA	Fraser	1
**WIC_0233**		MANCHENDA MEDICAL CLINIC, 7550 King George Boulevard	Surrey	BC	V3W 2T2	CANADA	49.1396593	-122.8452616	2 FHA	Fraser	3
**WIC_0235**		THE DOCTORS' OFFICE, 101-777 West Broadway	Vancouver	BC	V5Z 4J7	CANADA	49.2635735	-123.1215328	3 VCHA	Vancouver Coastal	11
**WIC_0236**		WELCOME MEDICAL CLINIC, 105-7885 6th Street	Burnaby	BC	V3N 3N4	CANADA	49.2192009	-122.927955	2 FHA	Fraser	5
**WIC_0237**		RICHMOND CENTRAL MEDICAL CLINIC, 150-7997 Westminster Highway	Richmond	BC	V6X 1A4	CANADA	49.1702798	-123.1371078	3 VCHA	Vancouver Coastal	7
**WIC_0239**		KHAZAMIPOUR MEDICAL SERVICES, 130-8120 Cook Road	Richmond	BC	V6Y 1T9	CANADA	49.1665349	-123.134889	3 VCHA	Vancouver Coastal	1
**WIC_0240**		COOK ROAD MEDICAL CLINIC, 100-8171 Cook Road	Richmond	BC	V6Y 3T8	CANADA	49.166676	-123.1340989	3 VCHA	Vancouver Coastal	4
**WIC_0241**		VIVA CARE, 8249 Eagle Landing Parkway	Chilliwack	BC	V2R 0P9	CANADA	49.1527121	-121.978137	2 FHA	Fraser	1
**WIC_0244**		CARETEL MEDICAL CLINIC, 8488 160th Street	Surrey	BC	V4N 0Y3	CANADA	49.1562899	-122.7785511	2 FHA	Fraser	1
**WIC_0245**		SAFA MEDICAL CLINIC, 5-8948 202nd Street	Langley	BC	V1M 4A7	CANADA	49.1652982	-122.661072	2 FHA	Fraser	2
**WIC_0246**		DENNING HEALTH GROUP, 8960 152nd Street	Surrey	BC	V3R 4E4	CANADA	49.1654497	-122.8007735	2 FHA	Fraser	3
**WIC_0247**		LIVE WELL MEDICAL CENTRE, 160-8980 No. 3 Road	Richmond	BC	V6Y 2E8	CANADA	49.1488439	-123.136664	3 VCHA	Vancouver Coastal	3
**WIC_0248**		CAREPLACE MEDICAL CENTRE, 306-9014 152 Street	Surrey	BC	V3R 4E7	CANADA	49.1664629	-122.8012111	2 FHA	Fraser	3
**WIC_0249**		KAMLOOPS URGENT CARE CLINIC, 4-910 Columbia Street	Kamloops	BC	V2C 1L2	CANADA	50.6691003	-120.3562339	1 IHA	Interior	1
**WIC_0250**		HORIZON ACCESS CLINIC, 9105 Sunset Drive	Chilliwack	BC	V2P 3X8	CANADA	49.1681708	-121.9676683	2 FHA	Fraser	6
**WIC_0251**		YATES AND QUADRA INTEGRATED HEALTH CENTRE, 105-911 Yates Street	Victoria	BC	V8V 4X3	CANADA	48.4250452	-123.3576632	4 VIHA	Vancouver Island	2
**WIC_0252**		MEGA FU MEDICAL, 9251 Alderbridge Way	Richmond	BC	V6X 0N1	CANADA	49.1780884	-123.1198611	3 VCHA	Vancouver Coastal	5
**WIC_0254**		ELICARE MEDICAL, 9618 Cameron Street	Burnaby	BC	V3J 1M2	CANADA	49.2534183	-122.8979941	2 FHA	Fraser	1
**WIC_0255**		VIVA CARE, 300-9855 Austin Avenue	Burnaby	BC	V3J 1N5	CANADA	49.2512273	-122.8958631	2 FHA	Fraser	4
**WIC_0256**		HIGHROADS MEDICAL CLINICS, 177-9855 Austin Avenue	Burnaby	BC	V3J 1N4	CANADA	49.2512273	-122.8958631	2 FHA	Fraser	8
